# Causal biological network models for reactive astrogliosis: a systems approach to neuroinflammation

**DOI:** 10.1038/s41598-022-07651-0

**Published:** 2022-03-10

**Authors:** Melinda Barkhuizen, Kasper Renggli, Sylvain Gubian, Manuel C. Peitsch, Carole Mathis, Marja Talikka

**Affiliations:** PMI R&D, Philip Morris Products S.A., Quai Jeanrenaud 5, 2000 Neuchâtel, Switzerland

**Keywords:** Central nervous system infections, Cellular signalling networks, Computational biology and bioinformatics, Computational models, Neurological disorders, Alzheimer's disease

## Abstract

Astrocytes play a central role in the neuroimmune response by responding to CNS pathologies with diverse molecular and morphological changes during the process of reactive astrogliosis. Here, we used a computational biological network model and mathematical algorithms that allow the interpretation of high-throughput transcriptomic datasets in the context of known biology to study reactive astrogliosis. We gathered available mechanistic information from the literature into a comprehensive causal biological network (CBN) model of astrocyte reactivity. The CBN model was built in the Biological Expression Language, which is both human-readable and computable. We characterized the CBN with a network analysis of highly connected nodes and demonstrated that the CBN captures relevant astrocyte biology. Subsequently, we used the CBN and transcriptomic data to identify key molecular pathways driving the astrocyte phenotype in four CNS pathologies: samples from mouse models of lipopolysaccharide-induced endotoxemia, Alzheimer’s disease, and amyotrophic lateral sclerosis; and samples from multiple sclerosis patients. The astrocyte CBN provides a new tool to identify causal mechanisms and quantify astrogliosis based on transcriptomic data.

## Introduction

Astrocytes and microglia are the main neuroimmune cells of the central nervous system (CNS). In the healthy brain, astrocytes maintain ion and neurotransmitter homeostasis, provide metabolic support to neurons, and support synaptic activity^1,2^. Astrocytes also perform innate immune functions to protect the brain against pathogens. Astrocytes respond to most CNS pathologies, including traumatic brain injury, ischemic stroke, autoimmune neurological diseases, neurodegenerative diseases, microbial infections, and environmental toxin exposure. Reactive astrogliosis is characterized as a profound change in astrocyte phenotype after a CNS pathology. The pathology of reactive astrogliosis in most of these diseases has been linked to the innate immune functions of astrocytes. Astrocytes express receptors to recognize pathogen-associated molecular patterns (PAMP) and damage- or danger-associated molecular patterns (DAMP) that trigger an innate immune response. The diverse responses that can trigger these reactive astrocytes release a wide variety of effector molecules in an pathology-specific manner^3,4^.

Early transcriptome-profiling studies identified an “A1” molecular signature of 12 genes that was associated with a neurotoxic astrocyte subtype that emerged after exposure to specific cytokines secreted by microglia exposed to lipopolysaccharide (LPS), an “A2” molecular signature of 12 genes that was associated with a neurotrophic subtype after ischemic stroke, and a core set of pan-reactive genes that were not pathology-specific^2,4,5^. However, recent studies have shown that the molecular activity of astrocytes is far more heterogeneous than the A1/A2 dichotomy and even A1-like pathologies can further be subdivided into at least 9 distinct gene sets that change drastically over time^6^. Furthermore, the categories of non-immune genes that contribute to the neurotoxicity/neuroprotective effects are vast, including epigenetic modifiers^7^, genes which encode toxic saturated lipids^8^, and genes which influence astrocyte-microglia communications^9^. Given this broad heterogeneity, there is a need for a tool to distinguish the molecular mechanisms driving the astrocyte phenotype in different CNS pathologies.

Our framework utilizes an established causal biological network (CBN) model methodology^10–14^. CBNs integrate a collection of causal statements curated from the scientific literature into a coherent computable network, which can aid in the interpretation of large-scale transcriptomic changes. The nodes within the network represent biological entities, such as mRNA, proteins, protein activities, and biological processes. The nodes are connected by edges indicating directional relationships (i.e., increases or decreases), which gives the network a cause-and-effect backbone topology. A second scorable layer is generated by using known gene expression signatures associated with a subset of nodes in the network and comparing the signatures of these nodes to pathology-induced changes in gene expression values to infer the activity of the node in a dataset. This approach considers changes in the abundance of transcripts as a downstream response to node activity without assuming these transcripts produce active proteins. In this way, a single transcriptomic profile (instead of many measurements at different biological levels) can be used to determine the activity of the whole network^11,14^.

In this study, we present a CBN model that describes important molecular events involved in astrocyte activity. We also show how transcriptomic data from models of amyotrophic lateral sclerosis (ALS), neuroinflammation caused by systemic LPS-induced endotoxemia, Alzheimer’s disease (AD), and multiple sclerosis (MS) can be assessed with this network model to get a mechanistic understanding of gene expression changes. This model is stored in the CBN database (http://www.causalbionet.com/) and accessible to the public.

## Methods

### General approach

Biological Expression Language (BEL, www.openbel.org, RRID:SCR_017661) is a human-readable, computable language that captures causal and correlative biological observations in statements consisting of two biological entities (nodes) connected by a relationship (edge). The gene signatures of the A1 vs. A2 astrocyte reactivity paradigm served as a starting point for identifying relevant pathways involved in astrogliosis^4,15^. We searched the literature for mechanistic papers published between 1994 and 2021 that described causal relationships between proteins encoded by the genes listed in the molecular signature of the A1 neuroinflammatory response induced by the bacterial toxin LPS, the A2 neurotrophic response induced by ischemic stroke, and the pan-reactive astrogliosis response^4,15^. These articles were supplemented with additional publications on astrogliosis in other CNS and retinal diseases also published up to 2021. The criteria to select publications were as follows: (1) The article was an original research article with a PubMed identifier, and (2) it studied the manipulation of astrocytes in a healthy or a non-cancerous CNS disease-related context. This led to the inclusion of studies using human biopsies, in vivo studies with mice and rats, and in vitro studies from primary astrocytes, organotypic slice cultures, and induced pluripotent stem cell-derived astrocytes. Mechanistic information originating from glioblastoma studies, other cancers, or immortalized cell lines were not included in the CBN. The journal impact factor or any other means of ranking publications was not considered. If the statements in the original research article were sufficiently supported by the results presented in the figures, the information was considered reliable and captured^16^. To retrieve causal relationships, the result sections from these articles were extracted for curation. Contradicting statements were captured without preferential treatment and with proper annotations (model organism, tissue, cell line, and treatment/disease). BEL statements curated from the literature were compiled into a CBN backbone by using the OpenBEL framework 3.0.0, an open-source compilation framework^16^. The CBN consists of biological entities (nodes) and the relationships between them. The network was visualized in Cytoscape (version 3.7.1, RRID:SCR_003032); the node and edge counts and most connected nodes in the network were obtained using the Network Analyzer tool in Cytoscape^17^.

### Data-driven expansion of the CBN model

The literature-derived CBN model backbone was expanded using a data-driven approach to identify additional molecular drives in astrogliosis that might have been missed during literature curation. The approach utilized a database of nearly 800 nodes with inferable activities (iNodes). The activity of these nodes in an experimental dataset can be inferred by comparing the differential mRNA abundances in the dataset with the known mRNAs regulated by the iNodes. The regulated mRNAs by the iNodes were derived from public datasets of mRNA changes in response to manipulation of the nodes (i.e., by knockdown, overexpression, inhibition, etc.). iNodes were inferred from transcriptomic data (GSE35338) published by Zamanian et al.^4^. GSE35338 contains microarray data from A1 astrocytes extracted from young adult male mice 1 day after intraperitoneal injection of 5 mg/kg LPS to produce neuroinflammation and from A2 astrocytes that were purified from young adult male mice 1, 3, or 7 days after induction of middle cerebral artery occlusion (MCAO) to induce ischemic stroke. After scoring of the iNode database with transcriptomic data from treatment versus control samples, we selected iNodes with an adjusted p-value < 0.01 for data-driven network enhancement. Additional literature was identified to find evidence to support the addition of new nodes into the networks using the same criteria as those for publications selected for construction of the CBN backbone.

### Identification of molecular drivers with the iNode approach

**Figure 1:**
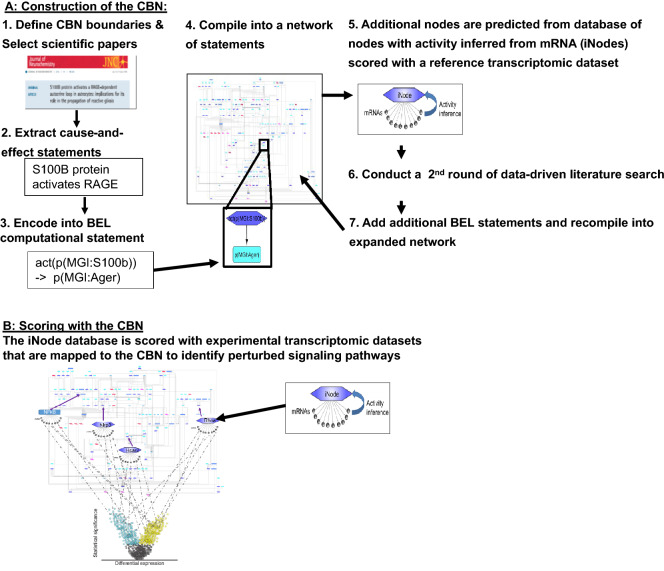
Overview of the causal biological network (CBN) approach. *BEL* Biological Expression Language.

We used four datasets that were not used for the data-driven network expansion to gain a mechanistic understanding of astrogliosis following different pathologies. The public datasets were: (i) GSE75246: astrocytes, neurons, and microglia from a mouse model of systemic LPS-induced endotoxemia vs. vehicle treated control cells^18^; (ii) GSE75431: astrocytes from the PS2APP mouse model of Alzheimer’s disease (AD) vs. non-trangenic controls^18^; (iii) GSE83670: astrocytes isolated from the normal-appearing white matter of human multiple sclerosis (MS) patients vs. astrocytes from controls^19^; and (iv) GSE69166: spinal cord astrocytes from the SOD1 G93A transgenic mouse model of ALS vs. nontransgenic controls^20^. The transcriptomic data were scored against the iNode database, and the iNodes with an adjusted inferred p-value < 0.01 were selected. The scoring results were uploaded to Cytoscape^17^ to visualize the iNode enrichment results in the context of the astrocyte CBN. The network model was orthologized to the equivalent mouse or human gene symbols to allow the mapping of the inferred nodes onto the network. Composite images for publication were generated with Inkscape (version 0.92.4, RRID:SCR_014479). An overview of our approach is shown in Fig. 1.

## Results

### Model description

We initially constructed three CBNs to capture A1/A2 and pan-reactive astrocyte activity. The literature backbone of the A1 astrocyte activity CBN consisted of 401 nodes connected by 732 edges, supported by data from 62 publications. After data-driven expansion, the A1 CBN was supported by data from 76 relevant peer-reviewed articles. The expanded A1 network contained 502 nodes and 893 edges. The literature backbone of the A2 astrocyte activity CBN consisted of 334 nodes connected by 504 edges, supported by data from 43 publications. After data-driven expansion, the A2 astrocyte activity network model was computed from 81 publications and consisted of 535 nodes and 842 edges. The literature backbone of the pan-reactive astrocyte CBN consisted of 458 nodes connected by 816 edges, supported by data from 70 publications. After data-driven expansion, the pan-reactive astrocyte activity network model was computed from 123 publications and contained 879 nodes and 1587 edges. However, several mechanisms and publications overlapped between the networks, and given the heterogeneity of reactive astrocyte activities^6–9^, we merged all three networks into one comprehensive network of astrocyte reactivity and updated the merged network with additional literature references.

The final merged astrocyte activity network model was computed from 235 publications (Supplementary List 1) and contained 1102 nodes and 2487 edges (Supplementary Fig. 1). The nodes with the most outdegree edges were the upstream inflammatory receptors TLR4 (Toll-like Receptor 4; 7 indegree edges and 60 outdegree edges), the IL-1 (Interleukin-1) receptor complex (3 indegree edges and 55 outdegree edges), and the TNFRSF (Tumor Necrosis Factor Receptor Superfamily; 1 indegree edges and 55 outdegree edges). The downstream nodes with the most indegree edges were the NF-κB complex (Nuclear Factor-Kappa B, 58 indegree edges and 40 outdegree edges) and GFAP (Glial Fibrillary Acidic Protein, 31 indegree edges and 2 outdegree edges).

### Drivers of astrocyte activity in a mouse model of endotoxemia^18^

For the first analysis, we assessed GSE75246, an RNA-sequencing dataset of astrocytes, neurons, and microglia isolated from the hemicortex of 2- to 2.5-month-old male mice 24 h after intraperitoneal injection of LPS or vehicle^18^. This dataset enabled us to explore the cellular specificity of the three main cell types that compose the brain with respect to their transcriptional response to an endotoxin insult. Systemic LPS injection is known to cause robust neuroinflammation^4^.

When the iNode enrichment results were visualized in the context of the astrocyte CBN, we identified a set of 46 connected nodes, mapped to different signaling pathways in the CBN. The first subset captured nodes related to inflammatory processes. As expected, LPS and the TLRs (TLR2, TLR3, TLR4, TLR5, and TLR7); their downstream effector MYD88 (MYD88 Innate Immune Signal Transduction Adaptor); NF-κB complex; and the IκKB (Inhibitor of NF-κB kinase) complex were inferred to be upregulated in astrocyte and microglia samples. Only TLR2 and IκKB were inferred to be upregulated in neurons.

The type I and II interferons and their receptors (IFNG, IFNB1, IFNAR1); Interferon regulatory factors (IRF1, IRF3); TNF-α (Tumor Necrosis Factor Alpha); the interleukin (IL)-6 family members IL-6, OSM (Oncostatin M), and LIF (Leukemia Inhibitory Factor); IL-19; IL-27; IL-1B and the IL-1 receptor complex were also inferred to be upregulated in astrocyte and microglia samples. Only LIF, IL-27, and IL-19 were inferred to be upregulated in neurons.

In addition to the inflammatory signaling pathways, proteins/chemicals/toxins associated with neurodegenerative diseases (beta-amyloid, *N*-methyl-4-phenylpyridium, and Apolipoprotein E (APOE)) were inferred to be upregulated in astrocytes and microglia. Other inferred upregulated signaling pathways in astrocytes and microglia included the p38 MAPK (Mitogen-Activated Protein Kinase) family; CSF2 (Colony Stimulating Factor 2); and FFAR3 (Free Fatty Acid Receptor 3).

**Figure 2:**
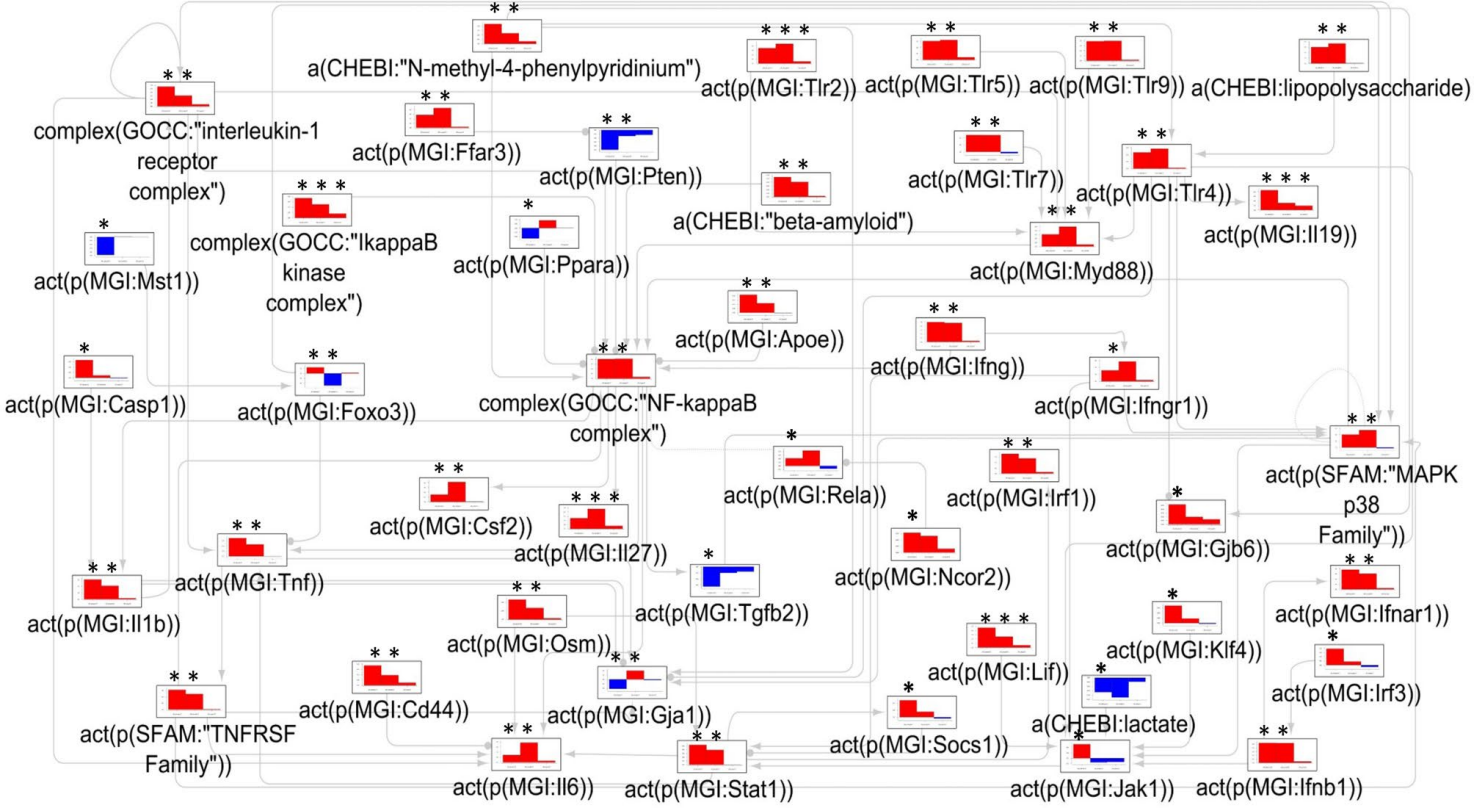
The mechanistic network of dysregulated signaling cascades in LPS-induced endotoxemia. The bar plots show the inferred scores of datasets derived from (i) astrocytes (left side bar plots), (ii) microglia (bar plots in the middle), and (iii) neurons (right side bar plots) isolated from LPS-treated mice in comparison to those from the corresponding cells isolated from vehicle-treated mice. Red and blue indicate inferred up- and downregulation, respectively. The values are not comparable between the graphs, and the scale was adjusted based on the highest value for each node. Inferred adjusted p-values < 0.01 are indicated by an Asterisk (*). Arrow legends: → causes an increase in. *a(…)* abundance, *act(p(…))* protein activity, *CHEBI* Chemical Entities of Biological Interest database, *complex(…)*, activity of a protein complex, *GOCC* Gene Ontology Cellular Complex database, *LPS* lipopolysaccharide, *MGI* Mouse Genome Informatics database.

PTEN (Phosphatase and Tensin Homolog) was inferred to be downregulated in astrocyte and microglia samples. The JAK1 (Janus Kinase 1)—STAT1 (Signal Transducer and Activator Of Transcription 1) pathway and the SOCS1 (Suppressor Of Cytokine Signaling 1), which inhibits STAT1 signaling cascade; caspase 1 (CASP1), which formed part of the NLRP3 (NOD-, LRR- and pyrin domain-containing protein 3) inflammasome together with IL-1B; the connexin-30 hemichannel (GJB6); the IFNGR1 (Interferon Gamma Receptor 1); and the RelA p65 subunit of the NF-κB complex were inferred to be upregulated in astrocytes, but not in microglia. The FOXO3 (Forkhead Box O3) cellular survival pathway was inferred to be upregulated in astrocyte samples but downregulated in microglia. PPARA (Peroxisome Proliferator-Activated Receptor (PPAR) Alpha) and the connexin-43 hemichannel (GJA1) were inferred to be downregulated in astrocytes and upregulated in microglia. Lactate and TGFB1 (Transforming Growth Factor Beta 1) were inferred to be downregulated in astrocytes but unchanged in microglia samples. An overview is given in Fig. 2 and Supplementary Table 1.

### Drivers of astrocyte activity in the PS2APP mouse model of Alzheimer’s disease^18^

The GSE75431 RNA-sequencing dataset contained the transcriptome of astrocytes isolated from the whole cortex of 7- and 13-month-old PS2APP transgenic mice and their non-transgenic littermates^18^. The PS2APP transgenic mouse is a model of AD characterized by the overexpression of the mutant alleles of amyloid precursor protein (APP) and presenilin (PSEN2), which induce an age-dependent beta-amyloid plaque pathology associated with astrogliosis in the hippocampus and frontolateral cortex^21^.

The analysis of the significantly scored iNodes in the context of the CBN model resulted in a smaller set of connected nodes (9 nodes) in the AD dataset than in the LPS dataset. After the inclusion of the NF-κB node (scoring did not reach statistical significance), which was the most connected node in the CBN, 21 nodes were connected in the samples collected from 13-month-old animals. Most of these nodes were not significant in samples from the 7-month-old mice. The connected subgraph highlighted inferred decreases in dexamethasone and the NR3C1 glucocorticoid receptor; IL-6; the metabolic regulators PPARG and PPARGC1A (PPARG Coactivator 1 Alpha/PCG-1α); the mitochondrial protein kinase PINK1 (PTEN Induced Kinase 1); and the antioxidant NFE2L2 (Nuclear factor erythroid 2/NRF2).

**Figure 3:**
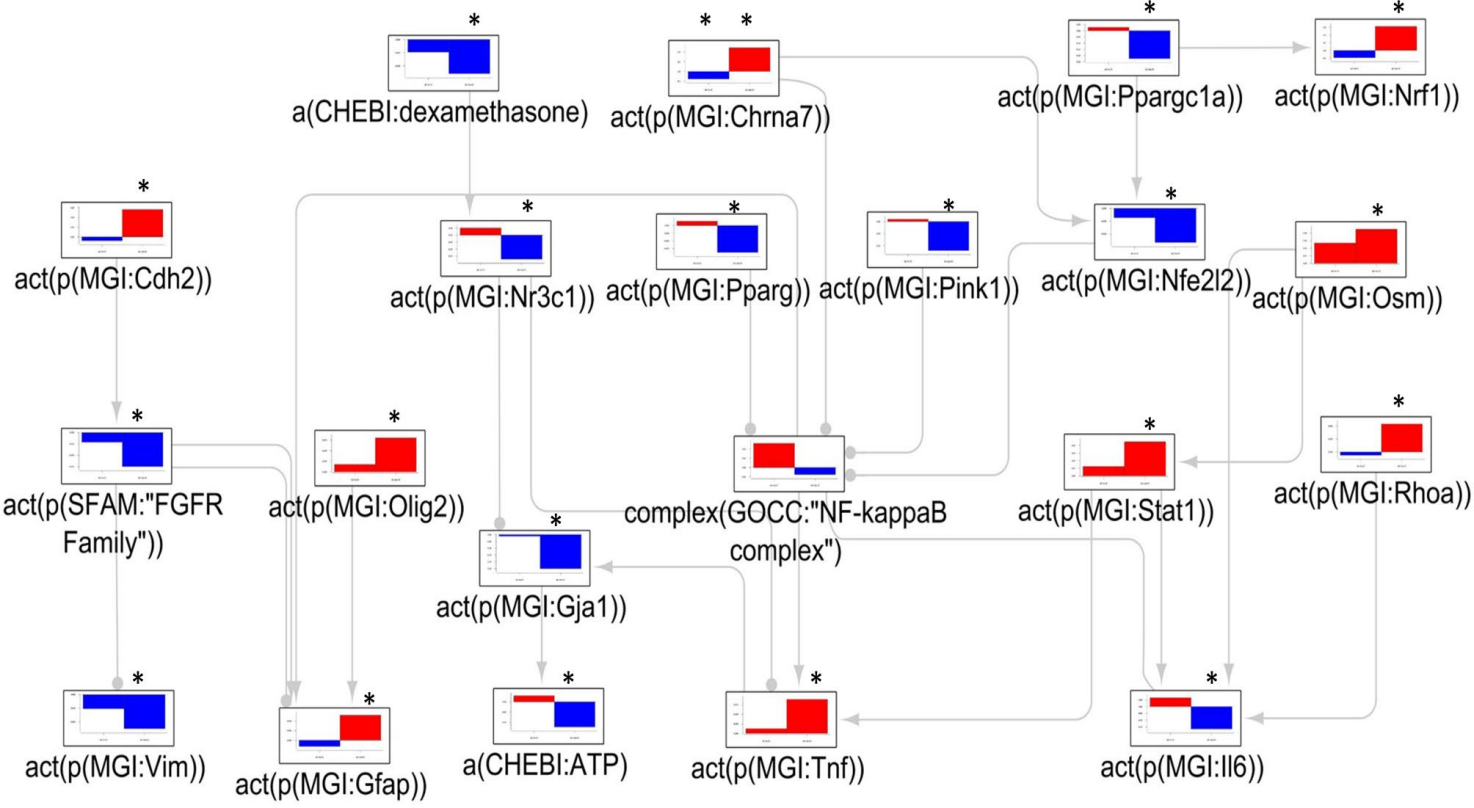
The mechanistic network of dysregulated signaling cascades in Alzheimer’s disease. The bar plots show the inferred scores computed following analysis of the dataset derived from astrocytes isolated from (i) 7-month-old transgenic AD mice vs. non-transgenic controls (left side bar plots) and (ii) 13-month-old transgenic AD mice vs. non-transgenic control mice (right side bar plots). Red and blue indicate inferred up- and downregulation, respectively. The values are not comparable between the graphs, and the scale was adjusted based on the highest value for each node. Inferred adjusted p-values < 0.01 are indicated by an Asterisk (*). Arrow legends: → causes an increase in; -● causes a decrease in. *AD* Alzheimer`s disease, *a(…)* abundance, *act(p(…))* protein activity, *CHEBI* Chemical Entities of Biological Interest database, *complex(…)* activity of a protein complex, *GOCC* Gene Ontology Cellular Complex database, *MGI* Mouse Genome Informatics database.

NRF1 (Nuclear Respiratory Factor 1); CHRNA7 (Cholinergic Receptor Nicotinic Alpha 7 Subunit); TNF-α and STAT1 were inferred to be upregulated. CHD2 (Chromodomain Helicase DNA Binding Protein 2) was inferred to be upregulated, but its downstream signaling through the FGFR (Fibroblast growth factor receptor) family, as well as the structural protein VIM (Vimentin), downstream of the FGFR family was inferred to be downregulated. Other structural markers, GFAP and OLIG2 (Oligodendrocyte Transcription Factor 2), as well as the cell-shape regulator RHOA (Ras homolog family member A) were inferred to be upregulated. An overview of these signaling cascades is shown in Fig. 3 and Supplementary Table 2.

### Drivers of astrocyte activity in patients with multiple sclerosis^19^

MS is an autoimmune disease with a strong inflammatory component characterized by demyelinated white-matter lesions in the brain and spinal cord. The white matter lesions are surrounded by areas of non-demyelinated normal-appearing white matter^19^. The GSE83670 dataset compared Affymetrix microarray gene expression data from human astrocytes isolated by postmortem laser-capture microdissection from the normal-appearing white matter of MS patients and neurologically normal age- and sex-matched controls. The authors of this dataset studied astrocytes isolated from the normal-appearing white matter, rather than those from the lesion area, to identify factors that could initiate or prevent MS progression^19^.

**Figure 4:**
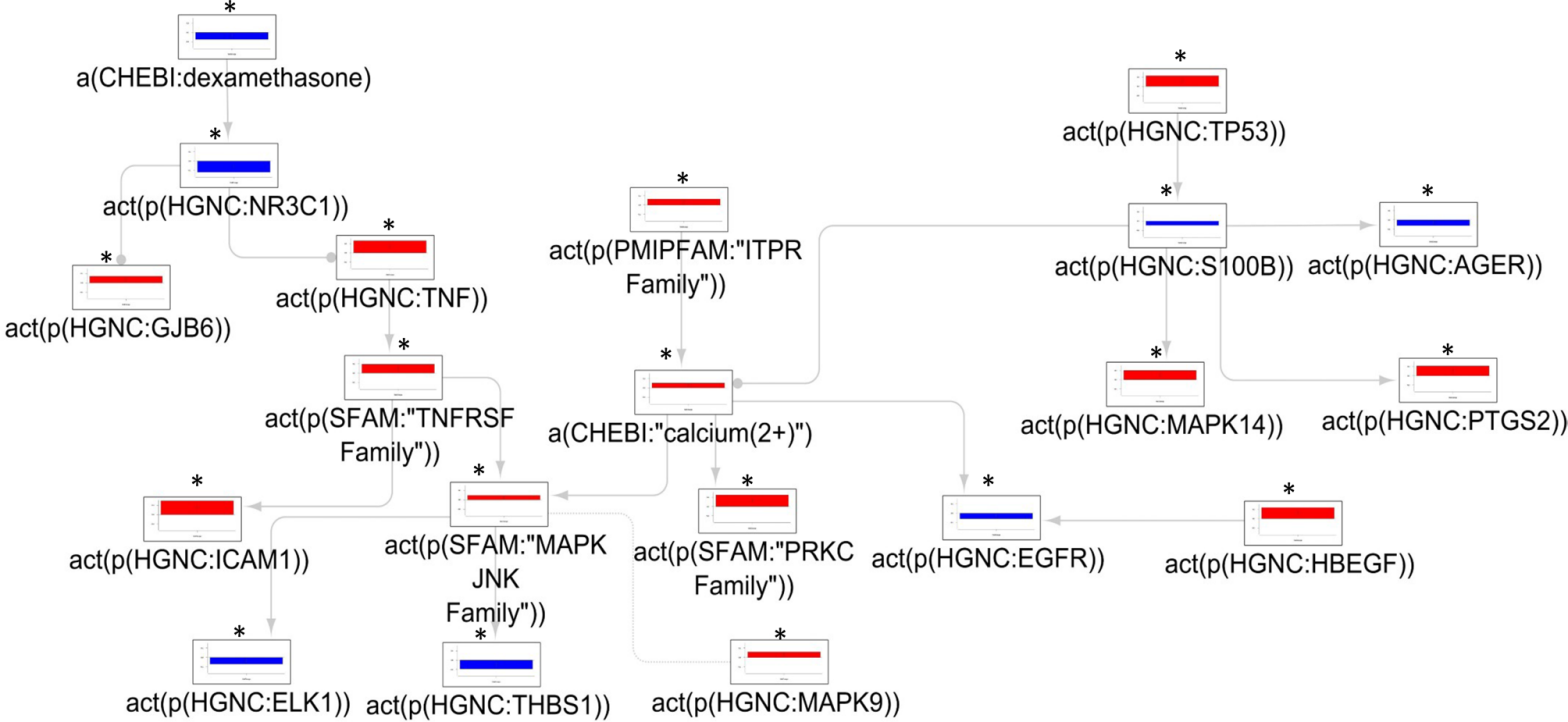
The mechanistic network of dysregulated signaling cascades in astrocytes isolated from MS patients versus control subjects. The bar plots show the inferred scores computed following analysis of the dataset derived from astrocytes isolated from the normal-appearing white matter of human MS patients vs. that of astrocytes from neurologically normal controls. Red and blue indicate inferred up- and downregulation, respectively. Inferred adjusted p-values < 0.01 are indicated by an Asterisk (*). Arrow legends: → causes an increase in; -● causes a decrease in. *a(…)* abundance, *act(p(…))* protein activity, *CHEBI* Chemical Entities of Biological Interest database, *complex(…)* activity of a protein complex, *GOCC* Gene Ontology Cellular Complex database, *HGNC* Hugo Gene Nomenclature database, *MS* multiple sclerosis.

The iNode enrichment analysis highlighted a set of 20 connected nodes in the astrocyte CBN. The anti-inflammatory glucocorticoid pathway (dexamethasone and the glucocorticoid receptor NR3C1) was inferred to be downregulated. TNF-α and the TNF receptor superfamily; downstream MAPK JNK (c-Jun N-terminal kinases) family; MAPK9; and MAPK14 were inferred to be upregulated; along with connexin-30 (GJB6). The ITPR (Inositol Triphosphate Receptor)—calcium—PRKC (Protein Kinase C) signaling pathway was also inferred to be upregulated. S100B (S100 Calcium Binding Protein B) and its receptor AGER (Advanced Glycosylation End-Product Specific Receptor) were inferred to be downregulated, but the nodes up- and downstream from S100B, TP53 (Tumor Protein P53), and PTGS2 (Prostaglandin-endoperoxide Synthase 2/Cyclooxygenase-2), were inferred to be upregulated. Other inferred upregulated nodes included the ICAM1 (Intercellular Adhesion Molecule 1), and HBEGF (Heparin-Binding Epidermal Growth Factor-like). The EGFR (Epidermal Growth Factor Receptor), downstream of HBEGF, was inferred to be downregulated along with THBS1 (Thrombospondin 1) and ELK1 (ETS Transcription Factor ELK1). An overview of these changes is given in Fig. 4 and Supplementary Table 3.

### Drivers of spinal cord astrocyte activity in from the SOD1 mouse model of ALS^20^

ALS is the most common motor neuron disease in adults. It leads to the death of motor neurons, which causes progressive paralysis. Approximately 10% of ALS cases are familial, and mutations in the superoxide dismutase 1 (SOD1) gene account for approximately 15% of familial ALS cases^22^. We analyzed the GSE69166 dataset, which used an Affymetrix microarray to compare gene expression in astrocytes isolated by laser-capture microdissection from the spinal cord of 3- or 4-month-old SOD1 G93A ALS mice with those isolated from their non-transgenic littermates. In this mouse model, pre-symptomatic ALS occurred at the age of 2 months, symptomatic ALS at 3 months, and late-stage ALS at 4 months after birth. The authors showed that the astrocytes isolated from the SOD1 transgenic mice had an altered immune response, with increased expression of lysosomal, phagocytic, and chemokine genes starting at the symptomatic ALS stage and continuing through late-stage ALS. The SOD1 transgenic astrocytes also showed a reduction in homeostatic transcripts involved in potassium transport. With functional assays for the activity of the lysosomal gene β-hexosaminidase and the uptake of neuronal cellular debris, the authors confirmed that lysosomal enzyme activity and phagocytosis were increased in the SOD1 transgenic astrocytes^20^.

In the late stage of ALS, we identified a set of 51 significant nodes connected by 64 edges. The first mechanism highlighted was a general increase in immune and inflammatory signaling. The toll-like receptors (TLR2, TLR3, TLR4, TLR5, and TLR7) and their downstream effector MYD88 were inferred to be upregulated. Other immune and inflammatory factors, including a set of type I and II interferons and their receptors (IFNG, IFNGR, IFNB1, IFNAR1); IRF3; PTGS2; TNF-α and the TNF receptor superfamily; and LIF were also inferred to be upregulated, as well as chemicals/toxins associated with neurodegenerative diseases (beta-amyloid and N-methyl-4-phenylpyridium), whilst the anti-inflammatory glucocorticoid receptor (NR3C1) and its ligand (dexamethasone) were inferred to be downregulated.

**Figure 5:**
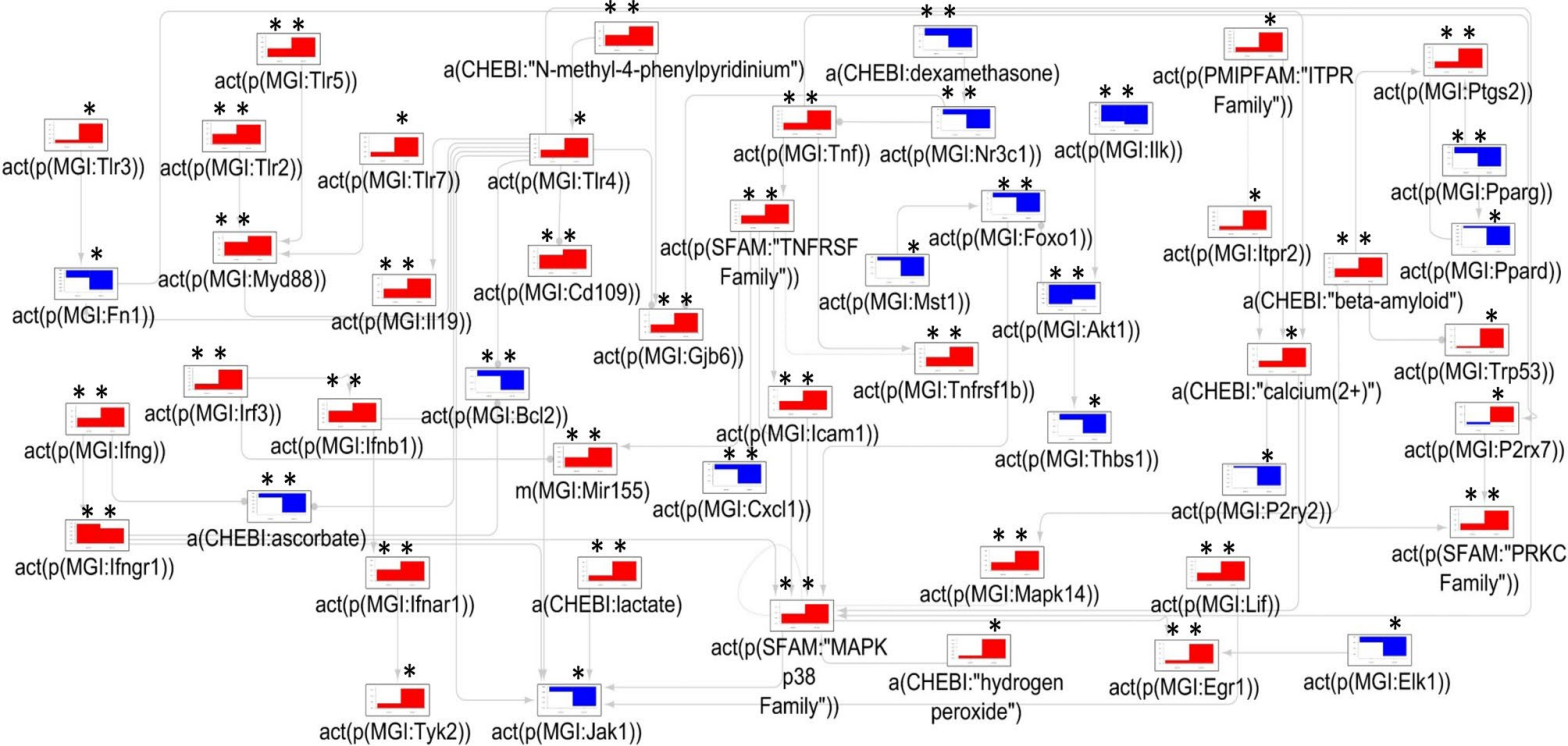
The mechanistic network of dysregulated signaling cascades in late-stage ALS. The bar plots show the inferred scores of datasets derived from (i) astrocytes isolated from symptomatic transgenic ALS mice vs. non-transgenic controls (left side bar plot) and (ii) late-stage stage ALS mice vs. non-transgenic controls (right side bar plot). Red and blue indicate inferred up- and downregulation, respectively. The values are not comparable between the graphs, and the scale was adjusted based on the highest value for each node. Inferred adjusted p-values < 0.01 are indicated by an Asterisk (*). Arrow legends: → causes an increase in; -● causes a decrease in. *act(…)* protein activity, *ALS* amyotrophic lateral sclerosis, *a(…)* abundance, *act(p(…))* protein activity, *CHEBI* Chemical Entities of Biological Interest database, *complex(…)* activity of a protein complex, *GOCC* Gene Ontology Cellular Complex database, *MGI* Mouse Genome Informatics database.

The network also highlighted increased oxidative stress, with an inferred upregulation of the pro-oxidant hydrogen peroxide and downregulation of the antioxidant molecule ascorbate. The metabolic regulators PPAR family members PPARD and PPARG were inferred to be downregulated. Other inferred upregulated signaling pathways included the p38 MAPK family; ITPR and the purinergic receptors (P2RY2 and P2RX7) which converge downstream on calcium and PRKC signaling. The ILK1 (Integrin-Linked Kinase)—AKT1 (AKT serine/threonine-protein kinase)—FOXO1 (Forkhead Box O1) cellular survival pathway was inferred to be downregulated. In general, the directionality of the regulation was similar in the earlier stage of ALS, but their inferred fold-changes were smaller. An overview of the dysregulated signaling pathways is shown in Fig. 5 and Supplementary Table 4.

## Discussion

Reactive astrogliosis plays a central role in CNS diseases and injuries. Traditionally, reactive astrogliosis has been identified by astrocyte hypertrophy and increased expression of GFAP. Later, a framework of neurotoxic A1 astrocytes, neurotrophic A2 astrocytes, and general pan-reactive astrocytes emerged^4^. However, recent studies have shown that astrogliosis is a much more complex and highly heterogeneous process^6–9,23^, and the molecular signature of reactive astrogliosis is strongly dependent on the type of injury^4^. Here, we introduced a comprehensive CBN model to aid in the identification of relevant molecular processes related to astrogliosis in high-throughput datasets. Scientific statements derived from original research articles and scripted into BEL were assembled into a network model that captured the molecular processes involved in diverse types of astrocyte activation^4,15^. Although the network was constructed to capture both the neurotoxic and neurotrophic aspects of astrocyte activation, the most connected nodes in the network were related to pro-inflammatory processes and the astrocytic marker GFAP.

We used the iNode enrichment analyses to infer the molecular drivers of reactive astrogliosis in four datasets: an LPS-induced mouse model of endotoxemia-associated neuroinflammation, the PS2APP mouse model of AD, the SOD1 G93A mouse model of ALS, and astrocytes isolated from the normal-appearing white matter of human patients with MS. The iNode analysis results mapped to the astrocyte CBN indicated that astrocytes isolated from both the ALS and LPS models had a highly inflammatory phenotype. Downstream, there was a divergence of the master-inflammatory regulator NF-κB. The NF-κB node reached significance in astrocytes from the LPS-induced inflammation dataset, but not in the ALS, AD, or MS datasets. The analysis also implicated downstream inflammatory mediators that overlapped between LPS and the neurodegenerative disease datasets. The p38 MAPK family, which is associated with several stages of the inflammatory process in neurodegenerative diseases^24^, was upregulated in ALS and LPS-treated mice. STAT1 was perturbed in LPS-treated and AD mice. The JAK/STAT signaling pathway is downstream of several cytokines and interferons, where it plays a role in immune functions and contributes to neuroinflammation^25^. Chemicals/proteins associated with neurodegenerative diseases (the Parkinson’s disease environmental toxin N-methyl-4-phenylpyridium and the AD-associated protein beta-amyloid), were inferred to be upregulated in the LPS-treated and ALS, but not in AD or MS astrocytes.

The structural astrocyte marker GFAP and the transcription factor OLIG2 were inferred to be upregulated in late-stage AD, and vimentin was inferred to be downregulated in the AD mouse astrocytes. GFAP and vimentin are the two major intermediate filament proteins in astrocytes, and together, are key components for glial scar formation after injury^26^. OLIG2 is a transcription factor that plays an essential role in the differentiation of oligodendrocytes and neurons in the developing CNS. However, OLIG2 also has a role in astrocyte differentiation. While most astrocytes express GFAP, there are GFAP-negative astrocytes in the CNS. A proportion of these GFAP-negative astrocytes are positive for OLIG2. These astrocytes play a role in inhibitory neuronal transmission^27^. These structural proteins are often used in immunohistochemistry studies to characterize astrocytes because they label a large part of the injury and are expressed at higher levels after CNS injury^28^. The inferred changes in these marker proteins could reflect changes in either astrocyte morphology, or astrocyte numbers, as the formation of the glial scar involves both. The publication associated with GSE75246 did not show the morphology of the astrocytes^18^, however, the inferred increase of GFAP and OLIG2 in our analysis suggests changes in astrocyte morphology.

The nodes inferred to be regulated in the astrocytes isolated from normal-appearing white matter in MS patients partially overlapped with the inflammatory and anti-inflammatory nodes highlighted in the other datasets. In addition, EGFR, S100B, and AGER were inferred to be decreased in MS. S100B is a cytoplasmic protein primarily expressed by astrocytes^28^. S100B is a calcium-binding protein that can be released in the extracellular space from injured cells and its high serum level is usually a sign of CNS injury. AGER (also known as RAGE) is a pattern recognition receptor. S100B mediated activation of AGER activates an inflammatory phenotype that induces NF-κB signaling^29^. There is a feedback loop between S100B, and TP53, where S100B reduces the activity of TP53 in a calcium-dependent manner, and the S100B/TP53 interaction mediates the effects of S100B^30,31^. TP53 promotes astrocyte maturation, reduces proliferation, and enhances the expression of synapse-associated genes, whilst HBEGF-EGFR signaling forms a positive feedback loop which inhibits astrocyte maturation, promotes proliferation, and reduces the expression of synapse forming genes. The inferred reduced expression of EGFR and increased expression of TP53 could indicate a reduction in proliferation and increased maturation of astrocytes in MS. The predicted directionality of HBEGF was inconsistent with the directionality of EGFR, however, both HBEGF and EGFR are also involved in other signaling pathways apart from the pathways captured in the CBN^32^, possibly explaining the causal inconsistency. The MS astrocytes appeared to be less reactive in certain reactive astrocyte pathways, with decreases in the S100B-AGER inflammatory axis. The anti-inflammatory signaling through the glucocorticoid receptor was inferred to be downregulated, whilst inflammatory signaling through TNF-α and its receptor was inferred to be upregulated. The authors of the publication associated with the GSE83670 dataset proposed that these astrocytes played a neuroprotective role in MS^19^.

In summary, we demonstrated how the reactive astrocyte CBN can be used in combination with the iNode enrichment analysis to identify drivers of astrocyte activation resulting from CNS pathologies. The current approach has some limitations. In the above analyses, we have limited the discussion to the connected nodes, however, several other network nodes were inferred to be regulated based on the transcriptomic datasets we used (shown in the Supplementary Information). It is likely that by focusing on sets of connected nodes, we missed some interesting candidate factors which were not connected to other perturbed nodes. Secondly, the mechanistic information extracted from transcriptomic data is limited to the extent of the iNode database. The inclusion of nodes in our scoring database is limited by the availability of high-quality public datasets in which these nodes were manipulated. Moreover, the biological context in public datasets varies and is often not astrocyte-specific. Hence, the mRNAs regulated by these nodes might be different in an astrocyte-specific context than in a general biological context. During the construction and data-driven expansion of the CBN, we were limited to pathways described in an astrocyte-specific context in the literature. As a result, not all the nodes suggested during data-driven expansion could be substantiated and there are likely parts of the signaling pathways missing. These nodes can be added to the model after experimental verification as the strength of the astrocyte-specific literature evidence grows. Lastly, while the use of the CBN tool helps to identify drives of astrocyte phenotypes, it will be worth further validating them with experimental data to support conclusions about the underlying disease biology.

## Conclusions

In conclusion, because reactive astrogliosis is a highly heterogeneous process, representation of the diverse mechanisms in astrocyte activity processes through CBN models can be a powerful tool for systems medicine^13,33^. In this manuscript, we demonstrated that the astrocyte activation network can be used as a substrate for scoring high-throughput data to gain a disease-specific mechanistic understanding of the differences between diseased and healthy astrocytes and different stages of the disease. Once more transcriptomic data become available, the model can be used to assess gene expression profiles in individuals with CNS diseases for classifying subjects, generating hypotheses about the mode of action of novel drug candidates, and predicting the drivers of treatment outcomes. Ultimately, the astrocyte network model could provide a supporting tool for mechanistic disease investigation, tailored drug therapy, and precision medicine.

## Supplementary Information

Supplementary Information.

## Data Availability

Transcriptomic datasets used are available in the Gene Expression Omnibus database on (www.ncbi.nlm.nih.gov/geo/). CBN models are available on the Causal Biological Networks database (www.causalbionet.com).
