# Supplementary material for: Causal biological network models for reactive astrogliosis: a systems approach to neuroinflammation
Source: Sci Rep. 2022 Mar 10;12:4205. doi: 10.1038/s41598-022-07651-0 (PMC8913664; doi:10.1038/s41598-022-07651-0)
Supplement: Supplementary file 1 — Supplementary Information. [file 41598_2022_7651_MOESM1_ESM.docx]

**ADDITIONAL FILE**

**Supplementary data**

**Causal biological network models for reactive astrogliosis: A systems approach to neuroinflammation**

Melinda Barkhuizen, Kasper Renggli, Sylvain Gubian, Manuel C. Peitsch, Carole Mathis, and Marja Talikka

PMI R&D, Philip Morris Products S.A., Quai Jeanrenaud 5, CH-2000 Neuchâtel, Switzerland

**Supplementary Table 1. Significant iNodes in astrocytes isolated from astrocytes of mice with neuroinflammation due to lipopolysaccharide (LPS) treatment, compared to vehicle treated samples scored with the reactive astrocyte CBN. The values of the same iNodes in LPS-treated microglia and neuron samples are also shown.**

| iNode name | Astrocyte fold-change | Astrocyte adjusted p value | Microglia fold-change | Microglia adjusted p value | Neuron fold-change | Neuron  adjusted p value |
| --- | --- | --- | --- | --- | --- | --- |
| a(CHEBI:"17alpha-estradiol") | 0.1275875322 | 0.008122449 | -0.0197588095 | 1.0 | 0.0286658597 | 0.1057622378 |
| a(CHEBI:"beta-amyloid") | 0.1364097762 | 1.5E-9 | 0.1023405906 | 0.0 | 0.0055283965 | 0.4673657143 |
| a(CHEBI:"N-methyl-4-phenylpyridinium") | 1.1177631663 | 0.0 | 0.5895863371 | 0.0 | 0.1342158723 | 0.0737037037 |
| a(CHEBI:"poly(I:C)") | 0.4319739523 | 0.0 | 0.2954040838 | 0.0 | 0.0276497526 | 0.1467486034 |
| a(CHEBI:"sphingosine 1-phosphate") | 0.1231996467 | 0.008122449 | 0.2211683706 | 0.0984065934 | -0.0342458756 | 0.0241212121 |
| a(CHEBI:dexamethasone) | -0.1565027316 | 0.0 | -0.0379929197 | 0.2993504274 | 0.0138560985 | 0.2944739884 |
| a(CHEBI:ethanol) | 0.2826026707 | 0.0 | -0.0158015118 | 1.0 | 0.1466774467 | 0.0 |
| a(CHEBI:lactate) | -0.0833459 | 0.0043497268 | -0.1138140621 | 0.0122934363 | -0.0231747214 | 0.1487032967 |
| a(CHEBI:lipopolysaccharide) | 0.2995616396 | 0.0 | 0.3652406676 | 0.0 | 3.787528E-4 | 0.6578264151 |
| act(p(MGI:Ache)) | -0.3126105204 | 0.0 | -0.1481841595 | 0.0 | -0.023563915 | 0.2219330855 |
| act(p(MGI:Akt2)) | 0.3916648543 | 0.0 | 0.3183981371 | 0.0034759825 | 0.0552675858 | 0.0 |
| act(p(MGI:Akt3)) | 0.1834885536 | 0.0 | 0.2141147315 | 0.0350440252 | 0.0517897206 | 1.0E-10 |
| act(p(MGI:Apc)) | -0.1407126223 | 0.0043497268 | -0.020861733 | 0.0390419762 | -0.0571570297 | 0.0 |
| act(p(MGI:Apoe)) | 0.2115135471 | 0.0 | 0.1112944324 | 3.0E-10 | -0.0039358119 | 0.3555119826 |
| act(p(MGI:Casp1)) | 0.1656149634 | 0.0043497268 | 0.022164847 | 0.0632117647 | -0.0042867867 | 0.377468254 |
| act(p(MGI:Casp7)) | -0.1771170812 | 0.0 | -0.1680064299 | 0.0 | -0.006899097 | 0.4808489796 |
| act(p(MGI:Cd40)) | 0.4141795655 | 0.0 | 0.3313870824 | 0.0 | 0.0163646931 | 0.2950689655 |
| act(p(MGI:Cd44)) | 0.186836508 | 0.0 | 0.0960392648 | 0.0122934363 | 0.0245625636 | 0.1981877551 |
| act(p(MGI:Csf2)) | 0.1498041781 | 0.0043497268 | 0.4145227997 | 0.0 | 0.0059563687 | 0.4430449612 |
| act(p(MGI:Cxcl12)) | -0.367050433 | 0.0 | 0.0701530797 | 1.0 | -0.0206784639 | 0.285988024 |
| act(p(MGI:Cxcr3)) | -0.1178263212 | 0.0043497268 | -0.1271594049 | 0.0 | -0.0013507226 | 0.4928628495 |
| act(p(MGI:Erbb3)) | -0.1186412565 | 6.64E-8 | -0.0664860463 | 0.0896901408 | -0.0051522688 | 0.445281203 |
| act(p(MGI:Erbb4)) | 0.1581437696 | 0.008122449 | 0.2820576635 | 0.0 | -0.0097468175 | 0.3791968504 |
| act(p(MGI:Ffar3)) | 0.2850052596 | 0.0 | 0.4384305746 | 0.0 | 0.0200511532 | 0.2410422535 |
| act(p(MGI:Fgfr1)) | 0.0751056498 | 0.008122449 | 0.044126746 | 0.7601441441 | 0.0199387906 | 0.1543265306 |
| act(p(MGI:Fmr1)) | 0.0955671775 | 2.584E-7 | 0.0863475469 | 0.5167017544 | 0.010814753 | 0.3555119826 |
| act(p(MGI:Foxo3)) | 0.1226662376 | 2.21E-8 | -0.248502469 | 0.0 | 0.0119341844 | 0.3958486486 |
| act(p(MGI:Gata4)) | -0.2052520466 | 0.0043497268 | -0.0753608559 | 0.0285211726 | 0.0177779329 | 0.1342650602 |
| act(p(MGI:Gba)) | -0.8139914253 | 0.0 | -0.7328820229 | 0.0 | -0.1097533173 | 0.0383614458 |
| act(p(MGI:Gdnf)) | 0.1169784588 | 4.36E-8 | 0.0950524235 | 0.0285211726 | -0.0050725748 | 0.4347788779 |
| act(p(MGI:Gja1)) | -0.2145547264 | 0.0 | 0.1980159876 | 0.0 | -0.0157564372 | 0.3459160494 |
| act(p(MGI:Gjb6)) | 0.1306005122 | 1.4E-8 | 0.0489045897 | 0.2397149425 | 0.0322382078 | 0.1240519481 |
| act(p(MGI:Grk2)) | 0.201956591 | 0.0043497268 | 0.0239804112 | 0.1274616614 | 0.0315151863 | 0.1903478261 |
| act(p(MGI:Hdac3)) | -0.1078298004 | 0.008122449 | 0.0924767464 | 0.0285211726 | 0.0017942547 | 0.4303416252 |
| act(p(MGI:Hdac4)) | -0.1776505528 | 0.0 | -0.114319216 | 0.0 | -0.1197041152 | 0.0 |
| act(p(MGI:Hdac8)) | -0.2641642251 | 0.0 | 0.0335868165 | 0.6849592476 | -0.020421529 | 0.3049464789 |
| act(p(MGI:Ifna2)) | 0.4024624337 | 0.0 | 0.369102785 | 0.0 | 0.022774229 | 0.16 |
| act(p(MGI:Ifnar1)) | 0.3852237972 | 0.0 | 0.3053246099 | 0.0 | 0.0232006182 | 0.1875022222 |
| act(p(MGI:Ifnb1)) | 0.5330945856 | 0.0 | 0.5313180549 | 0.0 | 0.0326286673 | 0.04975 |
| act(p(MGI:Ifng)) | 0.4978557898 | 0.0 | 0.4765593219 | 0.0 | 0.0260321977 | 0.1105555556 |
| act(p(MGI:Ifngr1)) | 0.1565909807 | 4.519E-7 | 0.2851188031 | 0.0122934363 | 0.0166489449 | 0.3330631579 |
| act(p(MGI:Il10ra)) | -0.309685316 | 0.0 | -0.245330095 | 0.0 | -0.0161065717 | 0.2566905537 |
| act(p(MGI:Il19)) | 0.2174289833 | 0.0 | 0.0768952234 | 1.0E-9 | 0.0467640971 | 0.0 |
| act(p(MGI:Il1b)) | 0.3028078578 | 0.0 | 0.2072171242 | 0.0 | 0.0173897103 | 0.3555119826 |
| act(p(MGI:Il23a)) | 0.1855419195 | 0.0 | 0.2130012173 | 0.0 | 0.0156807844 | 0.2636270096 |
| act(p(MGI:Il27)) | 0.2505802816 | 0.0 | 0.4728653669 | 0.0 | 0.0667642711 | 0.0 |
| act(p(MGI:Il6)) | 0.1444909121 | 1.0E-10 | 0.3825453735 | 0.0 | 0.0319078695 | 0.0383614458 |
| act(p(MGI:Insr)) | -0.2587154088 | 0.0 | -0.3730451136 | 0.0 | -0.0260671571 | 0.2061354582 |
| act(p(MGI:Irak4)) | 0.1404088955 | 2.0E-10 | 0.1248966066 | 0.0225017668 | 0.011140889 | 0.3521129412 |
| act(p(MGI:Irf1)) | 0.3555980075 | 0.0 | 0.2731728571 | 0.0 | 0.0270010676 | 0.0796 |
| act(p(MGI:Irf3)) | 0.195483643 | 0.0 | 0.0421974094 | 0.0503493976 | -0.0212133858 | 0.0848196721 |
| act(p(MGI:Irf9)) | 0.1561806888 | 1.0E-10 | 0.0889954916 | 0.0225017668 | 0.0159041104 | 0.2184511278 |
| act(p(MGI:Jak1)) | 0.1298374348 | 3.8E-8 | -0.0387243922 | 0.370306163 | -0.0348761287 | 0.0241212121 |
| act(p(MGI:Jak2)) | 0.1076139727 | 7.14E-8 | -0.0146445356 | 0.6608063492 | 0.0222987337 | 0.1675789474 |
| act(p(MGI:Jun)) | 0.1672978528 | 1.1E-9 | 0.0711701201 | 7.27E-8 | -0.022515235 | 0.1053529412 |
| act(p(MGI:Junb)) | 0.3406038044 | 0.0 | 0.2711335826 | 0.0 | 0.0288111558 | 0.151814433 |
| act(p(MGI:Klf4)) | 0.0865175946 | 0.008122449 | 0.0223264819 | 0.0480966767 | -0.0038420538 | 0.435474026 |
| act(p(MGI:Lcn2)) | 0.1156716733 | 4.8E-9 | 0.0308483186 | 0.0013277059 | 0.0095830842 | 0.4375448718 |
| act(p(MGI:Lif)) | 0.4663647303 | 0.0 | 0.2544632628 | 0.0 | 0.0527869556 | 4.0E-10 |
| act(p(MGI:Mst1)) | -0.2410017938 | 0.0 | 0.0023124048 | 0.7626347305 | 0.0011066412 | 0.4744958217 |
| act(p(MGI:Mtor)) | -0.161125907 | 0.0043497268 | 0.2816709741 | 0.0 | -0.0185932843 | 0.2768695652 |
| act(p(MGI:Myd88)) | 0.1534347947 | 1.0E-10 | 0.2502609461 | 0.0 | 0.0243945137 | 0.1467486034 |
| act(p(MGI:Ncor2)) | 0.0781370089 | 3.1218E-6 | 0.0654562693 | 0.3326320166 | 0.0149827547 | 0.2484161074 |
| act(p(MGI:Nfia)) | 0.0916861104 | 1.8204E-5 | 0.0128197639 | 1.0 | -0.0076391264 | 0.4422222222 |
| act(p(MGI:Nlgn3)) | 0.1208879081 | 3.4E-9 | 0.1526576714 | 0.1842768496 | -0.0170827489 | 0.2432222222 |
| act(p(MGI:Olig2)) | -0.1387359869 | 1.0E-10 | -0.0953496836 | 0.1035447154 | -0.0276238179 | 0.1025151515 |
| act(p(MGI:Osm)) | 0.57802598 | 0.0 | 0.334235115 | 0.0 | 0.0299207823 | 0.1404705882 |
| act(p(MGI:Pln)) | -0.1947698035 | 0.0 | -0.1583252883 | 0.0097469388 | 0.0291420294 | 0.04975 |
| act(p(MGI:Ppara)) | -0.1239468691 | 2.5E-9 | 0.0925852286 | 0.1350913838 | -0.0025498679 | 0.4720335664 |
| act(p(MGI:Ppargc1a)) | -0.2041353673 | 0.0 | 0.2516676067 | 0.0 | 0.008838797 | 0.3521129412 |
| act(p(MGI:Ppargc1b)) | 0.1672612274 | 1.0E-10 | 0.178412434 | 0.1295328084 | 0.0150085938 | 0.30248 |
| act(p(MGI:Pten)) | -0.2496945618 | 0.0 | -0.0760038056 | 0.0268918919 | -0.0596405911 | 0.0 |
| act(p(MGI:Ptgds)) | 0.1933042485 | 0.0 | 0.0679750497 | 0.1654278729 | 0.0349999933 | 0.0452272727 |
| act(p(MGI:Ptger3)) | -0.0595907795 | 4.18473E-4 | -0.2174353402 | 0.0 | -0.0082591486 | 0.3469249395 |
| act(p(MGI:Ptk2)) | 0.1364053456 | 4.0E-10 | 0.1413173269 | 0.0 | 0.0442261082 | 1.8E-8 |
| act(p(MGI:Ptpn11)) | -0.1184087859 | 3.4E-9 | 0.2170035758 | 0.0034759825 | 0.0142246769 | 0.1903478261 |
| act(p(MGI:Rara)) | -0.1075626358 | 6.2E-9 | -0.040441 | 0.2706048565 | -0.0083087017 | 0.4035277778 |
| act(p(MGI:Rarb)) | -0.1315267714 | 1.07E-8 | -0.0140501935 | 0.5797533333 | 0.0067316017 | 0.3965683453 |
| act(p(MGI:Rela)) | 0.1059004536 | 9.3E-9 | 0.2123669207 | 0.9939301075 | -0.0381238325 | 0.0322702703 |
| act(p(MGI:Rhob)) | 0.1295381068 | 0.0043497268 | 0.2703777561 | 0.0 | 0.0329770283 | 0.0562828283 |
| act(p(MGI:Rhoc)) | 0.1721740035 | 0.0 | 0.2071127604 | 0.0 | 0.0362141213 | 0.0452272727 |
| act(p(MGI:Rps6)) | -0.1069108473 | 1.95E-8 | -0.0332812742 | 0.0285211726 | -0.0097011115 | 0.4076401384 |
| act(p(MGI:Rps6kb1)) | -0.2499904213 | 0.0 | -0.0394544792 | 5.41508E-4 | -0.1062758195 | 0.0 |
| act(p(MGI:Rxra)) | 0.1194541622 | 2.3E-9 | 0.0323563324 | 0.4293033708 | -0.0027604987 | 0.4677062147 |
| act(p(MGI:S1pr1)) | 0.2259694064 | 0.0 | 0.214776234 | 0.0067744681 | 0.0372556258 | 0.1183243243 |
| act(p(MGI:Serpine1)) | -0.2495712471 | 0.0043497268 | -0.1310626158 | 1.27E-7 | -0.0045505853 | 0.5084397906 |
| act(p(MGI:Socs1)) | 0.1205592132 | 1.0E-10 | 0.0386520428 | 0.1069892473 | -0.0087815039 | 0.3845338346 |
| act(p(MGI:Sp1)) | 0.143265107 | 2.7E-9 | 0.0050664599 | 0.3949266409 | 0.0155921255 | 0.293872093 |
| act(p(MGI:Stat1)) | 0.2627284636 | 0.0 | 0.1956050949 | 0.0 | 6.130655E-4 | 0.5705840708 |
| act(p(MGI:Tgfb2)) | -0.1623063811 | 0.0043497268 | -0.050175656 | 0.0967513812 | -0.0394236314 | 0.0132666667 |
| act(p(MGI:Tgm1)) | -0.2349680617 | 0.0 | -0.0238184951 | 0.4489499072 | -0.0110322467 | 0.4021748252 |
| act(p(MGI:Tlr2)) | 0.3520307295 | 0.0 | 0.452955297 | 0.0 | 0.0464780633 | 9.9E-9 |
| act(p(MGI:Tlr3)) | 0.3430048974 | 0.0 | 0.1606974484 | 0.0 | 0.0316264886 | 0.089152 |
| act(p(MGI:Tlr4)) | 0.5795791412 | 0.0 | 0.7118210706 | 0.0 | 0.0248678743 | 0.1622912621 |
| act(p(MGI:Tlr5)) | 0.3064784712 | 0.0 | 0.3221684161 | 0.0 | 0.0407794569 | 0.0132666667 |
| act(p(MGI:Tlr7)) | 0.2435509939 | 0.0 | 0.2424157699 | 0.0 | -0.018929344 | 0.1404705882 |
| act(p(MGI:Tlr9)) | 0.3899876101 | 0.0 | 0.3974588735 | 0.0 | 0.0154248368 | 0.3554232558 |
| act(p(MGI:Tnf)) | 0.3486439028 | 0.0 | 0.2283457459 | 0.0 | -2.956417E-4 | 0.4874088184 |
| act(p(MGI:Vhl)) | -0.1457172858 | 2.7E-9 | -0.1530557532 | 0.4280377358 | 0.0011251519 | 0.5076850394 |
| act(p(MGI:Vim)) | -0.2738865371 | 0.0 | -0.1587824545 | 0.0390674847 | -0.0352777232 | 0.0132666667 |
| act(p(SFAM:"DRD Family")) | 0.3360558722 | 0.0 | -0.1036555108 | 1.0 | 0.0170592074 | 0.3676440678 |
| act(p(SFAM:"FGFR Family")) | -0.1019645039 | 1.062E-7 | 0.0833546414 | 0.0390674847 | 0.0051506871 | 0.3676440678 |
| act(p(SFAM:"MAPK p38 Family")) | 0.2014277322 | 0.0 | 0.2720467319 | 0.0 | -0.0116889894 | 0.2866547619 |
| act(p(SFAM:"MEK1/2 Family")) | 0.1110226061 | 0.0043497268 | 0.1805200558 | 0.0 | -0.0121897623 | 0.3521129412 |
| act(p(SFAM:"PRKC Family")) | 0.284493768 | 0.0 | 0.1319752846 | 0.0308129032 | -0.0155616247 | 0.2253382353 |
| act(p(SFAM:"SRC Family")) | 0.2553102513 | 0.0 | 0.2291861158 | 0.0 | 0.0292320377 | 0.1275641026 |
| act(p(SFAM:"TNFRSF Family")) | 0.308732405 | 0.0 | 0.2429101864 | 0.0 | 0.0069453563 | 0.4643333333 |
| complex(GOCC:"AMP-activated protein kinase complex") | -0.0460020509 | 0.0018955725 | -0.4325554897 | 0.0 | 0.0057942964 | 0.5106023468 |
| complex(GOCC:"IkappaB kinase complex") | 0.1842828801 | 0.0 | 0.1258820139 | 0.0 | 0.0391750148 | 3.88E-7 |
| complex(GOCC:"integrin complex") | 0.1686419467 | 2.7E-9 | 0.0285412468 | 0.0165604719 | 0.0257811169 | 0.1981877551 |
| complex(GOCC:"interleukin-1 receptor complex") | 0.280603183 | 0.0 | 0.1517834162 | 0.0 | 0.0289030552 | 0.2278405797 |
| complex(GOCC:"NF-kappaB complex") | 0.4044836588 | 0.0 | 0.4120238081 | 0.0 | 0.0327789045 | 0.1719061033 |

**Supplementary Table 2. Significant iNodes in astrocytes isolated from a transgenic mouse model of the early stage (7m) or late-stage (13m) of Alzheimer`s disease compared to age-matched non-transgenic controls and scored with the reactive astrocyte CBN.**

| iNode name | AD 7m fold-change | AD 7m adjusted p value | AD 13m fold-change | AD 13m adjusted p value |
| --- | --- | --- | --- | --- |
| a(CHEBI:ATP) | 0.0300325556 | 0.4869240781 | -0.1226121964 | 7.48E-8 |
| a(CHEBI:dexamethasone) | -0.0240422084 | 0.0927378641 | -0.064683717 | 7.03606E-5 |
| a(CHEBI:kynurenine) | -0.0280383082 | 0.6976474465 | 0.1931639123 | 2.0E-9 |
| act(p(MGI:Ache)) | -0.0054879129 | 0.7482902208 | -0.06013204 | 2.847049E-4 |
| act(p(MGI:Akt2)) | 0.1064950595 | 0.0 | -0.0229073897 | 1.0 |
| act(p(MGI:Arntl)) | -0.0499294385 | 2.88016E-5 | 0.0360221723 | 0.9880175439 |
| act(p(MGI:Bcl2)) | -0.0145323165 | 0.1259484536 | -0.072114272 | 0.0052368421 |
| act(p(MGI:Casp1)) | -0.007456599 | 0.7496918239 | 0.0902063886 | 0.0052368421 |
| act(p(MGI:Casp7)) | -0.0503501294 | 7.17274E-5 | -0.0694855602 | 1.088068E-4 |
| act(p(MGI:Cd40)) | 0.046898158 | 5.57442E-5 | 0.0934091508 | 8.467E-7 |
| act(p(MGI:Cdh2)) | -0.0200167659 | 0.813329463 | 0.1404291669 | 6.7E-8 |
| act(p(MGI:Chrna7)) | -0.0773706259 | 1.2E-8 | 0.2437924072 | 0.0 |
| act(p(MGI:Drd2)) | -0.0037800066 | 0.8613184979 | 0.0810825249 | 7.7191E-6 |
| act(p(MGI:Egr1)) | -0.0508664828 | 0.2744827586 | 0.191871019 | 0.0 |
| act(p(MGI:Erbb3)) | -0.0253482985 | 0.0976226415 | 0.0654203182 | 4.910763E-4 |
| act(p(MGI:Erbb4)) | 0.0744427793 | 6.89E-8 | -0.0213888237 | 1.0 |
| act(p(MGI:Foxo1)) | 0.0380783079 | 0.3877948718 | -0.1484246549 | 3.0E-10 |
| act(p(MGI:Gba)) | -0.1652615141 | 8.99E-8 | -0.0719168146 | 0.0402463652 |
| act(p(MGI:Gfap)) | -0.015962604 | 0.0782144788 | 0.065386591 | 9.20081E-5 |
| act(p(MGI:Gja1)) | -0.0110554972 | 0.2237000859 | -0.2512255041 | 0.0 |
| act(p(MGI:Gper1)) | -0.050171767 | 2.04802E-5 | 0.1472585706 | 7.0E-10 |
| act(p(MGI:Hbegf)) | 0.0157669688 | 0.1049703208 | 0.0948030911 | 7.25E-7 |
| act(p(MGI:Hdac1)) | -0.017867842 | 0.2595018315 | -0.0441712897 | 0.0097073171 |
| act(p(MGI:Hdac3)) | 0.0192440503 | 0.384630491 | -0.1186809849 | 2.22E-8 |
| act(p(MGI:Hdac4)) | 0.0468053095 | 5.70602E-5 | 0.0178039828 | 0.1357678688 |
| act(p(MGI:Hdac8)) | 0.0195608704 | 0.6314081996 | -0.1541821637 | 2.0E-10 |
| act(p(MGI:Icam1)) | 0.0370938087 | 0.9606896552 | -0.1547673526 | 0.0 |
| act(p(MGI:Ifng)) | 0.062004163 | 9.73E-8 | 0.0048394751 | 0.3897359695 |
| act(p(MGI:Il10)) | 0.0266382357 | 0.4068444444 | -0.1138644885 | 6.37E-8 |
| act(p(MGI:Il10ra)) | -0.0033458892 | 0.3967932317 | -0.0968463437 | 0.0052368421 |
| act(p(MGI:Il19)) | 0.006447804 | 0.2988315068 | 0.0891217841 | 0.0097073171 |
| act(p(MGI:Il6)) | 0.0662980703 | 0.0388292683 | -0.1656774344 | 1.0E-10 |
| act(p(MGI:Ilk)) | -0.0802578555 | 0.0 | 0.0583020784 | 1.0 |
| act(p(MGI:Irf3)) | -0.0255675468 | 0.1721081081 | 0.1004229609 | 5.76E-8 |
| act(p(MGI:Irs1)) | 0.0241141064 | 0.6213747715 | -0.1192757611 | 5.07E-8 |
| act(p(MGI:Itgb1)) | -0.0353638155 | 0.1229117647 | 0.1158233228 | 7.57E-8 |
| act(p(MGI:Jak1)) | 0.0344166795 | 0.0017363167 | 0.1034078926 | 2.033E-7 |
| act(p(MGI:Lama2)) | -0.007985797 | 0.4624959377 | -0.3546416864 | 3.32522E-5 |
| act(p(MGI:Lcn2)) | -0.0202163252 | 0.7995535714 | 0.1073120781 | 0.0052368421 |
| act(p(MGI:Lef1)) | -0.0621909983 | 0.0230724638 | 0.2120015516 | 0.0 |
| act(p(MGI:Map3k7)) | -0.0042358353 | 0.7690584615 | 0.1211660476 | 1.28E-8 |
| act(p(MGI:Mapk14)) | -0.0075565719 | 0.3542760563 | 0.0386499578 | 0.0097073171 |
| act(p(MGI:Mapk8)) | -0.0184596933 | 0.555608 | 0.1070988202 | 1.252E-7 |
| act(p(MGI:Mmp3)) | 0.0905388272 | 0.0 | -0.2980810037 | 0.0 |
| act(p(MGI:Mmp7)) | 0.042626385 | 2.680291E-4 | -0.0232840588 | 0.0910155534 |
| act(p(MGI:Mst1)) | -0.013511198 | 0.1331634412 | -0.0796913968 | 0.0052368421 |
| act(p(MGI:Mtor)) | 0.0656358336 | 1.803E-7 | -0.1996404531 | 0.0 |
| act(p(MGI:Nfe2l2)) | -0.0183123155 | 0.0594922946 | -0.0668318263 | 4.84209E-5 |
| act(p(MGI:Nr3c1)) | 0.0510536909 | 0.1388993289 | -0.172254771 | 3.0E-10 |
| act(p(MGI:Nrf1)) | -0.0938874803 | 0.0322702703 | 0.3125027132 | 0.0 |
| act(p(MGI:Olig2)) | 0.0175957542 | 0.0618034096 | 0.0802176857 | 0.0052368421 |
| act(p(MGI:Osm)) | 0.0268576935 | 0.2068898678 | 0.0450540992 | 0.0015747622 |
| act(p(MGI:Per2)) | -0.0062131303 | 0.7838473282 | 0.0743206394 | 0.0052368421 |
| act(p(MGI:Pik3ca)) | -0.0198349766 | 0.547854251 | 0.0949879542 | 0.0097073171 |
| act(p(MGI:Pink1)) | 0.0096194389 | 0.7468797468 | -0.1196095131 | 7.62E-8 |
| act(p(MGI:Pparg)) | 0.0142981755 | 0.182755102 | -0.0878144737 | 1.8082E-6 |
| act(p(MGI:Ppargc1a)) | 0.0273571016 | 0.6235812274 | -0.2251499867 | 0.0 |
| act(p(MGI:Ptger3)) | -0.061292153 | 1.1062E-6 | 0.0993183146 | 0.4340220751 |
| act(p(MGI:Ptpn11)) | 0.0375513015 | 0.8458917379 | -0.1777673172 | 0.0 |
| act(p(MGI:Rac1)) | 0.0598315009 | 0.4875930736 | -0.2484952115 | 0.0 |
| act(p(MGI:Rara)) | -0.0163836095 | 0.1682971429 | -0.0389218365 | 0.0031543163 |
| act(p(MGI:Rarg)) | -0.0398709172 | 2.511629E-4 | 0.0810238509 | 8.0269E-6 |
| act(p(MGI:Rhoa)) | -0.0062425522 | 0.3132599039 | 0.0539489206 | 9.586855E-4 |
| act(p(MGI:Slc16a1)) | -0.0126767224 | 0.5754031008 | 0.0637533979 | 0.0097073171 |
| act(p(MGI:Slc1a1)) | -0.0461372208 | 0.1640242424 | 0.1473942748 | 9.4E-8 |
| act(p(MGI:Stat1)) | 0.0123481453 | 0.4994175589 | 0.0450562166 | 0.0053090083 |
| act(p(MGI:Tgm1)) | -0.0034364698 | 0.5143644068 | -0.0857037939 | 2.03672E-5 |
| act(p(MGI:Tlr2)) | 0.0301633591 | 0.2017013575 | 0.0466631369 | 0.0010382161 |
| act(p(MGI:Tlr3)) | -0.0409744345 | 0.2008256881 | 0.1403961658 | 3.9E-9 |
| act(p(MGI:Tnf)) | 0.0124590995 | 0.4527706422 | 0.0891552891 | 7.704E-7 |
| act(p(MGI:Trp53)) | -0.0310586148 | 0.86565 | 0.1685367638 | 0.0 |
| act(p(MGI:Tyk2)) | -0.0218485692 | 0.2575294118 | 0.0814864181 | 0.0097073171 |
| act(p(MGI:Vim)) | -0.0258576558 | 0.9011934605 | -0.0624782258 | 5.96711E-5 |
| act(p(SFAM:"FGFR Family")) | -0.0214181056 | 0.0326783493 | -0.0750944911 | 0.0052368421 |
| act(p(SFAM:"NOTCH Family")) | 0.0786549143 | 0.5786307692 | -0.267974611 | 0.0 |
| act(p(SFAM:"PRKC Family")) | 0.0077091544 | 0.8523739377 | 0.1020475599 | 1.756E-7 |
| act(p(SFAM:"RAS Family")) | -0.0193630573 | 0.5137664544 | 0.0805763367 | 1.87975E-5 |
| complex(GOCC:"AMP-activated protein kinase complex") | -0.1060889626 | 0.0 | 0.2862915197 | 0.0 |
| complex(GOCC:"proteasome complex") | 0.0394638703 | 0.0530666667 | -0.120854544 | 1.012E-7 |

**Supplementary Table 3. Significant iNodes in astrocytes isolated from humans with multiple sclerosis (MS) compared to control subjects scored with the reactive astrocyte CBN.**

| iNode name | MS fold-change | MS adjusted p value |
| --- | --- | --- |
| a(CHEBI:"calcium(2+)") | 0.0520121435 | 0.0038921569 |
| a(CHEBI:"nitric oxide") | 0.1152124715 | 7.57E-8 |
| a(CHEBI:ascorbate) | -0.0910050949 | 5.48E-8 |
| a(CHEBI:ATP) | -0.0955339431 | 1.86E-9 |
| a(CHEBI:dexamethasone) | -0.0751674325 | 2.24E-7 |
| a(CHEBI:dopamine) | -0.0682859166 | 3.01E-8 |
| a(CHEBI:kynurenine) | 0.0878582611 | 1.26E-8 |
| a(CHEBI:lactate) | 0.1596041685 | 6.97E-13 |
| act(p(HGNC:ADRB2)) | -0.0824320173 | 6.13E-7 |
| act(p(HGNC:AGER)) | -0.0610488871 | 8.02E-6 |
| act(p(HGNC:AGTR2)) | 0.0510713007 | 0.0038921569 |
| act(p(HGNC:AKT2)) | 0.1766864152 | 2.47E-12 |
| act(p(HGNC:AKT3)) | 0.190186797 | 3.28E-10 |
| act(p(HGNC:APC)) | 0.0428640469 | 6.47677E-4 |
| act(p(HGNC:ARC)) | 0.0785351954 | 2.24E-7 |
| act(p(HGNC:ARNTL)) | -0.0766092415 | 1.56E-6 |
| act(p(HGNC:ARRB2)) | -0.0668526835 | 0.0073860465 |
| act(p(HGNC:BCL2)) | -0.0687947452 | 5.54E-7 |
| act(p(HGNC:BDNF)) | -0.0793809784 | 6.9E-7 |
| act(p(HGNC:CASP7)) | -0.1020997335 | 3.77E-10 |
| act(p(HGNC:CASP8)) | 0.0743998694 | 2.11E-7 |
| act(p(HGNC:CAT)) | -0.0527146726 | 3.671755E-4 |
| act(p(HGNC:CD109)) | 0.065564779 | 4.35E-6 |
| act(p(HGNC:CD40)) | 0.115650013 | 3.79E-11 |
| act(p(HGNC:CD44)) | 0.1071709358 | 2.53E-6 |
| act(p(HGNC:CDH2)) | 0.1753741238 | 2.83E-17 |
| act(p(HGNC:CHRNA7)) | 0.146510058 | 2.87E-13 |
| act(p(HGNC:DRD2)) | 0.1388081252 | 7.65E-11 |
| act(p(HGNC:EGFR)) | -0.0601317171 | 4.6E-6 |
| act(p(HGNC:EGR1)) | 0.1535021111 | 3.4E-16 |
| act(p(HGNC:ELK1)) | -0.0716928507 | 1.53E-6 |
| act(p(HGNC:EPAS1)) | -0.0557713636 | 0.0038921569 |
| act(p(HGNC:FFAR3)) | 0.1158680869 | 1.76E-9 |
| act(p(HGNC:FMR1)) | 0.0771959768 | 3.4E-5 |
| act(p(HGNC:FOXA2)) | -0.0817894552 | 2.16E-8 |
| act(p(HGNC:FOXO1)) | -0.1186971651 | 3.24E-10 |
| act(p(HGNC:GBA)) | -0.178861814 | 8.81E-12 |
| act(p(HGNC:GJB6)) | 0.0711999564 | 1.01E-5 |
| act(p(HGNC:GPER1)) | 0.0749220683 | 1.09E-6 |
| act(p(HGNC:GRK2)) | 0.0725460111 | 1.26E-6 |
| act(p(HGNC:GSK3B)) | 0.0497737636 | 1.195454E-4 |
| act(p(HGNC:HBEGF)) | 0.1193674238 | 8.11E-11 |
| act(p(HGNC:HDAC1)) | -0.0654423532 | 2.53E-6 |
| act(p(HGNC:HDAC3)) | -0.1056462004 | 1.28E-8 |
| act(p(HGNC:HDAC4)) | -0.097173677 | 1.98E-5 |
| act(p(HGNC:HDAC8)) | -0.149206654 | 6.31E-15 |
| act(p(HGNC:HES1)) | -0.0925147691 | 3.18E-7 |
| act(p(HGNC:HIF1A)) | 0.0568920349 | 0.0038921569 |
| act(p(HGNC:ICAM1)) | 0.1483110312 | 2.42E-11 |
| act(p(HGNC:IFNAR1)) | 0.0800925796 | 1.06E-7 |
| act(p(HGNC:IL10)) | -0.093639643 | 9.13E-9 |
| act(p(HGNC:IL18)) | -0.0536563382 | 0.0038921569 |
| act(p(HGNC:IL23A)) | 0.058767722 | 1.99E-5 |
| act(p(HGNC:ILK)) | -0.0713865353 | 2.47E-6 |
| act(p(HGNC:IRF3)) | 0.1047126122 | 1.47E-9 |
| act(p(HGNC:IRS1)) | -0.0444953585 | 3.573972E-4 |
| act(p(HGNC:ITGA11)) | 0.151466814 | 1.94E-6 |
| act(p(HGNC:ITGB1)) | 0.0601825923 | 0.0073860465 |
| act(p(HGNC:LCN2)) | 0.0678956586 | 2.47E-6 |
| act(p(HGNC:LEF1)) | 0.1614633683 | 2.02E-13 |
| act(p(HGNC:LIF)) | 0.0658580036 | 1.03E-5 |
| act(p(HGNC:MAPK14)) | 0.1013679421 | 1.35E-9 |
| act(p(HGNC:MAPK9)) | 0.058711148 | 8.77E-5 |
| act(p(HGNC:MMP10)) | -0.0601929433 | 0.0073860465 |
| act(p(HGNC:MMP3)) | -0.1211943146 | 3.71E-11 |
| act(p(HGNC:NFE2L2)) | -0.0556649843 | 0.0038921569 |
| act(p(HGNC:NFIA)) | 0.0433945292 | 0.0073860465 |
| act(p(HGNC:NLRP3)) | 0.088656475 | 4.4E-8 |
| act(p(HGNC:NR3C1)) | -0.1198251909 | 8.16E-14 |
| act(p(HGNC:OLIG2)) | -0.0919465024 | 1.12E-6 |
| act(p(HGNC:PIK3CA)) | 0.0858068661 | 3.02E-8 |
| act(p(HGNC:PINK1)) | -0.1056573453 | 4.46E-9 |
| act(p(HGNC:PPARGC1B)) | 0.0927266819 | 1.91E-9 |
| act(p(HGNC:PTEN)) | -0.1671519457 | 6.38E-11 |
| act(p(HGNC:PTGS2)) | 0.1057296954 | 5.06E-11 |
| act(p(HGNC:PTPN11)) | -0.0798867124 | 7.57E-8 |
| act(p(HGNC:RARG)) | 0.0664060899 | 3.99E-7 |
| act(p(HGNC:RELA)) | 0.0922050777 | 6.35E-9 |
| act(p(HGNC:ROR2)) | -0.1093078124 | 1.94E-10 |
| act(p(HGNC:RPS6)) | 0.0602296865 | 0.0038921569 |
| act(p(HGNC:S100B)) | -0.0435611752 | 0.0038921569 |
| act(p(HGNC:SLC1A1)) | 0.0709457763 | 2.69E-7 |
| act(p(HGNC:SOCS1)) | 0.0635029043 | 1.02E-5 |
| act(p(HGNC:SOD2)) | 0.0358695151 | 0.0038921569 |
| act(p(HGNC:TGM1)) | -0.0967008644 | 9.21E-9 |
| act(p(HGNC:THBS1)) | -0.0951090202 | 5.64E-9 |
| act(p(HGNC:TLR2)) | 0.1002338885 | 6.09E-8 |
| act(p(HGNC:TLR3)) | 0.076746212 | 0.0038921569 |
| act(p(HGNC:TNF)) | 0.1313416202 | 6.24E-11 |
| act(p(HGNC:TP53)) | 0.113870097 | 2.04E-12 |
| act(p(HGNC:TRAF3IP2)) | 0.1360397643 | 4.94E-6 |
| act(p(PMIPFAM:"Cannabinoid Receptor Family")) | -0.0659926396 | 3.89E-8 |
| act(p(PMIPFAM:"ITPR Family")) | 0.0639024926 | 4.13E-6 |
| act(p(SFAM:"MAPK JNK Family")) | 0.0474545289 | 4.45E-5 |
| act(p(SFAM:"NOTCH Family")) | -0.0544101826 | 1.35E-5 |
| act(p(SFAM:"PRKC Family")) | 0.124242837 | 1.39E-11 |
| act(p(SFAM:"TNFRSF Family")) | 0.0957093131 | 5.36E-8 |
| complex(GOCC:"AMP-activated protein kinase complex") | 0.0698117165 | 4.37E-6 |
| complex(GOCC:"IkappaB kinase complex") | 0.0840903385 | 2.43E-8 |

**Supplementary Table 4**. **Significant iNodes in astrocytes isolated from the early stage (3m) or late-stage (4m) of amyotrophic lateral sclerosis (ALS) in transgenic mice compared to age-matched non-transgenic controls and scored with the reactive astrocyte CBN.**

| iNode name | ALS 3m fold-change | ALS 3m adjusted p value | ALS 4m fold-change | ALS 4m adjusted p value |
| --- | --- | --- | --- | --- |
| a(CHEBI:"beta-amyloid") | 0.0906251554 | 1.0E-10 | 0.2063041614 | 0.0 |
| a(CHEBI:"calcium(2+)") | 0.0504264772 | 0.0182290076 | 0.1567548434 | 0.0 |
| a(CHEBI:"D-glucose") | 0.0320469269 | 0.0582439024 | 0.0964259657 | 0.0 |
| a(CHEBI:"hydrogen peroxide") | 0.0187304645 | 0.0865217391 | 0.1321281623 | 0.0 |
| a(CHEBI:"NAD(+)") | 0.0255878219 | 0.1495962877 | 0.0912292482 | 0.0072804878 |
| a(CHEBI:"nitric oxide") | 0.086215602 | 0.0 | 0.421247028 | 0.0 |
| a(CHEBI:"N-methyl-4-phenylpyridinium") | 0.3643766879 | 0.0 | 0.6595641574 | 0.0 |
| a(CHEBI:"valproic acid") | -0.0412239427 | 0.0255857143 | -0.2547894181 | 0.0 |
| a(CHEBI:ascorbate) | -0.057058547 | 6.76393E-5 | -0.2626381261 | 0.0 |
| a(CHEBI:ATP) | -0.0684656085 | 8.0E-9 | -0.2054084335 | 0.0 |
| a(CHEBI:dexamethasone) | -0.1166397331 | 0.0 | -0.3046812865 | 0.0 |
| a(CHEBI:dopamine) | -0.070586301 | 7.0E-10 | -0.0998863303 | 0.0 |
| a(CHEBI:kynurenine) | 0.0054417361 | 0.363569356 | 0.1275395316 | 0.0072804878 |
| a(CHEBI:lactate) | 0.0552543723 | 2.2688E-6 | 0.2082941114 | 0.0 |
| act(p(MGI:Ace2)) | 0.0741530527 | 0.0037725118 | 0.127732386 | 0.0 |
| act(p(MGI:Ache)) | -0.1122272423 | 0.0 | -0.3168352107 | 0.0 |
| act(p(MGI:Ager)) | -0.0617981187 | 3.241E-7 | -0.0155459147 | 0.1237874016 |
| act(p(MGI:Agtr2)) | 0.0099931509 | 0.3292425249 | 0.1136209075 | 1.0E-10 |
| act(p(MGI:Ahr)) | -0.003363291 | 0.3924393395 | -0.1210094157 | 0.0 |
| act(p(MGI:Akt1)) | -0.1051249347 | 0.0 | -0.0804692841 | 1.8E-9 |
| act(p(MGI:Akt2)) | 0.3789265086 | 0.0 | 0.6737458244 | 0.0 |
| act(p(MGI:Akt3)) | 0.2853487626 | 0.0 | 0.6175037146 | 0.0 |
| act(p(MGI:Aldh1a1)) | -0.0197171066 | 0.1460894118 | 0.173286431 | 0.0094761905 |
| act(p(MGI:Apc)) | 0.0595959639 | 1.9E-9 | -0.0498235607 | 0.6295925433 |
| act(p(MGI:Apoe)) | 0.0373680756 | 0.0711123919 | 0.2594265157 | 0.0 |
| act(p(MGI:Arc)) | 0.0388850117 | 0.2047325103 | 0.1924769161 | 0.0026891892 |
| act(p(MGI:Arrb2)) | -0.130781629 | 0.0 | -0.1022654704 | 0.0 |
| act(p(MGI:Atf4)) | 0.0330008823 | 0.3107671233 | 0.1332406338 | 0.0 |
| act(p(MGI:B2m)) | -0.0093254883 | 0.3282337229 | -0.1035169489 | 0.0026891892 |
| act(p(MGI:Bcl2)) | -0.0466071486 | 0.0037725118 | -0.1543529111 | 0.0 |
| act(p(MGI:Bdkrb2)) | 0.0246191514 | 0.0748376068 | 0.0695357378 | 0.0050700637 |
| act(p(MGI:Bdnf)) | -0.0716536822 | 1.9E-9 | -0.1325451107 | 0.0 |
| act(p(MGI:Casp1)) | 0.0773345383 | 2.0E-9 | 0.1880353599 | 0.0 |
| act(p(MGI:Casp7)) | -0.2404479337 | 0.0 | -0.4644775292 | 0.0 |
| act(p(MGI:Casp8)) | 0.016424381 | 0.1624125561 | 0.1119954818 | 0.0026891892 |
| act(p(MGI:Ccr2)) | 0.0936825746 | 5.0E-10 | 0.0865671845 | 0.1644870849 |
| act(p(MGI:Cd109)) | 0.1188705359 | 0.0 | 0.1668788834 | 0.0 |
| act(p(MGI:Cd40)) | 0.2102426494 | 0.0 | 0.4667403397 | 0.0 |
| act(p(MGI:Cd40lg)) | 0.03462602 | 0.0037725118 | 0.2603375555 | 0.0 |
| act(p(MGI:Cd44)) | 0.0560796624 | 0.0068917749 | 0.2710421613 | 0.0 |
| act(p(MGI:Cdc42)) | -0.1818364269 | 0.0068917749 | -0.2076400817 | 0.0773650108 |
| act(p(MGI:Cdh2)) | 0.0674583515 | 4.713E-7 | 0.3361090133 | 0.0 |
| act(p(MGI:Chrna7)) | 0.0954487722 | 0.0 | 0.3579069411 | 0.0 |
| act(p(MGI:Cntf)) | 0.4383595332 | 0.0 | 0.6516067866 | 0.0135681818 |
| act(p(MGI:Ctnnb1)) | -0.0693690909 | 3.0E-9 | -0.2062562768 | 0.0 |
| act(p(MGI:Cxcl1)) | -0.0454307342 | 0.0068917749 | -0.1713827214 | 0.0 |
| act(p(MGI:Cxcr3)) | -0.048584965 | 0.0129959184 | -0.1162635271 | 0.0026891892 |
| act(p(MGI:Drd2)) | 0.126213338 | 0.0 | 0.263686988 | 0.0 |
| act(p(MGI:Egr1)) | 0.0596290391 | 2.242E-7 | 0.3880372234 | 0.0 |
| act(p(MGI:Eif2ak3)) | 0.0336059205 | 0.0511897106 | 0.0787576329 | 0.0050700637 |
| act(p(MGI:Elk1)) | -0.0480607169 | 0.0942631579 | -0.1648752048 | 0.0 |
| act(p(MGI:Erbb4)) | 0.1166200143 | 0.0 | 0.1716223073 | 0.0 |
| act(p(MGI:Etv5)) | 0.0503926516 | 0.0848196721 | 0.1712773564 | 0.0072804878 |
| act(p(MGI:Ffar3)) | 0.1953961197 | 0.0 | 0.379492351 | 0.0 |
| act(p(MGI:Fgfr1)) | -0.0499060993 | 4.7861E-6 | -0.1538128519 | 0.0 |
| act(p(MGI:Fmr1)) | 0.0441074042 | 4.08768E-5 | 0.0139237003 | 0.1152253885 |
| act(p(MGI:Fn1)) | -0.0524711494 | 0.0282269504 | -0.1406941453 | 0.0050700637 |
| act(p(MGI:Foxa1)) | -0.0395337819 | 0.0068917749 | -0.1706109966 | 0.0 |
| act(p(MGI:Foxa2)) | -0.1193107391 | 0.0 | -0.2672746734 | 0.0 |
| act(p(MGI:Foxo1)) | -0.0812316014 | 1.0E-10 | -0.3413996524 | 0.0 |
| act(p(MGI:Fyn)) | -0.0694739633 | 0.0129959184 | -0.2148314914 | 0.0 |
| act(p(MGI:Gba)) | -0.5081816547 | 0.0 | -0.886917541 | 0.0 |
| act(p(MGI:Gjb6)) | 0.0679725649 | 1.93679E-5 | 0.1670643755 | 1.37202E-5 |
| act(p(MGI:Gper1)) | 0.0681732549 | 1.156E-7 | 0.2594031 | 0.0 |
| act(p(MGI:Gpnmb)) | -0.0816353242 | 1.0E-10 | -0.1678385124 | 0.0 |
| act(p(MGI:Grk2)) | 0.0481153596 | 0.0565061728 | 0.1899656204 | 0.0 |
| act(p(MGI:Hbegf)) | 0.1343741425 | 0.0 | 0.262968979 | 0.0 |
| act(p(MGI:Hdac3)) | -0.0554792195 | 8.724E-7 | -0.2004812086 | 0.0 |
| act(p(MGI:Hdac4)) | -0.0575610774 | 2.8582E-6 | -0.0875641313 | 1.0E-10 |
| act(p(MGI:Hdac8)) | -0.0635705054 | 5.38E-8 | -0.2889943684 | 0.0 |
| act(p(MGI:Hes1)) | -0.0241370171 | 0.2965490196 | -0.1199243733 | 0.0026891892 |
| act(p(MGI:Icam1)) | 0.1316183009 | 0.0 | 0.2889797127 | 0.0 |
| act(p(MGI:Ifna2)) | 0.0328067443 | 0.0621501502 | 0.1158942255 | 0.0 |
| act(p(MGI:Ifnar1)) | 0.1091403687 | 0.0 | 0.1901605002 | 0.0 |
| act(p(MGI:Ifnb1)) | 0.127976379 | 0.0 | 0.2176414522 | 0.0 |
| act(p(MGI:Ifng)) | 0.0823847944 | 0.0 | 0.1794367832 | 0.0 |
| act(p(MGI:Ifngr1)) | 0.1193922369 | 0.0 | 0.0927549494 | 0.0050700637 |
| act(p(MGI:Igf1)) | -0.1417938913 | 0.0 | -0.2395779128 | 0.0 |
| act(p(MGI:Il10ra)) | -0.081982681 | 2.4E-9 | -0.1610317803 | 0.0 |
| act(p(MGI:Il19)) | 0.0689689828 | 1.4E-8 | 0.1472608305 | 0.0 |
| act(p(MGI:Il1b)) | 0.0818651676 | 0.0 | 0.2087271969 | 0.0 |
| act(p(MGI:Il23a)) | 0.1171531755 | 0.0 | 0.3111951472 | 0.0 |
| act(p(MGI:Il27)) | 0.0598249742 | 0.1128506329 | 0.1142911382 | 0.0 |
| act(p(MGI:Ilk)) | -0.1255630252 | 0.0 | -0.1472433175 | 0.0 |
| act(p(MGI:Insr)) | -0.2366622499 | 0.0 | -0.3577453505 | 0.0 |
| act(p(MGI:Irak1)) | -0.0049207162 | 0.5638789546 | -0.1138512308 | 0.0050700637 |
| act(p(MGI:Irf1)) | 0.0695762789 | 0.0037725118 | 0.0789142259 | 0.0026891892 |
| act(p(MGI:Irf3)) | 0.0738568296 | 2.19E-7 | 0.2769344989 | 0.0 |
| act(p(MGI:Irs1)) | -0.017344297 | 0.0682877654 | -0.1381044212 | 0.0 |
| act(p(MGI:Itga11)) | 0.0508759494 | 0.0037725118 | 0.0892391819 | 0.0026891892 |
| act(p(MGI:Itpr2)) | 0.0198605746 | 0.3000849558 | 0.1107957842 | 0.0 |
| act(p(MGI:Jak1)) | -0.0227246147 | 0.2673134328 | -0.0911545529 | 0.0050700637 |
| act(p(MGI:Junb)) | 0.0584668683 | 0.0255857143 | 0.1602584504 | 0.0 |
| act(p(MGI:Lama2)) | -0.1880676075 | 0.0182290076 | -0.6561162369 | 0.0 |
| act(p(MGI:Lcn2)) | 0.0303584206 | 0.0182290076 | 0.1097275311 | 0.0050700637 |
| act(p(MGI:Lef1)) | 0.0749494565 | 5.0E-9 | 0.4818267256 | 0.0 |
| act(p(MGI:Lif)) | 0.1026801711 | 0.0 | 0.2507879842 | 0.0 |
| act(p(MGI:Mafg)) | 0.2535255856 | 0.0068917749 | 0.3744113166 | 0.0646862302 |
| act(p(MGI:Map2k1)) | 0.0694549621 | 0.0037725118 | 0.1488322338 | 0.0 |
| act(p(MGI:Map3k7)) | 0.0067172483 | 0.480987234 | 0.1227435087 | 0.0 |
| act(p(MGI:Mapk14)) | 0.0816162821 | 5.0E-10 | 0.1971400201 | 0.0 |
| act(p(MGI:Mapk8)) | 0.0432851186 | 0.0068917749 | 0.1358302313 | 0.0 |
| act(p(MGI:Mapk9)) | -0.0024980838 | 0.7548630491 | 0.0845259627 | 0.0072804878 |
| act(p(MGI:Mef2c)) | 0.0184744974 | 0.0605996125 | 0.1326723479 | 0.0 |
| act(p(MGI:Mmp10)) | -0.0528788752 | 4.8322E-6 | -0.2066762071 | 0.0 |
| act(p(MGI:Mmp3)) | -0.045517182 | 9.52146E-5 | -0.3864048003 | 0.0 |
| act(p(MGI:Mst1)) | -0.032071936 | 0.1460894118 | -0.1754445697 | 0.0 |
| act(p(MGI:Myd88)) | 0.1145288337 | 0.0 | 0.1642717407 | 0.0 |
| act(p(MGI:Nes)) | 0.0295417264 | 0.0511897106 | 0.1042371817 | 0.0 |
| act(p(MGI:Nfia)) | 0.0525376155 | 0.0068917749 | 0.0810966065 | 3.0E-10 |
| act(p(MGI:Ngfr)) | 0.0324742964 | 0.0500794702 | 0.0850560603 | 2.3E-9 |
| act(p(MGI:Nlrp3)) | 0.1426330758 | 0.0 | 0.251638644 | 0.0 |
| act(p(MGI:Nr1d1)) | -0.0763541111 | 0.0068917749 | -0.187424031 | 0.0026891892 |
| act(p(MGI:Nr3c1)) | -0.0842512264 | 0.0 | -0.3106128185 | 0.0 |
| act(p(MGI:Nr4a2)) | 0.0316609179 | 0.0068917749 | 0.0602964883 | 0.1440380952 |
| act(p(MGI:Olig2)) | -0.0913621016 | 0.0 | -0.3022734842 | 0.0 |
| act(p(MGI:Osm)) | 0.1130131431 | 0.0 | 0.2594601141 | 0.0 |
| act(p(MGI:Osmr)) | 0.0592795387 | 3.476E-7 | 0.0716034132 | 0.1111821862 |
| act(p(MGI:P2rx7)) | -0.0071347281 | 0.6076417112 | 0.0497479638 | 0.0094761905 |
| act(p(MGI:P2ry2)) | -0.0181831972 | 0.2687792208 | -0.2336339349 | 0.0 |
| act(p(MGI:Per2)) | 0.0149063643 | 0.1412565947 | 0.0812730023 | 0.0026891892 |
| act(p(MGI:Pik3ca)) | 0.0755106138 | 1.0E-10 | 0.2808911197 | 0.0 |
| act(p(MGI:Pink1)) | -0.0229671526 | 0.1406973366 | -0.131271686 | 0.0 |
| act(p(MGI:Plat)) | -0.0012411975 | 0.647664042 | -0.1075937908 | 0.0026891892 |
| act(p(MGI:Plin2)) | 0.0905679235 | 0.0 | 0.1657788005 | 0.0 |
| act(p(MGI:Ppard)) | -0.0136333016 | 0.2530635838 | -0.1311128725 | 0.0026891892 |
| act(p(MGI:Pparg)) | -0.0548424989 | 0.0068917749 | -0.1731178594 | 0.0 |
| act(p(MGI:Ppargc1a)) | -0.0412162417 | 6.76393E-5 | -0.232752973 | 0.0 |
| act(p(MGI:Ppargc1b)) | 0.2183435553 | 0.0 | 0.3056247581 | 0.0 |
| act(p(MGI:Pten)) | -0.1020348653 | 0.0 | -0.3850993675 | 0.0 |
| act(p(MGI:Ptger3)) | -0.0512288629 | 0.0068917749 | -0.0613577475 | 7.708E-7 |
| act(p(MGI:Ptger4)) | -0.1676928075 | 0.0354383562 | -0.5565301957 | 8.0E-10 |
| act(p(MGI:Ptgs2)) | 0.0600009348 | 2.78E-8 | 0.1874988615 | 0.0 |
| act(p(MGI:Ptk2)) | 0.0448231341 | 0.0068917749 | 0.0202006284 | 0.2741630616 |
| act(p(MGI:Ptpn11)) | -0.0343581846 | 0.0015981997 | -0.1365042075 | 0.0026891892 |
| act(p(MGI:Rara)) | -0.0243849682 | 0.1099335038 | -0.0778278709 | 0.0050700637 |
| act(p(MGI:Rarg)) | 0.0486099147 | 0.0037725118 | 0.1452751653 | 0.0 |
| act(p(MGI:Rela)) | 0.0796702748 | 0.0232408759 | 0.184567421 | 0.0 |
| act(p(MGI:Rock1)) | -0.0241356682 | 0.083558011 | -0.1449517842 | 0.0026891892 |
| act(p(MGI:Ror2)) | -0.081648852 | 5.0E-10 | -0.1860442798 | 0.0 |
| act(p(MGI:Rps6)) | 0.0771732184 | 0.0 | 0.1504923429 | 0.0 |
| act(p(MGI:S100b)) | -0.0539722749 | 2.287E-7 | -0.022414233 | 0.0661842697 |
| act(p(MGI:S1pr1)) | 0.0335915712 | 0.3561719745 | 0.1424182887 | 0.0 |
| act(p(MGI:Sirt1)) | -0.0340849916 | 0.0159839357 | -0.1003571984 | 0.0 |
| act(p(MGI:Slc1a1)) | 0.0688469823 | 2.66E-8 | 0.1516526603 | 0.0 |
| act(p(MGI:Sod2)) | 0.0432862865 | 0.0305087108 | 0.1099914533 | 0.0 |
| act(p(MGI:Sp1)) | 0.0517941197 | 2.9193E-6 | 0.1410221787 | 0.0026891892 |
| act(p(MGI:Tgm1)) | -0.0658532607 | 0.0037725118 | -0.1951820354 | 0.0 |
| act(p(MGI:Thbs1)) | -0.047067308 | 0.010248927 | -0.1580949375 | 0.0 |
| act(p(MGI:Timp1)) | 0.0402396513 | 1.835359E-4 | 0.0859165422 | 0.0072804878 |
| act(p(MGI:Tlr2)) | 0.1919393757 | 0.0 | 0.3723013755 | 0.0 |
| act(p(MGI:Tlr3)) | 0.0329359491 | 0.0818111111 | 0.215266754 | 0.0 |
| act(p(MGI:Tlr4)) | 0.082460034 | 0.0305087108 | 0.2112913002 | 0.0 |
| act(p(MGI:Tlr5)) | 0.0857068495 | 0.0037725118 | 0.1974440615 | 0.0 |
| act(p(MGI:Tlr7)) | 0.0468101018 | 0.2115131846 | 0.1979038184 | 0.0 |
| act(p(MGI:Tnf)) | 0.0924077711 | 0.0 | 0.2616676513 | 0.0 |
| act(p(MGI:Tnfrsf1b)) | 0.0516203758 | 3.1141E-6 | 0.1104831073 | 0.0 |
| act(p(MGI:Traf3ip2)) | 0.3990199121 | 0.0 | 0.5451017321 | 0.0 |
| act(p(MGI:Traf6)) | 0.0053203112 | 0.3401235476 | 0.1342943594 | 0.0 |
| act(p(MGI:Trp53)) | 0.0119501879 | 0.1506195462 | 0.1603879877 | 0.0 |
| act(p(MGI:Tsc2)) | -0.051722846 | 4.7188E-6 | -0.0796055085 | 0.0115362319 |
| act(p(MGI:Tyk2)) | 0.0366445449 | 0.0511897106 | 0.1599160453 | 0.0 |
| act(p(PMIPFAM:"Cannabinoid Receptor Family")) | -0.0769516356 | 0.0 | -0.0980243162 | 0.0 |
| act(p(PMIPFAM:"ITPR Family")) | 0.0320587618 | 0.1648730512 | 0.1401588499 | 0.0 |
| act(p(SFAM:"ADRB Family")) | 0.0041950731 | 0.9684332494 | -0.0783786605 | 0.0072804878 |
| act(p(SFAM:"CREB Family")) | -0.3244052541 | 1.0E-10 | -0.619700942 | 0.0072804878 |
| act(p(SFAM:"DRD Family")) | -0.0178449018 | 0.1913787234 | 0.2132457507 | 0.0 |
| act(p(SFAM:"MAPK p38 Family")) | 0.0883310155 | 0.0 | 0.1769243228 | 0.0 |
| act(p(SFAM:"PDE4 Family")) | 0.0310257923 | 0.0068917749 | 0.0796455337 | 0.0135681818 |
| act(p(SFAM:"PRKC Family")) | 0.1257810698 | 0.0 | 0.37866909 | 0.0 |
| act(p(SFAM:"ROCK Family")) | -0.0279482882 | 0.1537103448 | -0.1297412165 | 0.0050700637 |
| act(p(SFAM:"TNFRSF Family")) | 0.10735181 | 0.0 | 0.2530888443 | 0.0 |
| complex(GOCC:"AMP-activated protein kinase complex") | 0.0115941438 | 0.1474315705 | 0.1691289395 | 0.0072804878 |
| complex(GOCC:"IkappaB kinase complex") | 0.0821803842 | 0.0 | 0.2035536424 | 0.0 |
| complex(GOCC:"laminin complex") | -0.1658892686 | 0.0037725118 | -0.2728483083 | 0.0026891892 |
| m(MGI:Mir155) | 0.0480531012 | 1.36425E-5 | 0.0966908561 | 0.0 |


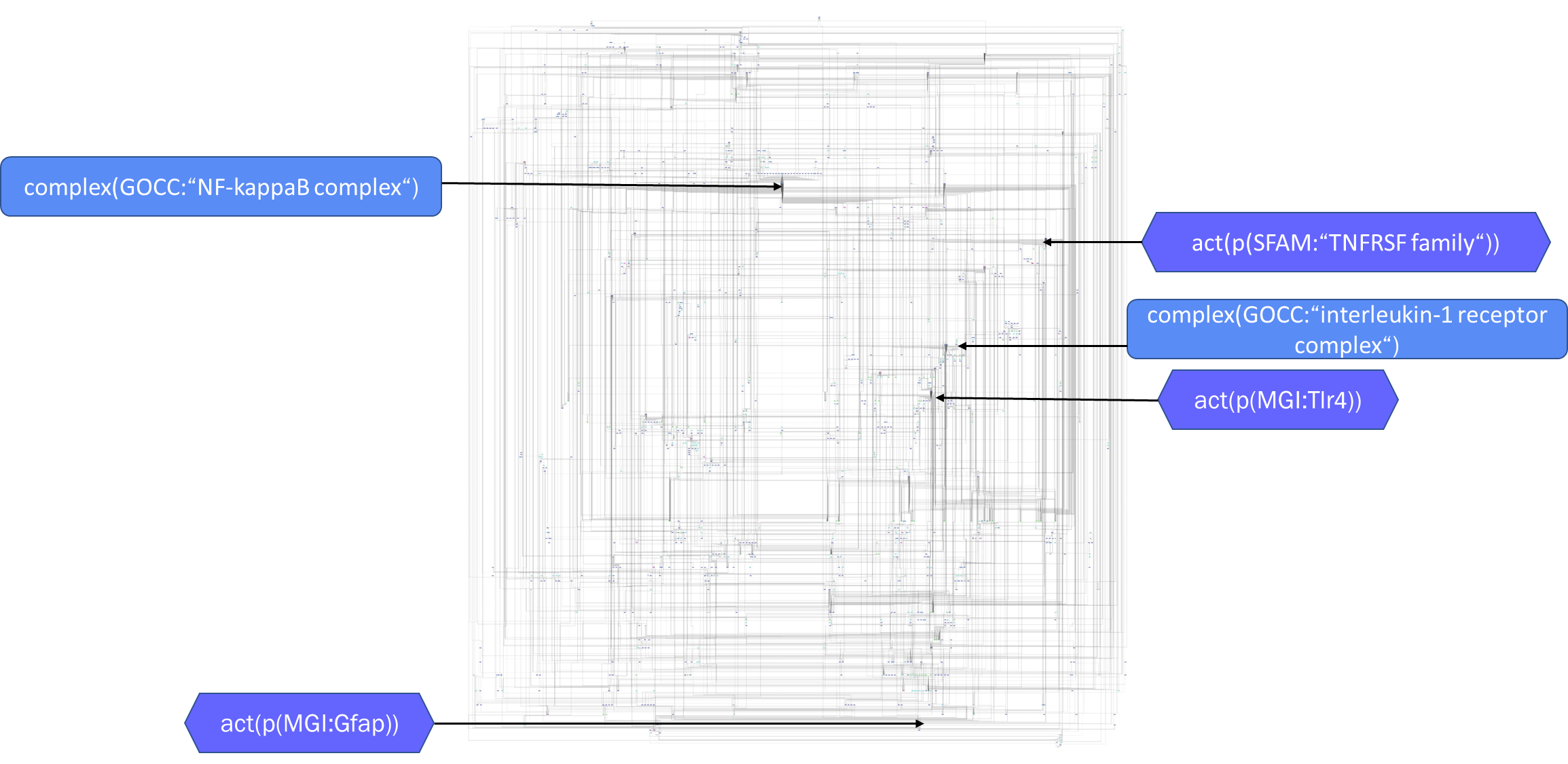


**Supplementary Figure 1**. The astrocyte activity CBN. The inserts show the highly connected TLR4 receptor, interleukin-1 receptor complex, TNFRSF family, GFAP, and the NF-κB complex nodes. act(…), protein activity (dark blue hexagon); complex(…), activity of a protein complex (medium-blue rounded rectangle); GOCC, Gene Ontology Cellular Complex database; MGI, Mouse Genome Informatics database; SFAM, protein family database.

**Supplementary list 1:**

References used to construct the astrocyte activity CBN:

1: Abdelli LS, Samsam A, Naser SA. Propionic Acid Induces Gliosis and Neuro-

inflammation through Modulation of PTEN/AKT Pathway in Autism Spectrum Disorder.

Sci Rep. 2019 Jun 19;9(1):8824. doi: 10.1038/s41598-019-45348-z. PMID: 31217543;

PMCID: PMC6584527.

2: Acaz-Fonseca E, Ortiz-Rodriguez A, Azcoitia I, Garcia-Segura LM, Arevalo MA.

Notch signaling in astrocytes mediates their morphological response to an

inflammatory challenge. Cell Death Discov. 2019 Apr 3;5:85. doi:

10.1038/s41420-019-0166-6. PMID: 30962951; PMCID: PMC6447583.

3: Agnihotri S, Wolf A, Picard D, Hawkins C, Guha A. GATA4 is a regulator of

astrocyte cell proliferation and apoptosis in the human and murine central

nervous system. Oncogene. 2009 Aug 27;28(34):3033-46. doi: 10.1038/onc.2009.159.

Epub 2009 Jun 22. PMID: 19543315.

4: Aleshin S, Grabeklis S, Hanck T, Sergeeva M, Reiser G. Peroxisome

proliferator-activated receptor (PPAR)-gamma positively controls and PPARalpha

negatively controls cyclooxygenase-2 expression in rat brain astrocytes through

a convergence on PPARbeta/delta via mutual control of PPAR expression levels.

Mol Pharmacol. 2009 Aug;76(2):414-24. doi: 10.1124/mol.109.056010. Epub 2009 May

29. PMID: 19483106.

5: Allnoch L, Baumgärtner W, Hansmann F. Impact of Astrocyte Depletion upon

Inflammation and Demyelination in a Murine Animal Model of Multiple Sclerosis.

Int J Mol Sci. 2019 Aug 12;20(16):3922. doi: 10.3390/ijms20163922. PMID:

31409036; PMCID: PMC6719128.

6: Barreto GE, White RE, Xu L, Palm CJ, Giffard RG. Effects of heat shock

protein 72 (Hsp72) on evolution of astrocyte activation following stroke in the

mouse. Exp Neurol. 2012 Dec;238(2):284-96. doi: 10.1016/j.expneurol.2012.08.015.

Epub 2012 Aug 20. PMID: 22940431; PMCID: PMC3498584.

7: Basso M, Berlin J, Xia L, Sleiman SF, Ko B, Haskew-Layton R, Kim E, Antonyak

MA, Cerione RA, Iismaa SE, Willis D, Cho S, Ratan RR. Transglutaminase

inhibition protects against oxidative stress-induced neuronal death downstream

of pathological ERK activation. J Neurosci. 2012 May 9;32(19):6561-9. doi:

10.1523/JNEUROSCI.3353-11.2012. Erratum in: J Neurosci. 2012 Aug 8;32(32):11157.

PMID: 22573678; PMCID: PMC3444816.

8: Beck H, Semisch M, Culmsee C, Plesnila N, Hatzopoulos AK. Egr-1 regulates

expression of the glial scar component phosphacan in astrocytes after

experimental stroke. Am J Pathol. 2008 Jul;173(1):77-92. doi:

10.2353/ajpath.2008.070648. Epub 2008 Jun 13. PMID: 18556777; PMCID: PMC2438287.

9: Ben Haim L, Ceyzériat K, Carrillo-de Sauvage MA, Aubry F, Auregan G,

Guillermier M, Ruiz M, Petit F, Houitte D, Faivre E, Vandesquille M, Aron-Badin

R, Dhenain M, Déglon N, Hantraye P, Brouillet E, Bonvento G, Escartin C. The

JAK/STAT3 pathway is a common inducer of astrocyte reactivity in Alzheimer's and

Huntington's diseases. J Neurosci. 2015 Feb 11;35(6):2817-29. doi:

10.1523/JNEUROSCI.3516-14.2015. PMID: 25673868; PMCID: PMC6605603.

10: Benner EJ, Luciano D, Jo R, Abdi K, Paez-Gonzalez P, Sheng H, Warner DS, Liu

C, Eroglu C, Kuo CT. Protective astrogenesis from the SVZ niche after injury is

controlled by Notch modulator Thbs4. Nature. 2013 May 16;497(7449):369-73. doi:

10.1038/nature12069. Epub 2013 Apr 24. PMID: 23615612; PMCID: PMC3667629.

11: Bhardwaj R, Yester JW, Singh SK, Biswas DD, Surace MJ, Waters MR, Hauser KF,

Yao Z, Boyce BF, Kordula T. RelB/p50 complexes regulate cytokine-induced YKL-40

expression. J Immunol. 2015 Mar 15;194(6):2862-70. doi:

10.4049/jimmunol.1400874. Epub 2015 Feb 13. PMID: 25681350; PMCID: PMC4355396.

12: Bhat SA, Goel R, Shukla R, Hanif K. Platelet CD40L induces activation of

astrocytes and microglia in hypertension. Brain Behav Immun. 2017

Jan;59:173-189. doi: 10.1016/j.bbi.2016.09.021. Epub 2016 Sep 19. PMID:

27658543.

13: Bi F, Huang C, Tong J, Qiu G, Huang B, Wu Q, Li F, Xu Z, Bowser R, Xia XG,

Zhou H. Reactive astrocytes secrete lcn2 to promote neuron death. Proc Natl Acad

Sci U S A. 2013 Mar 5;110(10):4069-74. doi: 10.1073/pnas.1218497110. Epub 2013

Feb 19. PMID: 23431168; PMCID: PMC3593910.

14: Bombeiro AL, Hell RC, Simões GF, Castro MV, Oliveira AL. Importance of major

histocompatibility complex of class I (MHC-I) expression for astroglial

reactivity and stability of neural circuits in vitro. Neurosci Lett. 2017 Apr

24;647:97-103. doi: 10.1016/j.neulet.2017.03.038. Epub 2017 Mar 21. PMID:

28341478.

15: Brambilla R, Bracchi-Ricard V, Hu WH, Frydel B, Bramwell A, Karmally S,

Green EJ, Bethea JR. Inhibition of astroglial nuclear factor kappaB reduces

inflammation and improves functional recovery after spinal cord injury. J Exp

Med. 2005 Jul 4;202(1):145-56. doi: 10.1084/jem.20041918. PMID: 15998793; PMCID:

PMC2212896.

16: Brinkmann V. FTY720 (fingolimod) in Multiple Sclerosis: therapeutic effects

in the immune and the central nervous system. Br J Pharmacol. 2009

Nov;158(5):1173-82. doi: 10.1111/j.1476-5381.2009.00451.x. Epub 2009 Oct 8.

PMID: 19814729; PMCID: PMC2782328.

17: Butchi NB, Du M, Peterson KE. Interactions between TLR7 and TLR9 agonists

and receptors regulate innate immune responses by astrocytes and microglia.

Glia. 2010 Apr 15;58(6):650-64. doi: 10.1002/glia.20952. PMID: 19998480; PMCID:

PMC3767435.

18: Caruso C, Durand D, Schiöth HB, Rey R, Seilicovich A, Lasaga M. Activation

of melanocortin 4 receptors reduces the inflammatory response and prevents

apoptosis induced by lipopolysaccharide and interferon-gamma in astrocytes.

Endocrinology. 2007 Oct;148(10):4918-26. doi: 10.1210/en.2007-0366. Epub 2007

Jun 26. PMID: 17595227.

19: Catalano M, Lauro C, Cipriani R, Chece G, Ponzetta A, Di Angelantonio S,

Ragozzino D, Limatola C. CX3CL1 protects neurons against excitotoxicity

enhancing GLT-1 activity on astrocytes. J Neuroimmunol. 2013 Oct

15;263(1-2):75-82. doi: 10.1016/j.jneuroim.2013.07.020. Epub 2013 Aug 8. PMID:

23968561.

20: Chao CC, Gutiérrez-Vázquez C, Rothhammer V, Mayo L, Wheeler MA, Tjon EC,

Zandee SEJ, Blain M, de Lima KA, Takenaka MC, Avila-Pacheco J, Hewson P, Liu L,

Sanmarco LM, Borucki DM, Lipof GZ, Trauger SA, Clish CB, Antel JP, Prat A,

Quintana FJ. Metabolic Control of Astrocyte Pathogenic Activity via cPLA2-MAVS.

Cell. 2019 Dec 12;179(7):1483-1498.e22. doi: 10.1016/j.cell.2019.11.016. Epub

2019 Dec 5. PMID: 31813625; PMCID: PMC6936326.

21: Chapouly C, Tadesse Argaw A, Horng S, Castro K, Zhang J, Asp L, Loo H,

Laitman BM, Mariani JN, Straus Farber R, Zaslavsky E, Nudelman G, Raine CS, John

GR. Astrocytic TYMP and VEGFA drive blood-brain barrier opening in inflammatory

central nervous system lesions. Brain. 2015 Jun;138(Pt 6):1548-67. doi:

10.1093/brain/awv077. Epub 2015 Mar 23. PMID: 25805644; PMCID: PMC4614128.

22: Cheng Y, Takeuchi H, Sonobe Y, Jin S, Wang Y, Horiuchi H, Parajuli B,

Kawanokuchi J, Mizuno T, Suzumura A. Sirtuin 1 attenuates oxidative stress via

upregulation of superoxide dismutase 2 and catalase in astrocytes. J

Neuroimmunol. 2014 Apr 15;269(1-2):38-43. doi: 10.1016/j.jneuroim.2014.02.001.

Epub 2014 Feb 12. PMID: 24565075.

23: Chen J, Wang ZZ, Zuo W, Zhang S, Chu SF, Chen NH. Effects of chronic mild

stress on behavioral and neurobiological parameters - Role of glucocorticoid.

Horm Behav. 2016 Feb;78:150-9. doi: 10.1016/j.yhbeh.2015.11.006. Epub 2015 Nov

22. PMID: 26592454.

24: Chen J, He W, Hu X, Shen Y, Cao J, Wei Z, Luan Y, He L, Jiang F, Tao Y. A

role for ErbB signaling in the induction of reactive astrogliosis. Cell Discov.

2017 Dec 5;3:17044. doi: 10.1038/celldisc.2017.44. PMID: 29238610; PMCID:

PMC5717352.

25: Chen X, Wang H, Zhou M, Li X, Fang Z, Gao H, Li Y, Hu W. Valproic Acid

Attenuates Traumatic Brain Injury-Induced Inflammation <i>in Vivo</i>:

Involvement of Autophagy and the Nrf2/ARE Signaling Pathway. Front Mol Neurosci.

2018 Apr 17;11:117. doi: 10.3389/fnmol.2018.00117. PMID: 29719500; PMCID:

PMC5913341.

26: Chen X, Zhang X, Wang Y, Lei H, Su H, Zeng J, Pei Z, Huang R. Inhibition of

immunoproteasome reduces infarction volume and attenuates inflammatory reaction

in a rat model of ischemic stroke. Cell Death Dis. 2015 Jan 29;6(1):e1626. doi:

10.1038/cddis.2014.586. PMID: 25633295; PMCID: PMC4669779.

27: Chen Y, Miles DK, Hoang T, Shi J, Hurlock E, Kernie SG, Lu QR. The basic

helix-loop-helix transcription factor olig2 is critical for reactive astrocyte

proliferation after cortical injury. J Neurosci. 2008 Oct 22;28(43):10983-9.

doi: 10.1523/JNEUROSCI.3545-08.2008. PMID: 18945906; PMCID: PMC2631240.

28: Chistyakov DV, Aleshin S, Sergeeva MG, Reiser G. Regulation of peroxisome

proliferator-activated receptor β/δ expression and activity levels by toll-like

receptor agonists and MAP kinase inhibitors in rat astrocytes. J Neurochem. 2014

Aug;130(4):563-74. doi: 10.1111/jnc.12757. Epub 2014 May 28. PMID: 24806616.

29: Choi DJ, An J, Jou I, Park SM, Joe EH. A Parkinson's disease gene, DJ-1,

regulates anti-inflammatory roles of astrocytes through prostaglandin

D<sub>2</sub> synthase expression. Neurobiol Dis. 2019 Jul;127:482-491. doi:

10.1016/j.nbd.2019.04.003. Epub 2019 Apr 4. PMID: 30954702.

30: Choi SS, Lee HJ, Lim I, Satoh J, Kim SU. Human astrocytes: secretome

profiles of cytokines and chemokines. PLoS One. 2014 Apr 1;9(4):e92325. doi:

10.1371/journal.pone.0092325. PMID: 24691121; PMCID: PMC3972155.

31: Choi WH, Ji KA, Jeon SB, Yang MS, Kim H, Min KJ, Shong M, Jou I, Joe EH.

Anti-inflammatory roles of retinoic acid in rat brain astrocytes: Suppression of

interferon-gamma-induced JAK/STAT phosphorylation. Biochem Biophys Res Commun.

2005 Apr 1;329(1):125-31. doi: 10.1016/j.bbrc.2005.01.110. PMID: 15721283.

32: Choi YK, Kim JH, Lee DK, Lee KS, Won MH, Jeoung D, Lee H, Ha KS, Kwon YG,

Kim YM. Carbon Monoxide Potentiation of L-Type Ca<sup>2+</sup> Channel Activity

Increases HIF-1α-Independent VEGF Expression via an AMPKα/SIRT1-Mediated

PGC-1α/ERRα Axis. Antioxid Redox Signal. 2017 Jul 1;27(1):21-36. doi:

10.1089/ars.2016.6684. Epub 2016 Sep 28. PMID: 27554679.

33: Christiansen SH, Selige J, Dunkern T, Rassov A, Leist M. Combined anti-

inflammatory effects of β2-adrenergic agonists and PDE4 inhibitors on astrocytes

by upregulation of intracellular cAMP. Neurochem Int. 2011 Nov;59(6):837-46.

doi: 10.1016/j.neuint.2011.08.012. Epub 2011 Aug 17. PMID: 21871511.

34: Cirillo C, Capoccia E, Iuvone T, Cuomo R, Sarnelli G, Steardo L, Esposito G.

S100B Inhibitor Pentamidine Attenuates Reactive Gliosis and Reduces Neuronal

Loss in a Mouse Model of Alzheimer's Disease. Biomed Res Int. 2015;2015:508342.

doi: 10.1155/2015/508342. Epub 2015 Jul 29. PMID: 26295040; PMCID: PMC4532807.

35: Cirillo G, Bianco MR, Colangelo AM, Cavaliere C, Daniele de L, Zaccaro L,

Alberghina L, Papa M. Reactive astrocytosis-induced perturbation of synaptic

homeostasis is restored by nerve growth factor. Neurobiol Dis. 2011

Mar;41(3):630-9. doi: 10.1016/j.nbd.2010.11.012. Epub 2010 Nov 25. PMID:

21111819.

36: Clark IC, Gutiérrez-Vázquez C, Wheeler MA, Li Z, Rothhammer V, Linnerbauer

M, Sanmarco LM, Guo L, Blain M, Zandee SEJ, Chao CC, Batterman KV, Schwabenland

M, Lotfy P, Tejeda-Velarde A, Hewson P, Manganeli Polonio C, Shultis MW, Salem

Y, Tjon EC, Fonseca-Castro PH, Borucki DM, Alves de Lima K, Plasencia A, Abate

AR, Rosene DL, Hodgetts KJ, Prinz M, Antel JP, Prat A, Quintana FJ. Barcoded

viral tracing of single-cell interactions in central nervous system

inflammation. Science. 2021 Apr 23;372(6540):eabf1230. doi:

10.1126/science.abf1230. PMID: 33888612; PMCID: PMC8157482.

37: Codeluppi S, Svensson CI, Hefferan MP, Valencia F, Silldorff MD, Oshiro M,

Marsala M, Pasquale EB. The Rheb-mTOR pathway is upregulated in reactive

astrocytes of the injured spinal cord. J Neurosci. 2009 Jan 28;29(4):1093-104.

doi: 10.1523/JNEUROSCI.4103-08.2009. PMID: 19176818; PMCID: PMC2682457.

38: Codeluppi S, Fernandez-Zafra T, Sandor K, Kjell J, Liu Q, Abrams M, Olson L,

Gray NS, Svensson CI, Uhlén P. Interleukin-6 secretion by astrocytes is

dynamically regulated by PI3K-mTOR-calcium signaling. PLoS One. 2014 Mar

25;9(3):e92649. doi: 10.1371/journal.pone.0092649. PMID: 24667246; PMCID:

PMC3965459.

39: Cooley ID, Chauhan VS, Donneyz MA, Marriott I. Astrocytes produce IL-19 in

response to bacterial challenge and are sensitive to the immunosuppressive

effects of this IL-10 family member. Glia. 2014 May;62(5):818-28. doi:

10.1002/glia.22644. PMID: 24677051; PMCID: PMC4076830.

40: Coulson-Thomas VJ, Lauer ME, Soleman S, Zhao C, Hascall VC, Day AJ, Fawcett

JW. Tumor Necrosis Factor-stimulated Gene-6 (TSG-6) Is Constitutively Expressed

in Adult Central Nervous System (CNS) and Associated with Astrocyte-mediated

Glial Scar Formation following Spinal Cord Injury. J Biol Chem. 2016 Sep

16;291(38):19939-52. doi: 10.1074/jbc.M115.710673. Epub 2016 Jul 19. PMID:

27435674; PMCID: PMC5025681.

41: Crocker SJ, Frausto RF, Whitton JL, Milner R. A novel method to establish

microglia-free astrocyte cultures: comparison of matrix metalloproteinase

expression profiles in pure cultures of astrocytes and microglia. Glia. 2008 Aug

15;56(11):1187-98. doi: 10.1002/glia.20689. PMID: 18449943; PMCID: PMC2776034.

42: Cuevas-Diaz Duran R, Wang CY, Zheng H, Deneen B, Wu JQ. Brain Region-

Specific Gene Signatures Revealed by Distinct Astrocyte Subpopulations Unveil

Links to Glioma and Neurodegenerative Diseases. eNeuro. 2019 Apr

2;6(2):ENEURO.0288-18.2019. doi: 10.1523/ENEURO.0288-18.2019. PMID: 30957015;

PMCID: PMC6449165.

43: Dai B, Yan T, Shen YX, Xu YJ, Shen HB, Chen D, Wang JR, He SH, Dong QR,

Zhang AL. Edaravone protects against oxygen-glucose-serum

deprivation/restoration-induced apoptosis in spinal cord astrocytes by

inhibiting integrated stress response. Neural Regen Res. 2017 Feb;12(2):283-289.

doi: 10.4103/1673-5374.199006. PMID: 28400812; PMCID: PMC5361514.

44: Ding J, Yu JZ, Li QY, Wang X, Lu CZ, Xiao BG. Rho kinase inhibitor Fasudil

induces neuroprotection and neurogenesis partially through astrocyte-derived

G-CSF. Brain Behav Immun. 2009 Nov;23(8):1083-8. doi: 10.1016/j.bbi.2009.05.002.

Epub 2009 May 15. PMID: 19447168.

45: Dostal CR, Gamsby NS, Lawson MA, McCusker RH. Glia- and tissue-specific

changes in the Kynurenine Pathway after treatment of mice with

lipopolysaccharide and dexamethasone. Brain Behav Immun. 2018 Mar;69:321-335.

doi: 10.1016/j.bbi.2017.12.006. Epub 2017 Dec 11. PMID: 29241670; PMCID:

PMC5857427.

46: Doyle KP, Cekanaviciute E, Mamer LE, Buckwalter MS. TGFβ signaling in the

brain increases with aging and signals to astrocytes and innate immune cells in

the weeks after stroke. J Neuroinflammation. 2010 Oct 11;7:62. doi:

10.1186/1742-2094-7-62. PMID: 20937129; PMCID: PMC2958905.

47: Dresselhaus E, Duerr JM, Vincent F, Sylvain EK, Beyna M, Lanyon LF,

LaChapelle E, Pettersson M, Bales KR, Ramaswamy G. Class I HDAC inhibition is a

novel pathway for regulating astrocytic apoE secretion. PLoS One. 2018 Mar

26;13(3):e0194661. doi: 10.1371/journal.pone.0194661. PMID: 29579087; PMCID:

PMC5868809.

48: Dumont AO, Goursaud S, Desmet N, Hermans E. Differential regulation of

glutamate transporter subtypes by pro-inflammatory cytokine TNF-α in cortical

astrocytes from a rat model of amyotrophic lateral sclerosis. PLoS One. 2014 May

16;9(5):e97649. doi: 10.1371/journal.pone.0097649. PMID: 24836816; PMCID:

PMC4023965.

49: Dusaban SS, Chun J, Rosen H, Purcell NH, Brown JH. Sphingosine 1-phosphate

receptor 3 and RhoA signaling mediate inflammatory gene expression in

astrocytes. J Neuroinflammation. 2017 Jun 2;14(1):111. doi:

10.1186/s12974-017-0882-x. PMID: 28577576; PMCID: PMC5455202.

50: Endo M, Ubulkasim G, Kobayashi C, Onishi R, Aiba A, Minami Y. Critical role

of Ror2 receptor tyrosine kinase in regulating cell cycle progression of

reactive astrocytes following brain injury. Glia. 2017 Jan;65(1):182-197. doi:

10.1002/glia.23086. Epub 2016 Oct 11. PMID: 27726178.

51: Erschbamer MK, Hofstetter CP, Olson L. RhoA, RhoB, RhoC, Rac1, Cdc42, and

Tc10 mRNA levels in spinal cord, sensory ganglia, and corticospinal tract

neurons and long-lasting specific changes following spinal cord injury. J Comp

Neurol. 2005 Apr 4;484(2):224-33. doi: 10.1002/cne.20471. PMID: 15736231.

52: Etienne-Manneville S, Hall A. Cdc42 regulates GSK-3beta and adenomatous

polyposis coli to control cell polarity. Nature. 2003 Feb 13;421(6924):753-6.

doi: 10.1038/nature01423. Epub 2003 Jan 29. PMID: 12610628.

53: Etienne-Manneville S, Hall A. Integrin-mediated activation of Cdc42 controls

cell polarity in migrating astrocytes through PKCzeta. Cell. 2001 Aug

24;106(4):489-98. doi: 10.1016/s0092-8674(01)00471-8. PMID: 11525734.

54: Facci L, Barbierato M, Marinelli C, Argentini C, Skaper SD, Giusti P. Toll-

like receptors 2, -3 and -4 prime microglia but not astrocytes across central

nervous system regions for ATP-dependent interleukin-1β release. Sci Rep. 2014

Oct 29;4:6824. doi: 10.1038/srep06824. PMID: 25351234; PMCID: PMC5381369.

55: Fang Z, Duthoit N, Wicher G, Källskog O, Ambartsumian N, Lukanidin E,

Takenaga K, Kozlova EN. Intracellular calcium-binding protein S100A4 influences

injury-induced migration of white matter astrocytes. Acta Neuropathol. 2006

Mar;111(3):213-9. doi: 10.1007/s00401-005-0019-7. Epub 2006 Feb 4. PMID:

16463066.

56: Feng D, Guo B, Liu G, Wang B, Wang W, Gao G, Qin H, Wu S. FGF2 alleviates

PTSD symptoms in rats by restoring GLAST function in astrocytes via the JAK/STAT

pathway. Eur Neuropsychopharmacol. 2015 Aug;25(8):1287-99. doi:

10.1016/j.euroneuro.2015.04.020. Epub 2015 May 5. PMID: 25979764.

57: Fernandes A, Falcão AS, Silva RF, Gordo AC, Gama MJ, Brito MA, Brites D.

Inflammatory signalling pathways involved in astroglial activation by

unconjugated bilirubin. J Neurochem. 2006 Mar;96(6):1667-79. doi:

10.1111/j.1471-4159.2006.03680.x. Epub 2006 Feb 10. PMID: 16476078.

58: Fischer I, Alliod C, Martinier N, Newcombe J, Brana C, Pouly S. Sphingosine

kinase 1 and sphingosine 1-phosphate receptor 3 are functionally upregulated on

astrocytes under pro-inflammatory conditions. PLoS One. 2011;6(8):e23905. doi:

10.1371/journal.pone.0023905. Epub 2011 Aug 24. PMID: 21887342; PMCID:

PMC3161076.

59: Fujita A, Yamaguchi H, Yamasaki R, Cui Y, Matsuoka Y, Yamada KI, Kira JI.

Connexin 30 deficiency attenuates A2 astrocyte responses and induces severe

neurodegeneration in a 1-methyl-4-phenyl-1,2,3,6-tetrahydropyridine

hydrochloride Parkinson's disease animal model. J Neuroinflammation. 2018 Aug

13;15(1):227. doi: 10.1186/s12974-018-1251-0. PMID: 30103794; PMCID: PMC6090688.

60: Gadea A, Schinelli S, Gallo V. Endothelin-1 regulates astrocyte

proliferation and reactive gliosis via a JNK/c-Jun signaling pathway. J

Neurosci. 2008 Mar 5;28(10):2394-408. doi: 10.1523/JNEUROSCI.5652-07.2008. PMID:

18322086; PMCID: PMC2695974.

61: Gallagher PE, Chappell MC, Ferrario CM, Tallant EA. Distinct roles for ANG

II and ANG-(1-7) in the regulation of angiotensin-converting enzyme 2 in rat

astrocytes. Am J Physiol Cell Physiol. 2006 Feb;290(2):C420-6. doi:

10.1152/ajpcell.00409.2004. Epub 2005 Sep 21. PMID: 16176966.

62: Galou M, Colucci-Guyon E, Ensergueix D, Ridet JL, Gimenez y Ribotta M,

Privat A, Babinet C, Dupouey P. Disrupted glial fibrillary acidic protein

network in astrocytes from vimentin knockout mice. J Cell Biol. 1996

May;133(4):853-63. doi: 10.1083/jcb.133.4.853. PMID: 8666670; PMCID: PMC2120844.

63: Gao K, Wang CR, Jiang F, Wong AY, Su N, Jiang JH, Chai RC, Vatcher G, Teng

J, Chen J, Jiang YW, Yu AC. Traumatic scratch injury in astrocytes triggers

calcium influx to activate the JNK/c-Jun/AP-1 pathway and switch on GFAP

expression. Glia. 2013 Dec;61(12):2063-77. doi: 10.1002/glia.22577. Epub 2013

Oct 7. PMID: 24123203.

64: Garwood CJ, Ratcliffe LE, Morgan SV, Simpson JE, Owens H, Vazquez-Villaseñor

I, Heath PR, Romero IA, Ince PG, Wharton SB. Insulin and IGF1 signalling

pathways in human astrocytes in vitro and in vivo; characterisation, subcellular

localisation and modulation of the receptors. Mol Brain. 2015 Aug 22;8:51. doi:

10.1186/s13041-015-0138-6. PMID: 26297026; PMCID: PMC4546315.

65: Gessi S, Merighi S, Stefanelli A, Fazzi D, Varani K, Borea PA. A(1) and A(3)

adenosine receptors inhibit LPS-induced hypoxia-inducible factor-1 accumulation

in murine astrocytes. Pharmacol Res. 2013 Oct;76:157-70. doi:

10.1016/j.phrs.2013.08.002. Epub 2013 Aug 19. PMID: 23969284.

66: Gironacci MM, Vatta M, Rodriguez-Fermepín M, Fernández BE, Peña C.

Angiotensin-(1-7) reduces norepinephrine release through a nitric oxide

mechanism in rat hypothalamus. Hypertension. 2000 Jun;35(6):1248-52. doi:

10.1161/01.hyp.35.6.1248. PMID: 10856272.

67: Gorina R, Font-Nieves M, Márquez-Kisinousky L, Santalucia T, Planas AM.

Astrocyte TLR4 activation induces a proinflammatory environment through the

interplay between MyD88-dependent NFκB signaling, MAPK, and Jak1/Stat1 pathways.

Glia. 2011 Feb;59(2):242-55. doi: 10.1002/glia.21094. PMID: 21125645.

68: Goto J, Tezuka T, Nakazawa T, Sagara H, Yamamoto T. Loss of Fyn tyrosine

kinase on the C57BL/6 genetic background causes hydrocephalus with defects in

oligodendrocyte development. Mol Cell Neurosci. 2008 Jun;38(2):203-12. doi:

10.1016/j.mcn.2008.02.009. Epub 2008 Mar 4. PMID: 18403215.

69: Griffin P, Dimitry JM, Sheehan PW, Lananna BV, Guo C, Robinette ML, Hayes

ME, Cedeño MR, Nadarajah CJ, Ezerskiy LA, Colonna M, Zhang J, Bauer AQ, Burris

TP, Musiek ES. Circadian clock protein Rev-erbα regulates neuroinflammation.

Proc Natl Acad Sci U S A. 2019 Mar 12;116(11):5102-5107. doi:

10.1073/pnas.1812405116. Epub 2019 Feb 21. PMID: 30792350; PMCID: PMC6421453.

70: Gris P, Tighe A, Levin D, Sharma R, Brown A. Transcriptional regulation of

scar gene expression in primary astrocytes. Glia. 2007 Aug 15;55(11):1145-55.

doi: 10.1002/glia.20537. PMID: 17597120.

71: Guo X, Dason ES, Zanon-Moreno V, Jiang Q, Nahirnyj A, Chan D, Flanagan JG,

Sivak JM. PGC-1α signaling coordinates susceptibility to metabolic and oxidative

injury in the inner retina. Am J Pathol. 2014 Apr;184(4):1017-1029. doi:

10.1016/j.ajpath.2013.12.012. Epub 2014 Feb 5. PMID: 24508229.

72: Guttenplan KA, Weigel MK, Prakash P, Wijewardhane PR, Hasel P, Rufen-

Blanchette U, Münch AE, Blum JA, Fine J, Neal MC, Bruce KD, Gitler AD, Chopra G,

Liddelow SA, Barres BA. Neurotoxic reactive astrocytes induce cell death via

saturated lipids. Nature. 2021 Nov;599(7883):102-107. doi:

10.1038/s41586-021-03960-y. Epub 2021 Oct 6. PMID: 34616039.

73: Hagmeyer S, Romão MA, Cristóvão JS, Vilella A, Zoli M, Gomes CM, Grabrucker

AM. Distribution and Relative Abundance of S100 Proteins in the Brain of the

APP23 Alzheimer's Disease Model Mice. Front Neurosci. 2019 Jun 20;13:640. doi:

10.3389/fnins.2019.00640. PMID: 31281238; PMCID: PMC6596341.

74: Hara M, Kobayakawa K, Ohkawa Y, Kumamaru H, Yokota K, Saito T, Kijima K,

Yoshizaki S, Harimaya K, Nakashima Y, Okada S. Interaction of reactive

astrocytes with type I collagen induces astrocytic scar formation through the

integrin-N-cadherin pathway after spinal cord injury. Nat Med. 2017

Jul;23(7):818-828. doi: 10.1038/nm.4354. Epub 2017 Jun 19. PMID: 28628111.

75: Haroon F, Drögemüller K, Händel U, Brunn A, Reinhold D, Nishanth G, Mueller

W, Trautwein C, Ernst M, Deckert M, Schlüter D. Gp130-dependent astrocytic

survival is critical for the control of autoimmune central nervous system

inflammation. J Immunol. 2011 Jun 1;186(11):6521-31. doi:

10.4049/jimmunol.1001135. Epub 2011 Apr 22. PMID: 21515788.

76: Hennessy E, Griffin ÉW, Cunningham C. Astrocytes Are Primed by Chronic

Neurodegeneration to Produce Exaggerated Chemokine and Cell Infiltration

Responses to Acute Stimulation with the Cytokines IL-1β and TNF-α. J Neurosci.

2015 Jun 3;35(22):8411-22. doi: 10.1523/JNEUROSCI.2745-14.2015. PMID: 26041910;

PMCID: PMC4452550.

77: Herrmann JE, Imura T, Song B, Qi J, Ao Y, Nguyen TK, Korsak RA, Takeda K,

Akira S, Sofroniew MV. STAT3 is a critical regulator of astrogliosis and scar

formation after spinal cord injury. J Neurosci. 2008 Jul 9;28(28):7231-43. doi:

10.1523/JNEUROSCI.1709-08.2008. PMID: 18614693; PMCID: PMC2583788.

78: He M, Shi X, Yang M, Yang T, Li T, Chen J. Mesenchymal stem cells-derived

IL-6 activates AMPK/mTOR signaling to inhibit the proliferation of reactive

astrocytes induced by hypoxic-ischemic brain damage. Exp Neurol. 2019

Jan;311:15-32. doi: 10.1016/j.expneurol.2018.09.006. Epub 2018 Sep 10. PMID:

30213506.

79: Hoffmann FS, Hofereiter J, Rübsamen H, Melms J, Schwarz S, Faber H, Weber P,

Pütz B, Loleit V, Weber F, Hohlfeld R, Meinl E, Krumbholz M. Fingolimod induces

neuroprotective factors in human astrocytes. J Neuroinflammation. 2015 Sep

30;12:184. doi: 10.1186/s12974-015-0393-6. PMID: 26419927; PMCID: PMC4589103.

80: Holt LM, Hernandez RD, Pacheco NL, Torres Ceja B, Hossain M, Olsen ML.

Astrocyte morphogenesis is dependent on BDNF signaling via astrocytic TrkB.T1.

Elife. 2019 Aug 21;8:e44667. doi: 10.7554/eLife.44667. PMID: 31433295; PMCID:

PMC6726422.

81: Houben E, Janssens K, Hermans D, Vandooren J, Van den Haute C, Schepers M,

Vanmierlo T, Lambrichts I, van Horssen J, Baekelandt V, Opdenakker G, Baron W,

Broux B, Slaets H, Hellings N. Oncostatin M-induced astrocytic tissue inhibitor

of metalloproteinases-1 drives remyelination. Proc Natl Acad Sci U S A. 2020 Mar

3;117(9):5028-5038. doi: 10.1073/pnas.1912910117. Epub 2020 Feb 18. PMID:

32071226; PMCID: PMC7060743.

82: Howe MD, Furr JW, Munshi Y, Roy-O'Reilly MA, Maniskas ME, Koellhoffer EC,

d'Aigle J, Sansing LH, McCullough LD, Urayama A. Transforming growth factor-β

promotes basement membrane fibrosis, alters perivascular cerebrospinal fluid

distribution, and worsens neurological recovery in the aged brain after stroke.

Geroscience. 2019 Oct;41(5):543-559. doi: 10.1007/s11357-019-00118-7. Epub 2019

Nov 13. PMID: 31721012; PMCID: PMC6885082.

83: Hsiao HY, Mak OT, Yang CS, Liu YP, Fang KM, Tzeng SF. TNF-alpha/IFN-gamma-

induced iNOS expression increased by prostaglandin E2 in rat primary astrocytes

via EP2-evoked cAMP/PKA and intracellular calcium signaling. Glia. 2007 Jan

15;55(2):214-23. doi: 10.1002/glia.20453. PMID: 17091492.

84: Ikeshima-Kataoka H, Abe Y, Abe T, Yasui M. Immunological function of

aquaporin-4 in stab-wounded mouse brain in concert with a pro-inflammatory

cytokine inducer, osteopontin. Mol Cell Neurosci. 2013 Sep;56:65-75. doi:

10.1016/j.mcn.2013.02.002. Epub 2013 Feb 18. PMID: 23428384.

85: Islam A, Kagawa Y, Miyazaki H, Shil SK, Umaru BA, Yasumoto Y, Yamamoto Y,

Owada Y. FABP7 Protects Astrocytes Against ROS Toxicity via Lipid Droplet

Formation. Mol Neurobiol. 2019 Aug;56(8):5763-5779. doi:

10.1007/s12035-019-1489-2. Epub 2019 Jan 24. PMID: 30680690.

86: Iwase K, Miyanaka K, Shimizu A, Nagasaki A, Gotoh T, Mori M, Takiguchi M.

Induction of endothelial nitric-oxide synthase in rat brain astrocytes by

systemic lipopolysaccharide treatment. J Biol Chem. 2000 Apr

21;275(16):11929-33. doi: 10.1074/jbc.275.16.11929. PMID: 10766821.

87: Iyer A, Zurolo E, Prabowo A, Fluiter K, Spliet WG, van Rijen PC, Gorter JA,

Aronica E. MicroRNA-146a: a key regulator of astrocyte-mediated inflammatory

response. PLoS One. 2012;7(9):e44789. doi: 10.1371/journal.pone.0044789. Epub

2012 Sep 13. PMID: 23028621; PMCID: PMC3441440.

88: Jiang ZS, Zhang JR. LncRNA SNHG5 enhances astrocytes and microglia viability

via upregulating KLF4 in spinal cord injury. Int J Biol Macromol. 2018

Dec;120(Pt A):66-72. doi: 10.1016/j.ijbiomac.2018.08.002. Epub 2018 Aug 1. PMID:

30076931.

89: Jones KJ, Korb E, Kundel MA, Kochanek AR, Kabraji S, McEvoy M, Shin CY,

Wells DG. CPEB1 regulates beta-catenin mRNA translation and cell migration in

astrocytes. Glia. 2008 Oct;56(13):1401-13. doi: 10.1002/glia.20707. PMID:

18618654; PMCID: PMC3013359.

90: Kanemaru K, Kubota J, Sekiya H, Hirose K, Okubo Y, Iino M. Calcium-dependent

N-cadherin up-regulation mediates reactive astrogliosis and neuroprotection

after brain injury. Proc Natl Acad Sci U S A. 2013 Jul 9;110(28):11612-7. doi:

10.1073/pnas.1300378110. Epub 2013 Jun 24. PMID: 23798419; PMCID: PMC3710861.

91: Kang W, Balordi F, Su N, Chen L, Fishell G, Hébert JM. Astrocyte activation

is suppressed in both normal and injured brain by FGF signaling. Proc Natl Acad

Sci U S A. 2014 Jul 22;111(29):E2987-95. doi: 10.1073/pnas.1320401111. Epub 2014

Jul 7. PMID: 25002516; PMCID: PMC4115557.

92: Kano SI, Choi EY, Dohi E, Agarwal S, Chang DJ, Wilson AM, Lo BD, Rose IVL,

Gonzalez S, Imai T, Sawa A. Glutathione <i>S</i>-transferases promote

proinflammatory astrocyte-microglia communication during brain inflammation. Sci

Signal. 2019 Feb 19;12(569):eaar2124. doi: 10.1126/scisignal.aar2124. PMID:

30783009; PMCID: PMC6637164.

93: Kasprowska D, Machnik G, Kost A, Gabryel B. Time-Dependent Changes in

Apoptosis Upon Autophagy Inhibition in Astrocytes Exposed to Oxygen and Glucose

Deprivation. Cell Mol Neurobiol. 2017 Mar;37(2):223-234. doi:

10.1007/s10571-016-0363-2. Epub 2016 Mar 16. PMID: 26983718.

94: Kim JH, Choi DJ, Jeong HK, Kim J, Kim DW, Choi SY, Park SM, Suh YH, Jou I,

Joe EH. DJ-1 facilitates the interaction between STAT1 and its phosphatase,

SHP-1, in brain microglia and astrocytes: A novel anti-inflammatory function of

DJ-1. Neurobiol Dis. 2013 Dec;60:1-10. doi: 10.1016/j.nbd.2013.08.007. Epub 2013

Aug 20. PMID: 23969237.

95: Kim KC, Hyun Joo S, Shin CY. CPEB1 modulates lipopolysaccharide-mediated

iNOS induction in rat primary astrocytes. Biochem Biophys Res Commun. 2011 Jun

17;409(4):687-92. doi: 10.1016/j.bbrc.2011.05.065. Epub 2011 May 17. PMID:

21620800.

96: Kim RY, Hoffman AS, Itoh N, Ao Y, Spence R, Sofroniew MV, Voskuhl RR.

Astrocyte CCL2 sustains immune cell infiltration in chronic experimental

autoimmune encephalomyelitis. J Neuroimmunol. 2014 Sep 15;274(1-2):53-61. doi:

10.1016/j.jneuroim.2014.06.009. Epub 2014 Jun 24. PMID: 25005117; PMCID:

PMC4343306.

97: Klemens J, Ciurkiewicz M, Chludzinski E, Iseringhausen M, Klotz D, Pfankuche

VM, Ulrich R, Herder V, Puff C, Baumgärtner W, Beineke A. Neurotoxic potential

of reactive astrocytes in canine distemper demyelinating leukoencephalitis. Sci

Rep. 2019 Aug 12;9(1):11689. doi: 10.1038/s41598-019-48146-9. PMID: 31406213;

PMCID: PMC6690900.

98: König HG, Coughlan KS, Kinsella S, Breen BA, Prehn JH. The BCL-2 family

protein Bid is critical for pro-inflammatory signaling in astrocytes. Neurobiol

Dis. 2014 Oct;70:99-107. doi: 10.1016/j.nbd.2014.06.008. Epub 2014 Jun 21. PMID:

24956542.

99: König R, Stillfried M, Aperdannier P, Clarner T, Beyer C, Kipp M, Mey J.

Expression of retinoid X receptor β is induced in astrocytes during corpus

callosum demyelination. J Chem Neuroanat. 2012 Mar;43(2):120-32. doi:

10.1016/j.jchemneu.2012.01.002. Epub 2012 Jan 28. PMID: 22306550.

100: Kordula T, Rydel RE, Brigham EF, Horn F, Heinrich PC, Travis J. Oncostatin

M and the interleukin-6 and soluble interleukin-6 receptor complex regulate

alpha1-antichymotrypsin expression in human cortical astrocytes. J Biol Chem.

1998 Feb 13;273(7):4112-8. doi: 10.1074/jbc.273.7.4112. PMID: 9461605.

101: Kordula T, Bugno M, Rydel RE, Travis J. Mechanism of interleukin-1- and

tumor necrosis factor alpha-dependent regulation of the alpha 1-antichymotrypsin

gene in human astrocytes. J Neurosci. 2000 Oct 15;20(20):7510-6. doi:

10.1523/JNEUROSCI.20-20-07510.2000. PMID: 11027208; PMCID: PMC6772857.

102: Koyama Y, Kotani M, Sawamura T, Kuribayashi M, Konishi R, Michinaga S.

Different actions of endothelin-1 on chemokine production in rat cultured

astrocytes: reduction of CX3CL1/fractalkine and an increase in CCL2/MCP-1 and

CXCL1/CINC-1. J Neuroinflammation. 2013 Apr 30;10:51. doi:

10.1186/1742-2094-10-51. PMID: 23627909; PMCID: PMC3675376.

103: Ko CY, Chang LH, Lee YC, Sterneck E, Cheng CP, Chen SH, Huang AM, Tseng JT,

Wang JM. CCAAT/enhancer binding protein delta (CEBPD) elevating PTX3 expression

inhibits macrophage-mediated phagocytosis of dying neuron cells. Neurobiol

Aging. 2012 Feb;33(2):422.e11-25. doi: 10.1016/j.neurobiolaging.2010.09.017.

Epub 2010 Nov 26. PMID: 21112127; PMCID: PMC6309870.

104: Krasovska V, Doering LC. Regulation of IL-6 Secretion by Astrocytes via

TLR4 in the Fragile X Mouse Model. Front Mol Neurosci. 2018 Aug 3;11:272. doi:

10.3389/fnmol.2018.00272. PMID: 30123107; PMCID: PMC6085486.

105: Krasowska-Zoladek A, Banaszewska M, Kraszpulski M, Konat GW. Kinetics of

inflammatory response of astrocytes induced by TLR 3 and TLR4 ligation. J

Neurosci Res. 2007 Jan;85(1):205-12. doi: 10.1002/jnr.21088. PMID: 17061254.

106: Kuhlow CJ, Krady JK, Basu A, Levison SW. Astrocytic ceruloplasmin

expression, which is induced by IL-1beta and by traumatic brain injury,

increases in the absence of the IL-1 type 1 receptor. Glia. 2003

Oct;44(1):76-84. doi: 10.1002/glia.10273. PMID: 12951659.

107: Kutsuno Y, Hirashima R, Sakamoto M, Ushikubo H, Michimae H, Itoh T, Tukey

RH, Fujiwara R. Expression of UDP-Glucuronosyltransferase 1 (UGT1) and

Glucuronidation Activity toward Endogenous Substances in Humanized UGT1 Mouse

Brain. Drug Metab Dispos. 2015 Jul;43(7):1071-6. doi: 10.1124/dmd.115.063719.

Epub 2015 May 7. PMID: 25953521; PMCID: PMC4468441.

108: Lagos-Cabré R, Alvarez A, Kong M, Burgos-Bravo F, Cárdenas A, Rojas-

Mancilla E, Pérez-Nuñez R, Herrera-Molina R, Rojas F, Schneider P, Herrera-

Marschitz M, Quest AFG, van Zundert B, Leyton L. α<sub>V</sub>β<sub>3</sub>

Integrin regulates astrocyte reactivity. J Neuroinflammation. 2017 Sep

29;14(1):194. doi: 10.1186/s12974-017-0968-5. PMID: 28962574; PMCID: PMC5622429.

109: Lananna BV, Nadarajah CJ, Izumo M, Cedeño MR, Xiong DD, Dimitry J, Tso CF,

McKee CA, Griffin P, Sheehan PW, Haspel JA, Barres BA, Liddelow SA, Takahashi

JS, Karatsoreos IN, Musiek ES. Cell-Autonomous Regulation of Astrocyte

Activation by the Circadian Clock Protein BMAL1. Cell Rep. 2018 Oct

2;25(1):1-9.e5. doi: 10.1016/j.celrep.2018.09.015. PMID: 30282019; PMCID:

PMC6221830.

110: Lapp DW, Zhang SS, Barnstable CJ. Stat3 mediates LIF-induced protection of

astrocytes against toxic ROS by upregulating the UPC2 mRNA pool. Glia. 2014

Feb;62(2):159-70. doi: 10.1002/glia.22594. Epub 2013 Dec 5. PMID: 24307565.

111: Laug D, Huang TW, Huerta NAB, Huang AY, Sardar D, Ortiz-Guzman J, Carlson

JC, Arenkiel BR, Kuo CT, Mohila CA, Glasgow SM, Lee HK, Deneen B. Nuclear factor

I-A regulates diverse reactive astrocyte responses after CNS injury. J Clin

Invest. 2019 Oct 1;129(10):4408-4418. doi: 10.1172/JCI127492. PMID: 31498149;

PMCID: PMC6763246.

112: Lee GA, Lai YG, Chen RJ, Liao NS. Interleukin 15 activates Akt to protect

astrocytes from oxygen glucose deprivation-induced cell death. Cytokine. 2017

Apr;92:68-74. doi: 10.1016/j.cyto.2017.01.010. Epub 2017 Jan 18. PMID: 28110119.

113: Lee JH, Park SM, Kim OS, Lee CS, Woo JH, Park SJ, Joe EH, Jou I.

Differential SUMOylation of LXRalpha and LXRbeta mediates transrepression of

STAT1 inflammatory signaling in IFN-gamma-stimulated brain astrocytes. Mol Cell.

2009 Sep 24;35(6):806-17. doi: 10.1016/j.molcel.2009.07.021. PMID: 19782030.

114: Lee JH, Han JH, Kim H, Park SM, Joe EH, Jou I. Parkinson's disease-

associated LRRK2-G2019S mutant acts through regulation of SERCA activity to

control ER stress in astrocytes. Acta Neuropathol Commun. 2019 May 2;7(1):68.

doi: 10.1186/s40478-019-0716-4. PMID: 31046837; PMCID: PMC6498585.

115: Lee SJ, Drabik K, Van Wagoner NJ, Lee S, Choi C, Dong Y, Benveniste EN.

ICAM-1-induced expression of proinflammatory cytokines in astrocytes:

involvement of extracellular signal-regulated kinase and p38 mitogen-activated

protein kinase pathways. J Immunol. 2000 Oct 15;165(8):4658-66. doi:

10.4049/jimmunol.165.8.4658. PMID: 11035109.

116: Lee S, Park JY, Lee WH, Kim H, Park HC, Mori K, Suk K. Lipocalin-2 is an

autocrine mediator of reactive astrocytosis. J Neurosci. 2009 Jan

7;29(1):234-49. doi: 10.1523/JNEUROSCI.5273-08.2009. PMID: 19129400; PMCID:

PMC6664907.

117: Lee S, Kim JH, Kim JH, Seo JW, Han HS, Lee WH, Mori K, Nakao K, Barasch J,

Suk K. Lipocalin-2 Is a chemokine inducer in the central nervous system: role of

chemokine ligand 10 (CXCL10) in lipocalin-2-induced cell migration. J Biol Chem.

2011 Dec 23;286(51):43855-43870. doi: 10.1074/jbc.M111.299248. Epub 2011 Oct 26.

PMID: 22030398; PMCID: PMC3243551.

118: Lee SJ, Seo BR, Choi EJ, Koh JY. The role of reciprocal activation of cAbl

and Mst1 in the oxidative death of cultured astrocytes. Glia. 2014

Apr;62(4):639-48. doi: 10.1002/glia.22631. Epub 2014 Jan 24. PMID: 24464935.

119: Leng L, Zhuang K, Liu Z, Huang C, Gao Y, Chen G, Lin H, Hu Y, Wu D, Shi M,

Xie W, Sun H, Shao Z, Li H, Zhang K, Mo W, Huang TY, Xue M, Yuan Z, Zhang X, Bu

G, Xu H, Xu Q, Zhang J. Menin Deficiency Leads to Depressive-like Behaviors in

Mice by Modulating Astrocyte-Mediated Neuroinflammation. Neuron. 2018 Nov

7;100(3):551-563.e7. doi: 10.1016/j.neuron.2018.08.031. Epub 2018 Sep 13. PMID:

30220511.

120: Liddelow SA, Guttenplan KA, Clarke LE, Bennett FC, Bohlen CJ, Schirmer L,

Bennett ML, Münch AE, Chung WS, Peterson TC, Wilton DK, Frouin A, Napier BA,

Panicker N, Kumar M, Buckwalter MS, Rowitch DH, Dawson VL, Dawson TM, Stevens B,

Barres BA. Neurotoxic reactive astrocytes are induced by activated microglia.

Nature. 2017 Jan 26;541(7638):481-487. doi: 10.1038/nature21029. Epub 2017 Jan

18. PMID: 28099414; PMCID: PMC5404890.

121: Lin B, Xu Y, Zhang B, He Y, Yan Y, He MC. MEK inhibition reduces glial scar

formation and promotes the recovery of sensorimotor function in rats following

spinal cord injury. Exp Ther Med. 2014 Jan;7(1):66-72. doi:

10.3892/etm.2013.1371. Epub 2013 Oct 29. PMID: 24348766; PMCID: PMC3861407.

122: Lin SX, Lisi L, Dello Russo C, Polak PE, Sharp A, Weinberg G, Kalinin S,

Feinstein DL. The anti-inflammatory effects of dimethyl fumarate in astrocytes

involve glutathione and haem oxygenase-1. ASN Neuro. 2011 Apr 7;3(2):e00055.

doi: 10.1042/AN20100033. PMID: 21382015; PMCID: PMC3072764.

123: Lipfert J, Ödemis V, Engele J. Grk2 is an essential regulator of CXCR7

signalling in astrocytes. Cell Mol Neurobiol. 2013 Jan;33(1):111-8. doi:

10.1007/s10571-012-9876-5. Epub 2012 Sep 2. PMID: 22940879.

124: Litvinchuk A, Wan YW, Swartzlander DB, Chen F, Cole A, Propson NE, Wang Q,

Zhang B, Liu Z, Zheng H. Complement C3aR Inactivation Attenuates Tau Pathology

and Reverses an Immune Network Deregulated in Tauopathy Models and Alzheimer's

Disease. Neuron. 2018 Dec 19;100(6):1337-1353.e5. doi:

10.1016/j.neuron.2018.10.031. Epub 2018 Nov 8. PMID: 30415998; PMCID:

PMC6309202.

125: Liu Y, Li Y, Zhan M, Liu Y, Li Z, Li J, Cheng G, Teng G, Lu L. Astrocytic

cytochrome P450 4A/20-hydroxyeicosatetraenoic acid contributes to angiogenesis

in the experimental ischemic stroke. Brain Res. 2019 Apr 1;1708:160-170. doi:

10.1016/j.brainres.2018.12.023. Epub 2018 Dec 17. PMID: 30571981.

126: Liu Z, Li Y, Cui Y, Roberts C, Lu M, Wilhelmsson U, Pekny M, Chopp M.

Beneficial effects of gfap/vimentin reactive astrocytes for axonal remodeling

and motor behavioral recovery in mice after stroke. Glia. 2014

Dec;62(12):2022-33. doi: 10.1002/glia.22723. Epub 2014 Jul 15. PMID: 25043249;

PMCID: PMC4307923.

127: Li S, Wang L, Berman MA, Zhang Y, Dorf ME. RNAi screen in mouse astrocytes

identifies phosphatases that regulate NF-kappaB signaling. Mol Cell. 2006 Nov

17;24(4):497-509. doi: 10.1016/j.molcel.2006.10.015. PMID: 17188031; PMCID:

PMC2572259.

128: Li Y, Xia Y, Wang Y, Mao L, Gao Y, He Q, Huang M, Chen S, Hu B. Sonic

hedgehog (Shh) regulates the expression of angiogenic growth factors in oxygen-

glucose-deprived astrocytes by mediating the nuclear receptor NR2F2. Mol

Neurobiol. 2013 Jun;47(3):967-75. doi: 10.1007/s12035-013-8395-9. Epub 2013 Feb

3. PMID: 23378030.

129: Li ZQ, Yan ZY, Lan FJ, Dong YQ, Xiong Y. Suppression of NLRP3 inflammasome

attenuates stress-induced depression-like behavior in NLGN3-deficient mice.

Biochem Biophys Res Commun. 2018 Jul 2;501(4):933-940. doi:

10.1016/j.bbrc.2018.05.085. Epub 2018 May 21. PMID: 29775613.

130: Loeffler S, Fayard B, Weis J, Weissenberger J. Interleukin-6 induces

transcriptional activation of vascular endothelial growth factor (VEGF) in

astrocytes in vivo and regulates VEGF promoter activity in glioblastoma cells

via direct interaction between STAT3 and Sp1. Int J Cancer. 2005 Jun

10;115(2):202-13. doi: 10.1002/ijc.20871. PMID: 15688401.

131: Luo J, Wu X, Liu H, Cui W, Guo W, Guo K, Guo H, Tao K, Li F, Shi Y, Feng D,

Yan H, Gao G, Qu Y. Antagonism of Protease-Activated Receptor 4 Protects Against

Traumatic Brain Injury by Suppressing Neuroinflammation via Inhibition of

Tab2/NF-κB Signaling. Neurosci Bull. 2021 Feb;37(2):242-254. doi:

10.1007/s12264-020-00601-8. Epub 2020 Oct 27. PMID: 33111257; PMCID: PMC7870748.

132: Lutgen V, Narasipura SD, Sharma A, Min S, Al-Harthi L. β-Catenin signaling

positively regulates glutamate uptake and metabolism in astrocytes. J

Neuroinflammation. 2016 Sep 10;13(1):242. doi: 10.1186/s12974-016-0691-7. PMID:

27612942; PMCID: PMC5018172.

133: Mamik MK, Banerjee S, Walseth TF, Hirte R, Tang L, Borgmann K, Ghorpade A.

HIV-1 and IL-1β regulate astrocytic CD38 through mitogen-activated protein

kinases and nuclear factor-κB signaling mechanisms. J Neuroinflammation. 2011

Oct 25;8:145. doi: 10.1186/1742-2094-8-145. PMID: 22027397; PMCID: PMC3247131.

134: Mayo L, Trauger SA, Blain M, Nadeau M, Patel B, Alvarez JI, Mascanfroni ID,

Yeste A, Kivisäkk P, Kallas K, Ellezam B, Bakshi R, Prat A, Antel JP, Weiner HL,

Quintana FJ. Regulation of astrocyte activation by glycolipids drives chronic

CNS inflammation. Nat Med. 2014 Oct;20(10):1147-56. doi: 10.1038/nm.3681. Epub

2014 Sep 14. PMID: 25216636; PMCID: PMC4255949.

135: Mayo L, Cunha AP, Madi A, Beynon V, Yang Z, Alvarez JI, Prat A, Sobel RA,

Kobzik L, Lassmann H, Quintana FJ, Weiner HL. IL-10-dependent Tr1 cells

attenuate astrocyte activation and ameliorate chronic central nervous system

inflammation. Brain. 2016 Jul;139(Pt 7):1939-57. doi: 10.1093/brain/aww113. Epub

2016 May 31. PMID: 27246324; PMCID: PMC4939696.

136: Mazumder AG, Patial V, Singh D. Mycophenolate mofetil contributes to

downregulation of the hippocampal interleukin type 2 and 1β mediated

PI3K/AKT/mTOR pathway hyperactivation and attenuates neurobehavioral

comorbidities in a rat model of temporal lobe epilepsy. Brain Behav Immun. 2019

Jan;75:84-93. doi: 10.1016/j.bbi.2018.09.020. Epub 2018 Sep 20. PMID: 30243822.

137: Ma K, Ding X, Song Q, Han Z, Yao H, Ding J, Hu G. Lactate enhances

Arc/arg3.1 expression through hydroxycarboxylic acid receptor 1-β-arrestin2

pathway in astrocytes. Neuropharmacology. 2020 Jul;171:108084. doi:

10.1016/j.neuropharm.2020.108084. Epub 2020 Apr 12. PMID: 32294462.

138: Mele T, Jurič DM. Metrifonate, like acetylcholine, up-regulates

neurotrophic activity of cultured rat astrocytes. Pharmacol Rep. 2014

Aug;66(4):618-23. doi: 10.1016/j.pharep.2014.02.025. Epub 2014 Apr 26. PMID:

24948063.

139: Miao Q, Ge M, Huang L. Up-regulation of GBP2 is Associated with Neuronal

Apoptosis in Rat Brain Cortex Following Traumatic Brain Injury. Neurochem Res.

2017 May;42(5):1515-1523. doi: 10.1007/s11064-017-2208-x. Epub 2017 Feb 27.

PMID: 28239766.

140: Michinaga S, Ishida A, Takeuchi R, Koyama Y. Endothelin-1 stimulates cyclin

D1 expression in rat cultured astrocytes via activation of Sp1. Neurochem Int.

2013 Jul;63(1):25-34. doi: 10.1016/j.neuint.2013.04.004. Epub 2013 Apr 22. PMID:

23619396.

141: Moidunny S, Matos M, Wesseling E, Banerjee S, Volsky DJ, Cunha RA,

Agostinho P, Boddeke HW, Roy S. Oncostatin M promotes excitotoxicity by

inhibiting glutamate uptake in astrocytes: implications in HIV-associated

neurotoxicity. J Neuroinflammation. 2016 Jun 10;13(1):144. doi:

10.1186/s12974-016-0613-8. PMID: 27287400; PMCID: PMC4903004.

142: Navarrete M, Araque A. Endocannabinoids mediate neuron-astrocyte

communication. Neuron. 2008 Mar 27;57(6):883-93. doi:

10.1016/j.neuron.2008.01.029. PMID: 18367089.

143: Neal ML, Boyle AM, Budge KM, Safadi FF, Richardson JR. The glycoprotein

GPNMB attenuates astrocyte inflammatory responses through the CD44 receptor. J

Neuroinflammation. 2018 Mar 8;15(1):73. doi: 10.1186/s12974-018-1100-1. PMID:

29519253; PMCID: PMC5842560.

144: Neal M, Luo J, Harischandra DS, Gordon R, Sarkar S, Jin H, Anantharam V,

Désaubry L, Kanthasamy A, Kanthasamy A. Prokineticin-2 promotes chemotaxis and

alternative A2 reactivity of astrocytes. Glia. 2018 Oct;66(10):2137-2157. doi:

10.1002/glia.23467. Epub 2018 Sep 12. PMID: 30277602; PMCID: PMC6240381.

145: Neumann C, Garreis F, Paulsen F, Hammer CM, Birke MT, Scholz M. Osteopontin

is induced by TGF-β2 and regulates metabolic cell activity in cultured human

optic nerve head astrocytes. PLoS One. 2014 Apr 9;9(4):e92762. doi:

10.1371/journal.pone.0092762. PMID: 24718314; PMCID: PMC3981660.

146: Nicchia GP, Srinivas M, Li W, Brosnan CF, Frigeri A, Spray DC. New possible

roles for aquaporin-4 in astrocytes: cell cytoskeleton and functional

relationship with connexin43. FASEB J. 2005 Oct;19(12):1674-6. doi:

10.1096/fj.04-3281fje. Epub 2005 Aug 15. PMID: 16103109.

147: Niego B, Freeman R, Puschmann TB, Turnley AM, Medcalf RL. t-PA-specific

modulation of a human blood-brain barrier model involves plasmin-mediated

activation of the Rho kinase pathway in astrocytes. Blood. 2012 May

17;119(20):4752-61. doi: 10.1182/blood-2011-07-369512. Epub 2012 Jan 19. PMID:

22262761.

148: Oeckl P, Lattke M, Wirth T, Baumann B, Ferger B. Astrocyte-specific IKK2

activation in mice is sufficient to induce neuroinflammation but does not

increase susceptibility to MPTP. Neurobiol Dis. 2012 Dec;48(3):481-7. doi:

10.1016/j.nbd.2012.06.010. Epub 2012 Jun 30. PMID: 22750522.

149: Ogier C, Creidy R, Boucraut J, Soloway PD, Khrestchatisky M, Rivera S.

Astrocyte reactivity to Fas activation is attenuated in TIMP-1 deficient mice,

an in vitro study. BMC Neurosci. 2005 Nov 29;6:68. doi: 10.1186/1471-2202-6-68.

PMID: 16316466; PMCID: PMC1325973.

150: Ohta K, Kuno S, Inoue S, Ikeda E, Fujinami A, Ohta M. The effect of

dopamine agonists: the expression of GDNF, NGF, and BDNF in cultured mouse

astrocytes. J Neurol Sci. 2010 Apr 15;291(1-2):12-6. doi:

10.1016/j.jns.2010.01.013. Epub 2010 Feb 2. PMID: 20129627.

151: Oh SM, Chang MY, Song JJ, Rhee YH, Joe EH, Lee HS, Yi SH, Lee SH. Combined

Nurr1 and Foxa2 roles in the therapy of Parkinson's disease. EMBO Mol Med. 2015

May;7(5):510-25. doi: 10.15252/emmm.201404610. Erratum in: EMBO Mol Med.

2016;8(2):171. PMID: 25759364; PMCID: PMC4492814.

152: Ouali Alami N, Schurr C, Olde Heuvel F, Tang L, Li Q, Tasdogan A, Kimbara

A, Nettekoven M, Ottaviani G, Raposo C, Röver S, Rogers-Evans M, Rothenhäusler

B, Ullmer C, Fingerle J, Grether U, Knuesel I, Boeckers TM, Ludolph A, Wirth T,

Roselli F, Baumann B. NF-κB activation in astrocytes drives a stage-specific

beneficial neuroimmunological response in ALS. EMBO J. 2018 Aug

15;37(16):e98697. doi: 10.15252/embj.201798697. Epub 2018 Jun 6. PMID: 29875132;

PMCID: PMC6092622.

153: Pacey LK, Guan S, Tharmalingam S, Thomsen C, Hampson DR. Persistent

astrocyte activation in the fragile X mouse cerebellum. Brain Behav. 2015 Sep

25;5(10):e00400. doi: 10.1002/brb3.400. PMID: 26516618; PMCID: PMC4614053.

154: Pannell M, Economopoulos V, Wilson TC, Kersemans V, Isenegger PG, Larkin

JR, Smart S, Gilchrist S, Gouverneur V, Sibson NR. Imaging of translocator

protein upregulation is selective for pro-inflammatory polarized astrocytes and

microglia. Glia. 2020 Feb;68(2):280-297. doi: 10.1002/glia.23716. Epub 2019 Sep

3. PMID: 31479168; PMCID: PMC6916298.

155: Pan LN, Zhu W, Li Y, Xu XL, Guo LJ, Lu Q, Wang J. Astrocytic Toll-like

receptor 3 is associated with ischemic preconditioning-induced protection

against brain ischemia in rodents. PLoS One. 2014 Jun 10;9(6):e99526. doi:

10.1371/journal.pone.0099526. PMID: 24914679; PMCID: PMC4051824.

156: Park JH, Riew TR, Shin YJ, Park JM, Cho JM, Lee MY. Induction of Krüppel-

like factor 4 expression in reactive astrocytes following ischemic injury in

vitro and in vivo. Histochem Cell Biol. 2014 Jan;141(1):33-42. doi:

10.1007/s00418-013-1134-5. Epub 2013 Aug 10. PMID: 23934449.

157: Patel BN, Dunn RJ, Jeong SY, Zhu Q, Julien JP, David S. Ceruloplasmin

regulates iron levels in the CNS and prevents free radical injury. J Neurosci.

2002 Aug 1;22(15):6578-86. doi: 10.1523/JNEUROSCI.22-15-06578.2002. PMID:

12151537; PMCID: PMC6758125.

158: Patel H, McIntire J, Ryan S, Dunah A, Loring R. Anti-inflammatory effects

of astroglial α7 nicotinic acetylcholine receptors are mediated by inhibition of

the NF-κB pathway and activation of the Nrf2 pathway. J Neuroinflammation. 2017

Sep 26;14(1):192. doi: 10.1186/s12974-017-0967-6. PMID: 28950908; PMCID:

PMC5615458.

159: Predebon J. Perceived size of familiar objects and the theory of off-sized

perceptions. Percept Psychophys. 1994 Aug;56(2):238-47. doi: 10.3758/bf03213902.

PMID: 7971124.

160: Qadri F, Wolf A, Waldmann T, Rascher W, Unger T. Sensitivity of

hypothalamic paraventricular nucleus to C- and N-terminal angiotensin fragments:

vasopressin release and drinking. J Neuroendocrinol. 1998 Apr;10(4):275-81.

PMID: 9630397.

161: Qian D, Li L, Rong Y, Liu W, Wang Q, Zhou Z, Gu C, Huang Y, Zhao X, Chen J,

Fan J, Yin G. Blocking Notch signal pathway suppresses the activation of

neurotoxic A1 astrocytes after spinal cord injury. Cell Cycle. 2019

Nov;18(21):3010-3029. doi: 10.1080/15384101.2019.1667189. Epub 2019 Sep 18.

PMID: 31530090; PMCID: PMC6791691.

162: Qiao C, Yin N, Gu HY, Zhu JL, Ding JH, Lu M, Hu G. Atp13a2 Deficiency

Aggravates Astrocyte-Mediated Neuroinflammation via NLRP3 Inflammasome

Activation. CNS Neurosci Ther. 2016 Jun;22(6):451-60. doi: 10.1111/cns.12514.

Epub 2016 Feb 5. PMID: 26848562; PMCID: PMC6492810.

163: Qin H, Niyongere SA, Lee SJ, Baker BJ, Benveniste EN. Expression and

functional significance of SOCS-1 and SOCS-3 in astrocytes. J Immunol. 2008 Sep

1;181(5):3167-76. doi: 10.4049/jimmunol.181.5.3167. PMID: 18713987; PMCID:

PMC2836124.

164: Retamal MA, Froger N, Palacios-Prado N, Ezan P, Sáez PJ, Sáez JC, Giaume C.

Cx43 hemichannels and gap junction channels in astrocytes are regulated

oppositely by proinflammatory cytokines released from activated microglia. J

Neurosci. 2007 Dec 12;27(50):13781-92. doi: 10.1523/JNEUROSCI.2042-07.2007.

PMID: 18077690; PMCID: PMC6673621.

165: Rius J, Guma M, Schachtrup C, Akassoglou K, Zinkernagel AS, Nizet V,

Johnson RS, Haddad GG, Karin M. NF-kappaB links innate immunity to the hypoxic

response through transcriptional regulation of HIF-1alpha. Nature. 2008 Jun

5;453(7196):807-11. doi: 10.1038/nature06905. Epub 2008 Apr 23. PMID: 18432192;

PMCID: PMC2669289.

166: Rivieccio MA, John GR, Song X, Suh HS, Zhao Y, Lee SC, Brosnan CF. The

cytokine IL-1beta activates IFN response factor 3 in human fetal astrocytes in

culture. J Immunol. 2005 Mar 15;174(6):3719-26. doi:

10.4049/jimmunol.174.6.3719. PMID: 15749911.

167: Rodriguez-Grande B, Swana M, Nguyen L, Englezou P, Maysami S, Allan SM,

Rothwell NJ, Garlanda C, Denes A, Pinteaux E. The acute-phase protein PTX3 is an

essential mediator of glial scar formation and resolution of brain edema after

ischemic injury. J Cereb Blood Flow Metab. 2014 Mar;34(3):480-8. doi:

10.1038/jcbfm.2013.224. Epub 2013 Dec 18. PMID: 24346689; PMCID: PMC3948128.

168: Roque C, Mendes-Oliveira J, Baltazar G. G protein-coupled estrogen receptor

activates cell type-specific signaling pathways in cortical cultures: relevance

to the selective loss of astrocytes. J Neurochem. 2019 Apr;149(1):27-40. doi:

10.1111/jnc.14648. Epub 2019 Jan 28. PMID: 30570746.

169: Rose CR, Blum R, Pichler B, Lepier A, Kafitz KW, Konnerth A. Truncated

TrkB-T1 mediates neurotrophin-evoked calcium signalling in glia cells. Nature.

2003 Nov 6;426(6962):74-8. doi: 10.1038/nature01983. PMID: 14603320.

170: Rothhammer V, Mascanfroni ID, Bunse L, Takenaka MC, Kenison JE, Mayo L,

Chao CC, Patel B, Yan R, Blain M, Alvarez JI, Kébir H, Anandasabapathy N,

Izquierdo G, Jung S, Obholzer N, Pochet N, Clish CB, Prinz M, Prat A, Antel J,

Quintana FJ. Type I interferons and microbial metabolites of tryptophan modulate

astrocyte activity and central nervous system inflammation via the aryl

hydrocarbon receptor. Nat Med. 2016 Jun;22(6):586-97. doi: 10.1038/nm.4106. Epub

2016 May 9. PMID: 27158906; PMCID: PMC4899206.

171: Rothhammer V, Kenison JE, Tjon E, Takenaka MC, de Lima KA, Borucki DM, Chao

CC, Wilz A, Blain M, Healy L, Antel J, Quintana FJ. Sphingosine 1-phosphate

receptor modulation suppresses pathogenic astrocyte activation and chronic

progressive CNS inflammation. Proc Natl Acad Sci U S A. 2017 Feb

21;114(8):2012-2017. doi: 10.1073/pnas.1615413114. Epub 2017 Feb 6. PMID:

28167760; PMCID: PMC5338419.

172: Rothhammer V, Borucki DM, Tjon EC, Takenaka MC, Chao CC, Ardura-Fabregat A,

de Lima KA, Gutiérrez-Vázquez C, Hewson P, Staszewski O, Blain M, Healy L,

Neziraj T, Borio M, Wheeler M, Dragin LL, Laplaud DA, Antel J, Alvarez JI, Prinz

M, Quintana FJ. Microglial control of astrocytes in response to microbial

metabolites. Nature. 2018 May;557(7707):724-728. doi: 10.1038/s41586-018-0119-x.

Epub 2018 May 16. PMID: 29769726; PMCID: PMC6422159.

173: Saijo K, Winner B, Carson CT, Collier JG, Boyer L, Rosenfeld MG, Gage FH,

Glass CK. A Nurr1/CoREST pathway in microglia and astrocytes protects

dopaminergic neurons from inflammation-induced death. Cell. 2009 Apr

3;137(1):47-59. doi: 10.1016/j.cell.2009.01.038. PMID: 19345186; PMCID:

PMC2754279.

174: Santos R, Vadodaria KC, Jaeger BN, Mei A, Lefcochilos-Fogelquist S, Mendes

APD, Erikson G, Shokhirev M, Randolph-Moore L, Fredlender C, Dave S, Oefner R,

Fitzpatrick C, Pena M, Barron JJ, Ku M, Denli AM, Kerman BE, Charnay P, Kelsoe

JR, Marchetto MC, Gage FH. Differentiation of Inflammation-Responsive Astrocytes

from Glial Progenitors Generated from Human Induced Pluripotent Stem Cells. Stem

Cell Reports. 2017 Jun 6;8(6):1757-1769. doi: 10.1016/j.stemcr.2017.05.011.

PMID: 28591655; PMCID: PMC5470172.

175: Sanyal A, DeAndrade MP, Novis HS, Lin S, Chang J, Lengacher N, Tomlinson

JJ, Tansey MG, LaVoie MJ. Lysosome and Inflammatory Defects in GBA1-Mutant

Astrocytes Are Normalized by LRRK2 Inhibition. Mov Disord. 2020

May;35(5):760-773. doi: 10.1002/mds.27994. Epub 2020 Feb 8. PMID: 32034799;

PMCID: PMC8167931.

176: Schachtrup C, Ryu JK, Helmrick MJ, Vagena E, Galanakis DK, Degen JL,

Margolis RU, Akassoglou K. Fibrinogen triggers astrocyte scar formation by

promoting the availability of active TGF-beta after vascular damage. J Neurosci.

2010 Apr 28;30(17):5843-54. doi: 10.1523/JNEUROSCI.0137-10.2010. PMID: 20427645;

PMCID: PMC2871011.

177: Schneider L, Pellegatta S, Favaro R, Pisati F, Roncaglia P, Testa G,

Nicolis SK, Finocchiaro G, d'Adda di Fagagna F. DNA damage in mammalian neural

stem cells leads to astrocytic differentiation mediated by BMP2 signaling

through JAK-STAT. Stem Cell Reports. 2013 Jul 25;1(2):123-38. doi:

10.1016/j.stemcr.2013.06.004. PMID: 24052948; PMCID: PMC3757751.

178: Schwarz L, Vollmer G, Richter-Landsberg C. The Small Heat Shock Protein

HSP25/27 (HspB1) Is Abundant in Cultured Astrocytes and Associated with

Astrocytic Pathology in Progressive Supranuclear Palsy and Corticobasal

Degeneration. Int J Cell Biol. 2010;2010:717520. doi: 10.1155/2010/717520. Epub

2010 Jan 27. PMID: 20150973; PMCID: PMC2817856.

179: Shao W, Zhang SZ, Tang M, Zhang XH, Zhou Z, Yin YQ, Zhou QB, Huang YY, Liu

YJ, Wawrousek E, Chen T, Li SB, Xu M, Zhou JN, Hu G, Zhou JW. Suppression of

neuroinflammation by astrocytic dopamine D2 receptors via αB-crystallin. Nature.

2013 Feb 7;494(7435):90-4. doi: 10.1038/nature11748. Epub 2012 Dec 16. PMID:

23242137.

180: Sharma R, Fischer MT, Bauer J, Felts PA, Smith KJ, Misu T, Fujihara K,

Bradl M, Lassmann H. Inflammation induced by innate immunity in the central

nervous system leads to primary astrocyte dysfunction followed by demyelination.

Acta Neuropathol. 2010 Aug;120(2):223-36. doi: 10.1007/s00401-010-0704-z. Epub

2010 Jun 8. PMID: 20532539; PMCID: PMC2892605.

181: Sheng WS, Hu S, Nettles AR, Lokensgard JR, Vercellotti GM, Rock RB. Hemin

inhibits NO production by IL-1β-stimulated human astrocytes through induction of

heme oxygenase-1 and reduction of p38 MAPK activation. J Neuroinflammation. 2010

Sep 7;7:51. doi: 10.1186/1742-2094-7-51. PMID: 20822529; PMCID: PMC2949627.

182: Shin SY, Song H, Kim CG, Choi YK, Lee KS, Lee SJ, Lee HJ, Lim Y, Lee YH.

Egr-1 is necessary for fibroblast growth factor-2-induced transcriptional

activation of the glial cell line-derived neurotrophic factor in murine

astrocytes. J Biol Chem. 2009 Oct 30;284(44):30583-93. doi:

10.1074/jbc.M109.010678. Epub 2009 Aug 31. PMID: 19721135; PMCID: PMC2781613.

183: Shrikant P, Chung IY, Ballestas ME, Benveniste EN. Regulation of

intercellular adhesion molecule-1 gene expression by tumor necrosis factor-

alpha, interleukin-1 beta, and interferon-gamma in astrocytes. J Neuroimmunol.

1994 May;51(2):209-20. doi: 10.1016/0165-5728(94)90083-3. PMID: 7910170.

184: Sobue A, Ito N, Nagai T, Shan W, Hada K, Nakajima A, Murakami Y, Mouri A,

Yamamoto Y, Nabeshima T, Saito K, Yamada K. Astroglial major histocompatibility

complex class I following immune activation leads to behavioral and

neuropathological changes. Glia. 2018 May;66(5):1034-1052. doi:

10.1002/glia.23299. Epub 2018 Jan 30. PMID: 29380419.

185: Song S, Miranda CJ, Braun L, Meyer K, Frakes AE, Ferraiuolo L, Likhite S,

Bevan AK, Foust KD, McConnell MJ, Walker CM, Kaspar BK. Major histocompatibility

complex class I molecules protect motor neurons from astrocyte-induced toxicity

in amyotrophic lateral sclerosis. Nat Med. 2016 Apr;22(4):397-403. doi:

10.1038/nm.4052. Epub 2016 Feb 29. PMID: 26928464; PMCID: PMC4823173.

186: Sriram K, Benkovic SA, Hebert MA, Miller DB, O'Callaghan JP. Induction of

gp130-related cytokines and activation of JAK2/STAT3 pathway in astrocytes

precedes up-regulation of glial fibrillary acidic protein in the

1-methyl-4-phenyl-1,2,3,6-tetrahydropyridine model of neurodegeneration: key

signaling pathway for astrogliosis in vivo? J Biol Chem. 2004 May

7;279(19):19936-47. doi: 10.1074/jbc.M309304200. Epub 2004 Mar 2. PMID:

14996842.

187: Sun L, Shen R, Agnihotri SK, Chen Y, Huang Z, Büeler H. Lack of PINK1

alters glia innate immune responses and enhances inflammation-induced, nitric

oxide-mediated neuron death. Sci Rep. 2018 Jan 10;8(1):383. doi:

10.1038/s41598-017-18786-w. PMID: 29321620; PMCID: PMC5762685.

188: Takarada-Iemata M, Kezuka D, Takeichi T, Ikawa M, Hattori T, Kitao Y, Hori

O. Deletion of N-myc downstream-regulated gene 2 attenuates reactive

astrogliosis and inflammatory response in a mouse model of cortical stab injury.

J Neurochem. 2014 Aug;130(3):374-87. doi: 10.1111/jnc.12729. Epub 2014 Apr 25.

PMID: 24697507.

189: Tarassishin L, Loudig O, Bauman A, Shafit-Zagardo B, Suh HS, Lee SC.

Interferon regulatory factor 3 inhibits astrocyte inflammatory gene expression

through suppression of the proinflammatory miR-155 and miR-155*. Glia. 2011

Dec;59(12):1911-22. doi: 10.1002/glia.21233. PMID: 22170100; PMCID: PMC3241213.

190: Tarassishin L, Suh HS, Lee SC. LPS and IL-1 differentially activate mouse

and human astrocytes: role of CD14. Glia. 2014 Jun;62(6):999-1013. doi:

10.1002/glia.22657. Epub 2014 Mar 21. PMID: 24659539; PMCID: PMC4015139.

191: Tcw J, Wang M, Pimenova AA, Bowles KR, Hartley BJ, Lacin E, Machlovi SI,

Abdelaal R, Karch CM, Phatnani H, Slesinger PA, Zhang B, Goate AM, Brennand KJ.

An Efficient Platform for Astrocyte Differentiation from Human Induced

Pluripotent Stem Cells. Stem Cell Reports. 2017 Aug 8;9(2):600-614. doi:

10.1016/j.stemcr.2017.06.018. Epub 2017 Jul 27. PMID: 28757165; PMCID:

PMC5550034.

192: Tjalkens RB, Liu X, Mohl B, Wright T, Moreno JA, Carbone DL, Safe S. The

peroxisome proliferator-activated receptor-gamma agonist

1,1-bis(3'-indolyl)-1-(p-trifluoromethylphenyl)methane suppresses manganese-

induced production of nitric oxide in astrocytes and inhibits apoptosis in

cocultured PC12 cells. J Neurosci Res. 2008 Feb 15;86(3):618-29. doi:

10.1002/jnr.21524. PMID: 18041089.

193: Tokuda E, Okawa E, Ono S. Dysregulation of intracellular copper trafficking

pathway in a mouse model of mutant copper/zinc superoxide dismutase-linked

familial amyotrophic lateral sclerosis. J Neurochem. 2009 Oct;111(1):181-91.

doi: 10.1111/j.1471-4159.2009.06310.x. Epub 2009 Jul 27. PMID: 19656261.

194: Tran MD, Neary JT. Purinergic signaling induces thrombospondin-1 expression

in astrocytes. Proc Natl Acad Sci U S A. 2006 Jun 13;103(24):9321-6. doi:

10.1073/pnas.0603146103. Epub 2006 Jun 5. PMID: 16754856; PMCID: PMC1482608.

195: Tyzack GE, Hall CE, Sibley CR, Cymes T, Forostyak S, Carlino G, Meyer IF,

Schiavo G, Zhang SC, Gibbons GM, Newcombe J, Patani R, Lakatos A. A

neuroprotective astrocyte state is induced by neuronal signal EphB1 but fails in

ALS models. Nat Commun. 2017 Oct 27;8(1):1164. doi: 10.1038/s41467-017-01283-z.

PMID: 29079839; PMCID: PMC5660125.

196: van Neerven S, Regen T, Wolf D, Nemes A, Johann S, Beyer C, Hanisch UK, Mey

J. Inflammatory chemokine release of astrocytes in vitro is reduced by all-trans

retinoic acid. J Neurochem. 2010 Sep 1;114(5):1511-26. doi:

10.1111/j.1471-4159.2010.06867.x. Epub 2010 Jun 16. PMID: 20557428.

197: van Neerven S, Nemes A, Imholz P, Regen T, Denecke B, Johann S, Beyer C,

Hanisch UK, Mey J. Inflammatory cytokine release of astrocytes in vitro is

reduced by all-trans retinoic acid. J Neuroimmunol. 2010 Dec 15;229(1-2):169-79.

doi: 10.1016/j.jneuroim.2010.08.005. Epub 2010 Sep 9. PMID: 20826012.

198: Villarreal A, Seoane R, González Torres A, Rosciszewski G, Angelo MF, Rossi

A, Barker PA, Ramos AJ. S100B protein activates a RAGE-dependent autocrine loop

in astrocytes: implications for its role in the propagation of reactive gliosis.

J Neurochem. 2014 Oct;131(2):190-205. doi: 10.1111/jnc.12790. Epub 2014 Jul 5.

PMID: 24923428.

199: Vodret S, Bortolussi G, Jašprová J, Vitek L, Muro AF. Inflammatory

signature of cerebellar neurodegeneration during neonatal hyperbilirubinemia in

Ugt1 <sup>-/-</sup> mouse model. J Neuroinflammation. 2017 Mar 24;14(1):64. doi:

10.1186/s12974-017-0838-1. PMID: 28340583; PMCID: PMC5366125.

200: Vom Berg J, Prokop S, Miller KR, Obst J, Kälin RE, Lopategui-Cabezas I,

Wegner A, Mair F, Schipke CG, Peters O, Winter Y, Becher B, Heppner FL.

Inhibition of IL-12/IL-23 signaling reduces Alzheimer's disease-like pathology

and cognitive decline. Nat Med. 2012 Dec;18(12):1812-9. doi: 10.1038/nm.2965.

Epub 2012 Nov 25. PMID: 23178247.

201: Wagner LK, Gilling KE, Schormann E, Kloetzel PM, Heppner FL, Krüger E,

Prokop S. Immunoproteasome deficiency alters microglial cytokine response and

improves cognitive deficits in Alzheimer's disease-like APPPS1 mice. Acta

Neuropathol Commun. 2017 Jun 24;5(1):52. doi: 10.1186/s40478-017-0453-5. PMID:

28646899; PMCID: PMC5483273.

202: Wang C, Zhang CJ, Martin BN, Bulek K, Kang Z, Zhao J, Bian G, Carman JA,

Gao J, Dongre A, Xue H, Miller SD, Qian Y, Hambardzumyan D, Hamilton T,

Ransohoff RM, Li X. IL-17 induced NOTCH1 activation in oligodendrocyte

progenitor cells enhances proliferation and inflammatory gene expression. Nat

Commun. 2017 May 31;8:15508. doi: 10.1038/ncomms15508. PMID: 28561022; PMCID:

PMC5460031.

203: Wang D, Wang S, Ji B, Zheng M. Spatiotemporal expression of FOXA1

correlates with reactive gliosis after spinal cord injury. Neuropeptides. 2017

Dec;66:36-44. doi: 10.1016/j.npep.2017.08.002. Epub 2017 Aug 18. PMID: 28844448.

204: Wang R, Yang B, Zhang D. Activation of interferon signaling pathways in

spinal cord astrocytes from an ALS mouse model. Glia. 2011 Jun;59(6):946-58.

doi: 10.1002/glia.21167. Epub 2011 Mar 28. PMID: 21446050; PMCID: PMC3077460.

205: Wang Y, Cheng X, He Q, Zheng Y, Kim DH, Whittemore SR, Cao QL. Astrocytes

from the contused spinal cord inhibit oligodendrocyte differentiation of adult

oligodendrocyte precursor cells by increasing the expression of bone

morphogenetic proteins. J Neurosci. 2011 Apr 20;31(16):6053-8. doi:

10.1523/JNEUROSCI.5524-09.2011. PMID: 21508230; PMCID: PMC3081104.

206: Warren KM, Reeves TM, Phillips LL. MT5-MMP, ADAM-10, and N-cadherin act in

concert to facilitate synapse reorganization after traumatic brain injury. J

Neurotrauma. 2012 Jul 1;29(10):1922-40. doi: 10.1089/neu.2012.2383. Epub 2012

May 14. PMID: 22489706; PMCID: PMC3390984.

207: Weidemann A, Krohne TU, Aguilar E, Kurihara T, Takeda N, Dorrell MI, Simon

MC, Haase VH, Friedlander M, Johnson RS. Astrocyte hypoxic response is essential

for pathological but not developmental angiogenesis of the retina. Glia. 2010

Aug;58(10):1177-85. doi: 10.1002/glia.20997. PMID: 20544853; PMCID: PMC2993327.

208: Wei T, Wang Y, Xu W, Liu Y, Chen H, Yu Z. KCa3.1 deficiency attenuates

neuroinflammation by regulating an astrocyte phenotype switch involving the

PI3K/AKT/GSK3β pathway. Neurobiol Dis. 2019 Dec;132:104588. doi:

10.1016/j.nbd.2019.104588. Epub 2019 Aug 27. PMID: 31470105.

209: Werkman I, Sikkema AH, Versluijs JB, Qin J, de Boer P, Baron W. TLR3

agonists induce fibronectin aggregation by activated astrocytes: a role of pro-

inflammatory cytokines and fibronectin splice variants. Sci Rep. 2020 Jan

17;10(1):532. doi: 10.1038/s41598-019-57069-4. PMID: 31953424; PMCID:

PMC6969115.

210: Wheeler MA, Clark IC, Tjon EC, Li Z, Zandee SEJ, Couturier CP, Watson BR,

Scalisi G, Alkwai S, Rothhammer V, Rotem A, Heyman JA, Thaploo S, Sanmarco LM,

Ragoussis J, Weitz DA, Petrecca K, Moffitt JR, Becher B, Antel JP, Prat A,

Quintana FJ. MAFG-driven astrocytes promote CNS inflammation. Nature. 2020

Feb;578(7796):593-599. doi: 10.1038/s41586-020-1999-0. Epub 2020 Feb 12. PMID:

32051591; PMCID: PMC8049843.

211: Wilczynska KM, Gopalan SM, Bugno M, Kasza A, Konik BS, Bryan L, Wright S,

Griswold-Prenner I, Kordula T. A novel mechanism of tissue inhibitor of

metalloproteinases-1 activation by interleukin-1 in primary human astrocytes. J

Biol Chem. 2006 Nov 17;281(46):34955-64. doi: 10.1074/jbc.M604616200. Epub 2006

Sep 29. PMID: 17012236.

212: Wilhelm CJ, Hashimoto JG, Roberts ML, Zhang X, Goeke CM, Bloom SH,

Guizzetti M. Plasminogen activator system homeostasis and its dysregulation by

ethanol in astrocyte cultures and the developing brain. Neuropharmacology. 2018

Aug;138:193-209. doi: 10.1016/j.neuropharm.2018.06.004. Epub 2018 Jun 6. PMID:

29885422; PMCID: PMC6310223.

213: Wilson JX, Dragan M. Sepsis inhibits recycling and glutamate-stimulated

export of ascorbate by astrocytes. Free Radic Biol Med. 2005 Oct 15;39(8):990-8.

doi: 10.1016/j.freeradbiomed.2005.05.020. PMID: 16198226.

214: Wu HQ, Pereira EF, Bruno JP, Pellicciari R, Albuquerque EX, Schwarcz R. The

astrocyte-derived alpha7 nicotinic receptor antagonist kynurenic acid controls

extracellular glutamate levels in the prefrontal cortex. J Mol Neurosci. 2010

Jan;40(1-2):204-10. doi: 10.1007/s12031-009-9235-2. Epub 2009 Aug 19. PMID:

19690987; PMCID: PMC3929341.

215: Xia M, Zhu Y. FOXO3a involvement in the release of TNF-α stimulated by ATP

in spinal cord astrocytes. J Mol Neurosci. 2013 Nov;51(3):792-804. doi:

10.1007/s12031-013-0067-8. Epub 2013 Jul 17. PMID: 23860688.

216: Xia M, Zhu Y. Fibronectin enhances spinal cord astrocyte proliferation by

elevating P2Y1 receptor expression. J Neurosci Res. 2014 Aug;92(8):1078-90. doi:

10.1002/jnr.23384. Epub 2014 Mar 31. PMID: 24687862.

217: Xue P, Chen L, Lu X, Zhang J, Bao G, Xu G, Sun Y, Guo X, Jiang J, Gu H, Cui

Z. Vimentin Promotes Astrocyte Activation After Chronic Constriction Injury. J

Mol Neurosci. 2017 Sep;63(1):91-99. doi: 10.1007/s12031-017-0961-6. Epub 2017

Aug 8. PMID: 28791619.

218: Xu J, Chavis JA, Racke MK, Drew PD. Peroxisome proliferator-activated

receptor-alpha and retinoid X receptor agonists inhibit inflammatory responses

of astrocytes. J Neuroimmunol. 2006 Jul;176(1-2):95-105. doi:

10.1016/j.jneuroim.2006.04.019. Epub 2006 Jun 9. PMID: 16764943.

219: Xu J, Zheng Y, Lv S, Kang J, Yu Y, Hou K, Li Y, Chi G. Lactate Promotes

Reactive Astrogliosis and Confers Axon Guidance Potential to Astrocytes under

Oxygen-Glucose Deprivation. Neuroscience. 2020 Aug 21;442:54-68. doi:

10.1016/j.neuroscience.2020.06.041. Epub 2020 Jul 4. PMID: 32634533.

220: Xu L, Wang L, Wen Z, Wu L, Jiang Y, Yang L, Xiao L, Xie Y, Ma M, Zhu W, Ye

R, Liu X. Caveolin-1 is a checkpoint regulator in hypoxia-induced astrocyte

apoptosis via Ras/Raf/ERK pathway. Am J Physiol Cell Physiol. 2016 Jun

1;310(11):C903-10. doi: 10.1152/ajpcell.00309.2015. Epub 2016 Mar 23. PMID:

27009876.

221: Yang X, Chen S, Shao Z, Li Y, Wu H, Li X, Mao L, Zhou Z, Bai L, Mei X, Liu

C. Apolipoprotein E Deficiency Exacerbates Spinal Cord Injury in Mice:

Inflammatory Response and Oxidative Stress Mediated by NF-κB Signaling Pathway.

Front Cell Neurosci. 2018 May 23;12:142. doi: 10.3389/fncel.2018.00142. PMID:

29875635; PMCID: PMC5974465.

222: Yan Y, Ding X, Li K, Ciric B, Wu S, Xu H, Gran B, Rostami A, Zhang GX. CNS-

specific therapy for ongoing EAE by silencing IL-17 pathway in astrocytes. Mol

Ther. 2012 Jul;20(7):1338-48. doi: 10.1038/mt.2012.12. Epub 2012 Mar 20. Erratum

in: Mol Ther. 2014 Dec;22(12):2155. PMID: 22434134; PMCID: PMC3392982.

223: Yao X, Jiang Q, Ding W, Yue P, Wang J, Zhao K, Zhang H. Interleukin 4

inhibits high mobility group box-1 protein-mediated NLRP3 inflammasome formation

by activating peroxisome proliferator-activated receptor-γ in astrocytes.

Biochem Biophys Res Commun. 2019 Feb 5;509(2):624-631. doi:

10.1016/j.bbrc.2018.11.145. Epub 2018 Dec 31. PMID: 30606476.

224: Yi M, Dou F, Lu Q, Yu Z, Chen H. Activation of the KCa3.1 channel

contributes to traumatic scratch injury-induced reactive astrogliosis through

the JNK/c-Jun signaling pathway. Neurosci Lett. 2016 Jun 15;624:62-71. doi:

10.1016/j.neulet.2016.05.004. Epub 2016 May 6. PMID: 27163196.

225: Yoo BK, Choi JW, Shin CY, Jeon SJ, Park SJ, Cheong JH, Han SY, Ryu JR, Song

MR, Ko KH. Activation of p38 MAPK induced peroxynitrite generation in LPS plus

IFN-gamma-stimulated rat primary astrocytes via activation of iNOS and NADPH

oxidase. Neurochem Int. 2008 May;52(6):1188-97. doi:

10.1016/j.neuint.2007.12.009. Epub 2007 Dec 27. PMID: 18289732.

226: Yueh MF, Chen S, Nguyen N, Tukey RH. Developmental onset of bilirubin-

induced neurotoxicity involves Toll-like receptor 2-dependent signaling in

humanized UDP-glucuronosyltransferase1 mice. J Biol Chem. 2014 Feb

21;289(8):4699-709. doi: 10.1074/jbc.M113.518613. Epub 2014 Jan 8. PMID:

24403077; PMCID: PMC3931032.

227: Yun JH, Park SJ, Jo A, Kang JL, Jou I, Park JS, Choi YH. Caveolin-1 is

involved in reactive oxygen species-induced SHP-2 activation in astrocytes. Exp

Mol Med. 2011 Dec 31;43(12):660-8. doi: 10.3858/emm.2011.43.12.075. PMID:

21918362; PMCID: PMC3256293.

228: Yu AL, Fuchshofer R, Birke M, Kampik A, Bloemendal H, Welge-Lüssen U.

Oxidative stress and TGF-beta2 increase heat shock protein 27 expression in

human optic nerve head astrocytes. Invest Ophthalmol Vis Sci. 2008

Dec;49(12):5403-11. doi: 10.1167/iovs.07-1478. Epub 2008 Jun 14. PMID: 18552392.

229: Yu Q, Zhao MW, Yang P. LncRNA UCA1 Suppresses the Inflammation Via

Modulating miR-203-Mediated Regulation of MEF2C/NF-κB Signaling Pathway in

Epilepsy. Neurochem Res. 2020 Apr;45(4):783-795. doi:

10.1007/s11064-019-02952-9. Epub 2020 Feb 13. PMID: 32056051.

230: Yu S, Wang X, He X, Wang Y, Gao S, Ren L, Shi Y. Curcumin exerts anti-

inflammatory and antioxidative properties in 1-methyl-4-phenylpyridinium ion

(MPP(+))-stimulated mesencephalic astrocytes by interference with TLR4 and

downstream signaling pathway. Cell Stress Chaperones. 2016 Jul;21(4):697-705.

doi: 10.1007/s12192-016-0695-3. Epub 2016 May 10. PMID: 27164829; PMCID:

PMC4908001.

231: Yu Z, Yu P, Chen H, Geller HM. Targeted inhibition of KCa3.1 attenuates

TGF-β-induced reactive astrogliosis through the Smad2/3 signaling pathway. J

Neurochem. 2014 Jul;130(1):41-49. doi: 10.1111/jnc.12710. Epub 2014 Mar 27.

PMID: 24606313; PMCID: PMC4065629.

232: Zanon RG, Oliveira AL. MHC I upregulation influences astroglial reaction

and synaptic plasticity in the spinal cord after sciatic nerve transection. Exp

Neurol. 2006 Aug;200(2):521-31. doi: 10.1016/j.expneurol.2006.03.004. Epub 2006

Apr 21. PMID: 16631171.

233: Zhang R, Wu Y, Xie F, Zhong Y, Wang Y, Xu M, Feng J, Charish J, Monnier PP,

Qin X. RGMa mediates reactive astrogliosis and glial scar formation through

TGFβ1/Smad2/3 signaling after stroke. Cell Death Differ. 2018

Aug;25(8):1503-1516. doi: 10.1038/s41418-018-0058-y. Epub 2018 Feb 2. PMID:

29396549; PMCID: PMC6113216.

234: Zhu J, Hu Z, Han X, Wang D, Jiang Q, Ding J, Xiao M, Wang C, Lu M, Hu G.

Dopamine D2 receptor restricts astrocytic NLRP3 inflammasome activation via

enhancing the interaction of β-arrestin2 and NLRP3. Cell Death Differ. 2018

Nov;25(11):2037-2049. doi: 10.1038/s41418-018-0127-2. Epub 2018 May 21. PMID:

29786071; PMCID: PMC6219479.

235: Zou J, Wang YX, Dou FF, Lü HZ, Ma ZW, Lu PH, Xu XM. Glutamine synthetase

down-regulation reduces astrocyte protection against glutamate excitotoxicity to

neurons. Neurochem Int. 2010 Mar;56(4):577-84. doi:

10.1016/j.neuint.2009.12.021. Epub 2010 Jan 12. PMID: 20064572; PMCID:

PMC2831119.
